# The contribution of unstable housing to HIV and hepatitis C virus transmission among people who inject drugs globally, regionally, and at country level: a modelling study

**DOI:** 10.1016/S2468-2667(21)00258-9

**Published:** 2022-01-07

**Authors:** Jack Stone, Adelina Artenie, Matthew Hickman, Natasha K Martin, Louisa Degenhardt, Hannah Fraser, Peter Vickerman

**Affiliations:** Population Health Sciences, Bristol Medical School, University of Bristol, Bristol, UK; Population Health Sciences, Bristol Medical School, University of Bristol, Bristol, UK; Population Health Sciences, Bristol Medical School, University of Bristol, Bristol, UK; NIHR Health Protection Research Unit in Behavioural Science and Evaluation, University of Bristol, Bristol, UK; Division of Global Public Health, University of California San Diego, San Diego, CA, USA; National Drug and Alcohol Research Centre, UNSW Sydney, Sydney, NSW, Australia; Population Health Sciences, Bristol Medical School, University of Bristol, Bristol, UK; Population Health Sciences, Bristol Medical School, University of Bristol, Bristol, UK; NIHR Health Protection Research Unit in Behavioural Science and Evaluation, University of Bristol, Bristol, UK

## Abstract

**Background:**

A considerable proportion of people who inject drugs are unstably housed. Although unstable housing is associated with HIV and HCV infection among people who inject drugs, its contribution to transmission is unknown. We estimated the global and national proportions of incident HIV and HCV infections among people who inject drugs attributed to housing instability from 2020 to 2029.

**Methods:**

In this modelling study, we developed country-level models of unstable housing and HIV and HCV transmission among people who inject drugs in 58 countries globally, calibrated to country-specific data on the prevalences of HIV and HCV and unstable housing. Based on a recently published systematic review, unstably housed people who inject drugs were assumed to have a 39% (95% CI 6–84) increased risk of HIV transmission and a 64% (95% CI 43–89%) increased risk of HCV transmission. We used pooled country-level estimates from systematic reviews on HCV and HIV prevalence in people who inject drugs. Our models estimated the transmission population attributable fraction (tPAF) of unstable housing to HIV and HCV transmission among people who inject drugs, defined as the percentage of infections prevented from 2020 to 2029 if the additional risk due to unstable housing was removed.

**Findings:**

Our models were produced for 58 countries with sufficient data (accounting for >66% of the global people who inject drugs population). Globally, we project unstable housing contributes 7·9% (95% credibility interval [CrI] 2·3–15·7) of new HIV infections and 11·2% (7·7–15·5) of new HCV infections among people who inject drugs from 2020 to 2029. Country-level tPAFs were strongly associated with the prevalence of unstable housing. tPAFs were greater in high-income countries (HIV 17·2% [95% CrI 5·1–30·0]; HCV 19·4% [95% CrI 13·8–26·0]) than in low-income or middle-income countries (HIV 6·6% [95% CrI 1·8–13·1]; HCV 8·3% [95% CrI 5·5–11·7]). tPAFs for HIV and HCV were highest in Afghanistan, Czech Republic, India, USA, England, and Wales where unstable housing contributed more than 20% of new HIV and HCV infections.

**Interpretation:**

Unstable housing is an important modifiable risk factor for HIV and HCV transmission among people who inject drugs in many countries. The study emphasises the importance of implementing initiatives to mitigate these risks and reduce housing instability.

**Funding:**

National Institute for Health Research and National Institute of Allergy and Infectious Diseases and National Institute for Drug Abuse.

## Introduction

Globally, people who inject drugs have a high prevalence of HIV (18%) and more than 50% of people who inject drugs have been infected with hepatitis C virus (HCV).^1^ HIV and HCV prevention interventions for people who inject drugs have typically centred on the delivery of needle and syringe programmes, opioid agonist treatment, and treatment for HIV and HCV. However, there has been growing recognition of the role of social and structural factors and overlapping syndemics in elevating the risk of HIV and HCV transmission among people who inject drugs,^2^ and the need for strategies to address them.^3-7^ Unstable housing, which is typically defined as the lack of access to adequate or fixed housing and includes homelessness, is one of the predominant factors associated with HIV and HCV transmission among people who inject drugs,^5^ along with criminal justice interactions.^3,4,6,7^

It is estimated that, globally, 22% of people who inject drugs have had unstable housing (from here on, we use this term to mean unstable housing or homelessness) in the past year, with this proportion exceeding 40% in India, North America, and some parts of Europe.^1^ A 2021 systematic review found that people who inject drugs who were recently unstably housed were on average at 39% (95% CI 6–84) greater risk of acquiring HIV and 64% (95% CI 43–89%) greater risk of acquiring HCV than people who inject drugs with stable housing.^5^ The mechanisms underlying these relationships are probably multifactorial. Several studies have suggested that the chronic stress associated with meeting daily survival needs among people experiencing unstable housing can supersede efforts to reduce HCV and HIV risk.^8,9^ Unstably housed people who inject drugs face multiple barriers to accessing medical and harm reduction services^10^ and are more likely to engage in sex work and high-risk injecting behaviours than stably housed people who inject drugs,^11,12^ possibly due to the absence of safe places to store sterile injecting equipment, competing priorities in addition to injecting safely, and a greater number of injecting partners.^8,9^ Homelessness has also been linked to HIV outbreaks that occurred during 2011–16 among people who inject drugs in Europe, North America, and Israel.^9^ The current study uses modelling to estimate the global and national proportions (population attributable fraction [PAF]) of incident HIV and HCV infections attributed to housing instability among people who inject drugs in 2020–29.

## Methods

### Model description

In this modelling study, we developed two simple dynamic, deterministic models of HIV or HCV transmission among people who inject drugs globally to account for the chain of transmission events when calculating the PAF of unstable housing (transmission PAF [tPAF]) to HIV and HCV infection. Traditionally, the PAF of a given exposure for a health outcome has been estimated using a simple formula that considers the corresponding relative risk (RR) and prevalence of the exposure (henceforth denoted as the classical PAF [cPAF]).^13^ However, this is not ideal for some infectious diseases because it does not account for the onward chain of transmission resulting from an infection event.^13^ Importantly, the two models we developed were not supposed to capture all intricacies of the HIV and HCV epidemics among people who inject drugs in each country and territory, which would require a more complex model structure and additional data.

**Figure 1:**
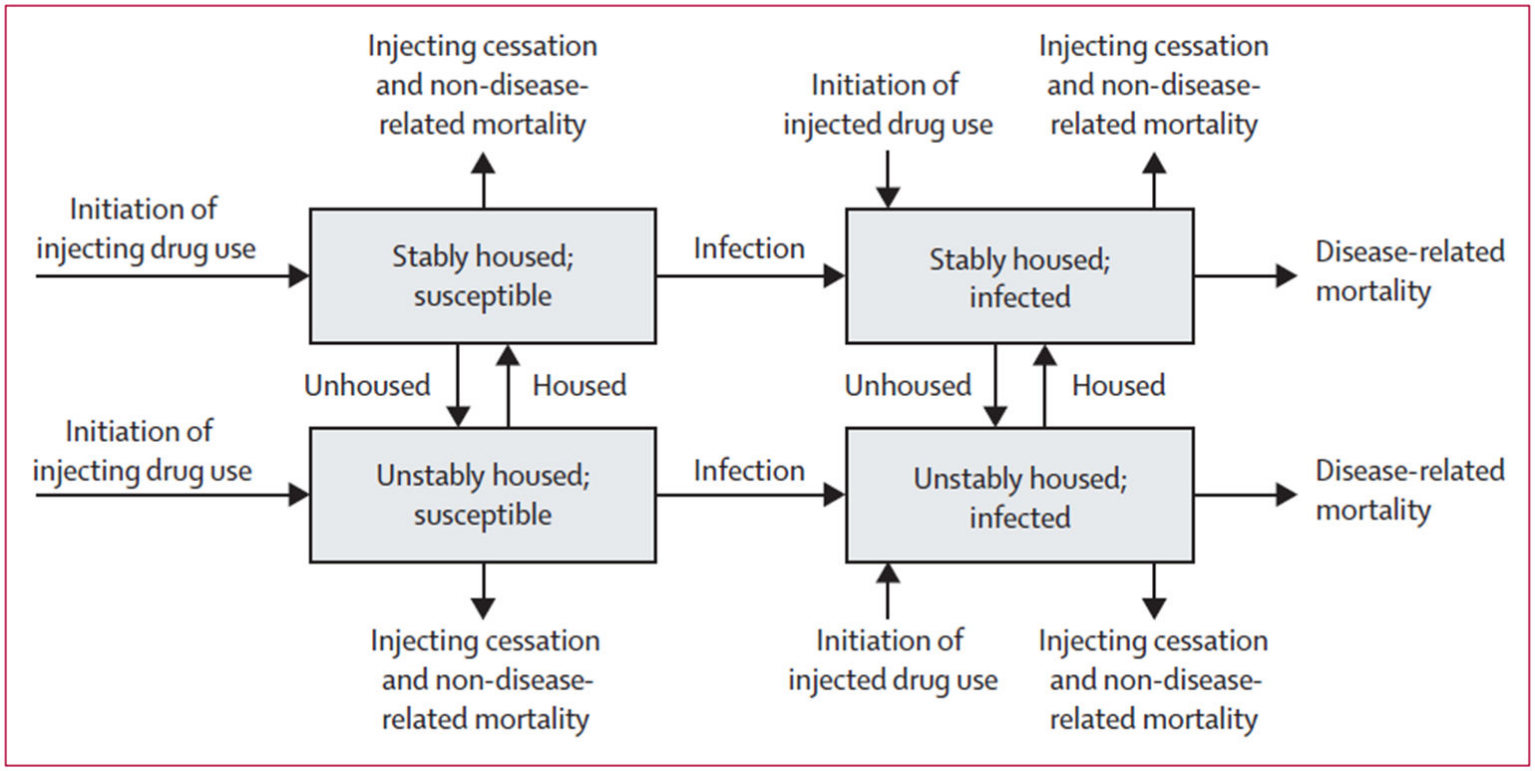
Model schematic for HIV and HCV transmission model among people who inject drugs Disease related mortality only applies for HIV and people who inject drugs can only enter the model as infected when modelling HIV in sub-Saharan Africa. HCV=hepatitis C virus.

Separate models were developed for HIV and HCV (figure 1). Each model stratifies the population of people who inject drugs by infection status (susceptible or infected) and housing status (unstably housed or stably housed). Individuals enter the model through initiating injection drug use, either unstably housed or not. All new people who inject drugs are assumed to be susceptible to HCV or HIV, except in sub-Saharan Africa, where there is a high HIV prevalence in the general population and so individuals can enter the model as infected. We assume entry into the model is balanced by individuals ceasing injection drug use and non-HIV related mortality (which includes overdose and other causes). We include HIV-related mortality in the HIV model, but not HCV-related mortality because it is generally low among people who inject drugs currently.^14^ The effect of HIV treatment was included simply in the HIV model by adjusting the rate of HIV-related mortality to reflect the proportion of HIV-infected people who inject drugs who are on antiretroviral therapy (ART). The rate of HCV treatment is still low among people who inject drugs in most settings or the data are scarce,^15^ and so adjustment by HCV treatment was only included in sensitivity analyses.

In our models, people who inject drugs transition between states of unstable and stable housing at fixed rates to balance each other; the rate is determined by the average duration people who inject drugs are unstably housed. Susceptible people who inject drugs become infected with HIV (with sexual and injecting transmission modelled together but only from other people who inject drugs) or HCV at a rate that depends on the HIV or HCV prevalence among people who inject drugs, with unstably housed people who inject drugs having a greater risk of HIV and HCV acquisition and transmission than stably housed people who inject drugs. The model assumes random mixing.

### Model parameterisation and calibration

We used pooled country-level estimates for countries or territories globally (using only the most recently available data for each country and territory) for people who inject drugs from systematic reviews on HCV antibody prevalence,^1^ HIV prevalence,^1,16^ proportion of individuals that are recently (within the last year) homeless or unstably housed,^1,5^ population size of people who inject drugs,^1,16^ and average duration of injecting drugs (table; appendix pp 3–7). Country-specific estimates are in the appendix (appendix pp 8–12).

HCV antibody prevalence estimates were adjusted to give chronic prevalence, assuming that 22–29% of individuals had spontaneously cleared their infection.^18^ We used the pooled adjusted estimates of the increased risk of HIV (adjusted [a] RR 1·39; 95% CI 1·06–1·84) or HCV (aRR 1·64; 95% CI 1·43–1·89) transmission among unstably housed people who inject drugs from our recent systematic review.^5^

Countries or territories were categorised as having sufficient data for estimating the tPAF of unstable housing to HIV or HCV transmission if they had country-specific estimates of HIV or HCV prevalence and proportion of people who inject drugs that were unstably housed. Regional estimates of the average duration of injecting were used for countries or territories without country-specific data because preliminary analyses showed this parameter had little effect on country-level PAF estimates.

All parameters had uncertainty associated with them, based directly on the studies they were derived from. Given uncertainty in how the current duration of injecting relates to the overall duration of injecting until cessation, considerable uncertainty was associated with this parameter by constructing an uncertainty interval of 50–150% of the median values reported in a 2020 global systematic review and meta-analysis.^17^ Data are scarce on the proportion of individuals in unstable housing when initiating injection drug use. For simplicity, we assumed that the proportion of individuals that are unstably housed when they initiate injecting is the same as the overall proportion of people who inject drugs that are unstably housed in that country or territory. There is also little data on the duration that people who inject drugs remain unstably housed,^19-22^ and so we incorporated wide uncertainty in this parameter (3 months to 2 years) based on estimates from different settings (Scotland, Canada, USA, and Australia^19-22^). Similarly, data on HIV treatment coverage among people who inject drugs is scarce,^24^ and so we assumed it to be between 50–100% (sampled uniformly) of the overall population-level coverage.^25^ Our HIV treatment coverage estimate was used to estimate our average HIV mortality rate for each country or territory by assuming an average survival of 10·8–16·7 years for those not on ART,^26^ with the mortality rate being reduced by 66–80% for those on ART.^27,28^ For sub-Saharan Africa, country-level data on the HIV prevalence among men aged 15–24 years were used as proxy for HIV prevalence when people initiate injecting.

For each country or territory with sufficient data, 1000 parameter sets (including data for calibration) were sampled from their distributions (in effect a probabilistic sensitivity analysis). The models were then separately calibrated for HIV and HCV using the non-linear least-squares fitting function in Matlab version R2020b. For each country or territory and each sampled parameter sets, the HIV or HCV transmission rate and rate that people who inject drugs become unstably housed were calibrated to sampled values for the HIV or chronic HCV prevalence and proportion of people who inject drugs that are unstably housed, assuming that the model was at stability at baseline.

### Model analyses

To estimate the contribution of unstable housing to HIV and HCV transmission, the baseline model fits for each country or territory were run for 10 years from 2020 to 2029, with each being compared with a counterfactual model scenario in which the increased risk of HIV or HCV transmission due to unstable housing was removed (RR=1) over that period. The tPAF of unstable housing was calculated by comparing the number of new infections occurring over 10 years between the baseline model and the counterfactual as:

$$\text{tPAF}=100-100\times \frac{\begin{array}{c} \text{New infections in counterfactual}\\ \text{scenario over 10 years} \end{array}\;\;}{\begin{array}{c} \text{New infections in baseline scenario}\\ \text{over 10 years} \end{array}}$$

The variation across the different model fits for each country or territory were used to produce 95% credibility intervals (CrI). Regional and global estimates of the tPAF were estimated only using those countries or territories with sufficient data and population size estimates for people who inject drugs, with uncertainty incorporated into these estimates. No PAFs were estimated for the Pacific Island States and territories because insufficient data was available. We also estimated the tPAF for high-income countries and low-income and middle-income countries (LMICs).

To investigate within-country heterogeneity, we did a linear regression analysis of covariance to determine which parameter uncertainties contribute most to variability in the country-level tPAFs of unstable housing on HCV or HIV transmission.

To investigate between-country heterogeneity, we used generalised linear regression models to determine which country-level model parameters or calibration outcomes are most important for determining differences in our tPAF estimates across countries or territories. The median 10-year tPAFs for HIV and HCV were regressed on the following covariates: percentage of people who inject drugs that are unstably housed, HCV or HIV prevalence, rate of HIV mortality (HIV tPAF only), rate of non-HIV related mortality, and average duration of injecting.

### Sensitivity analyses

We did sensitivity analyses to investigate how the global and national 10-year tPAF estimates for HIV and HCV would differ if: people who inject drugs mixed partially (25%) assortatively by housing status; all people who inject drugs started injecting as stably housed; the HIV or HCV epidemics were increasing or decreasing (modelled by increasing or decreasing the HIV or HCV transmission rates by 10% from 2020); unadjusted estimates were used for the relative increase in HIV transmission risk if people who inject drugs were unstably housed (RR 1·55; 85% CI 1·23–1·95; this was omitted for HCV because the unadjusted and adjusted estimates were similar^5^); the high relative increase in HCV transmission risk was used for western and eastern European countries (RR 2·06; 95% CI 1·64–2·59;^5^ the only region found to have a statistical difference in the RR for unstable housing^5^); 10% of HCV infected people who inject drugs were treated per year with direct acting antivirals and reinfection occurs at the same rate as for primary infection; the rate of non-HIV mortality for unstably housed people who inject drugs was double that of people who inject drugs who are stably housed.^11^

We also investigated how the regional and global 10-year tPAF estimates for HIV and HCV would differ if we also included countries or territories with insufficient data. For countries or territories with insufficient data, we imputed data for HIV and HCV prevalence or the proportion of individuals that are unstably housed by directly using regional estimates, incorporating uncertainty in these regional estimates from a global systematic review.^1^ Lastly, we also calculated the cPAFs to determine how they differ from the tPAF. The global cPAF was estimated by weighting national cPAFs by the estimated number of prevalent infections among people who inject drugs in each country or territory .

### Role of the funding source

The funders of the study had no role in the study design, data collection, data analysis, data interpretation, or writing of the report.

## Results

**Figure 2:**
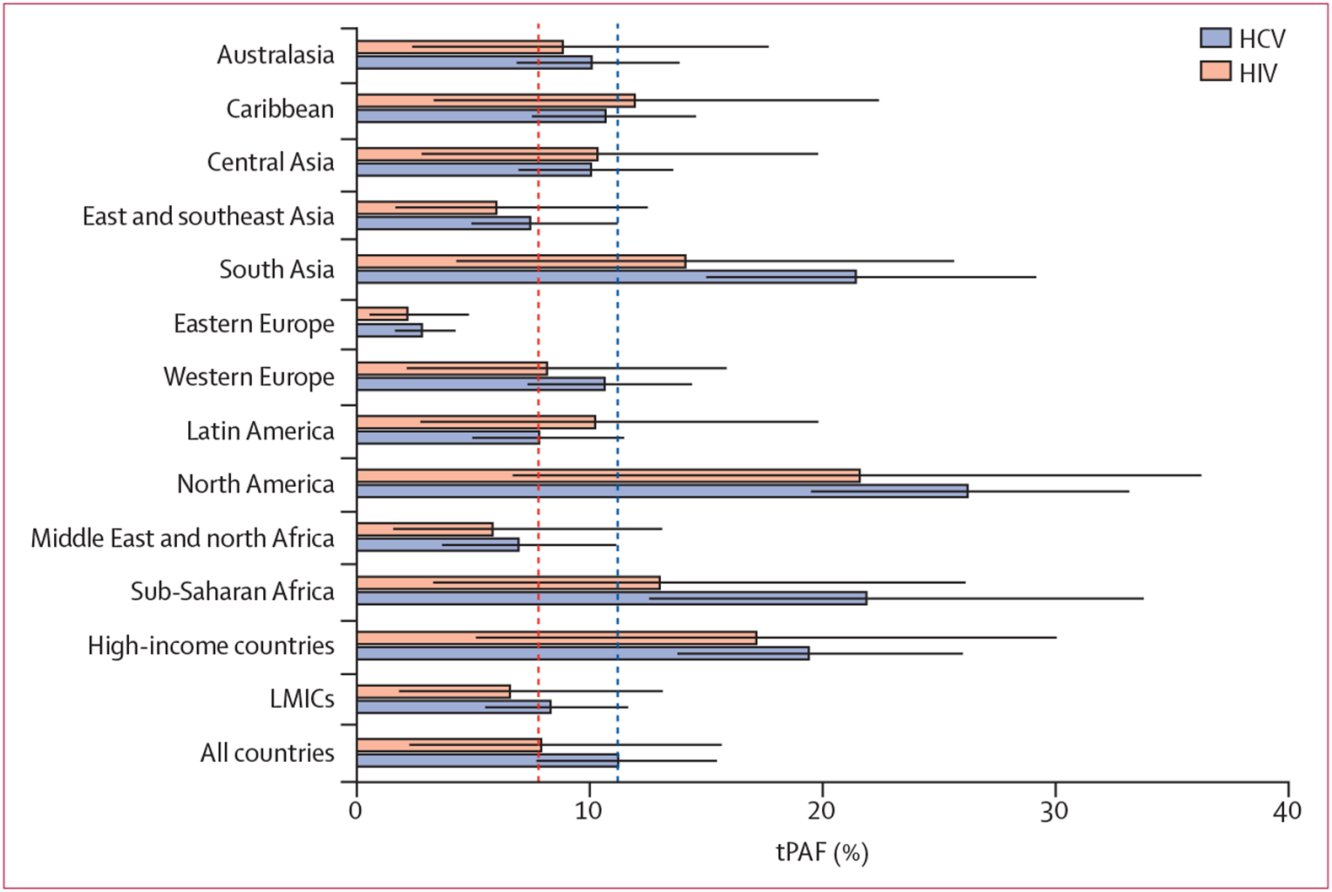
Regional 10-year tPAFs of unstable housing on HIV and HCV among people who inject drugs Estimates are the weighted average over countries and territories within a region with a population size estimate for people who inject drugs, and only includes countries and territories with sufficient data. Red dashed lines show the median global tPAFs for HIV and blue dashed lines show the median global tPAFs for HCV. Whiskers are 95% credibility intervals. HCV=hepatitis C virus. LMIC=low-income and middle-income countries. tPAF=transmission population attributable fraction.

**Figure 3:**
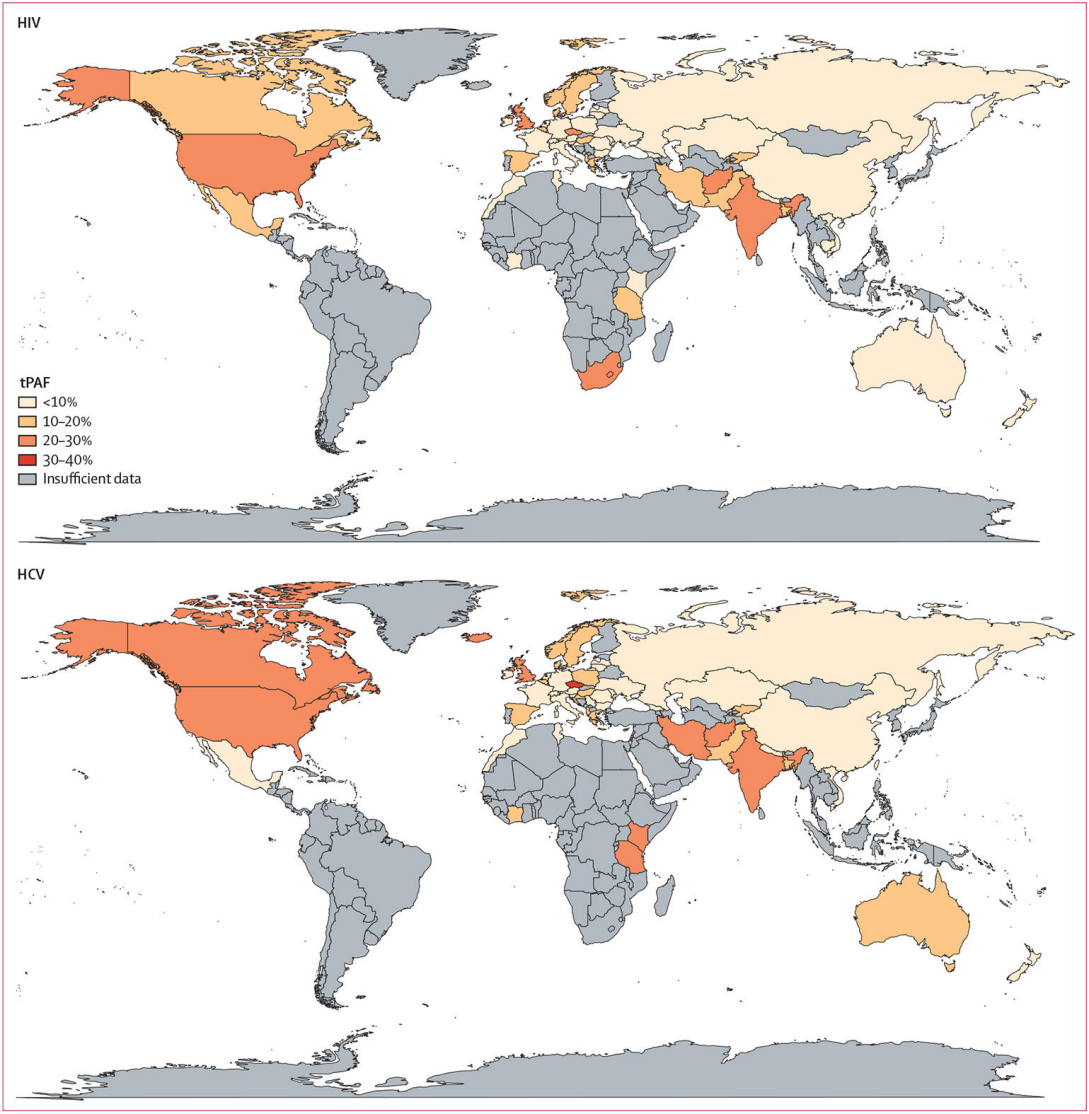
Map of the 10-year tPAFs of unstable housing on HIV and HCV transmission among people who inject drugs Only countries and territories with sufficient data are included. All country-level estimates, including those that have insufficient data and used regional data, are in the appendix (pp 8–12). HCV=hepatitis C virus. tPAF=transmission population attributable fraction.

**Figure 4:**
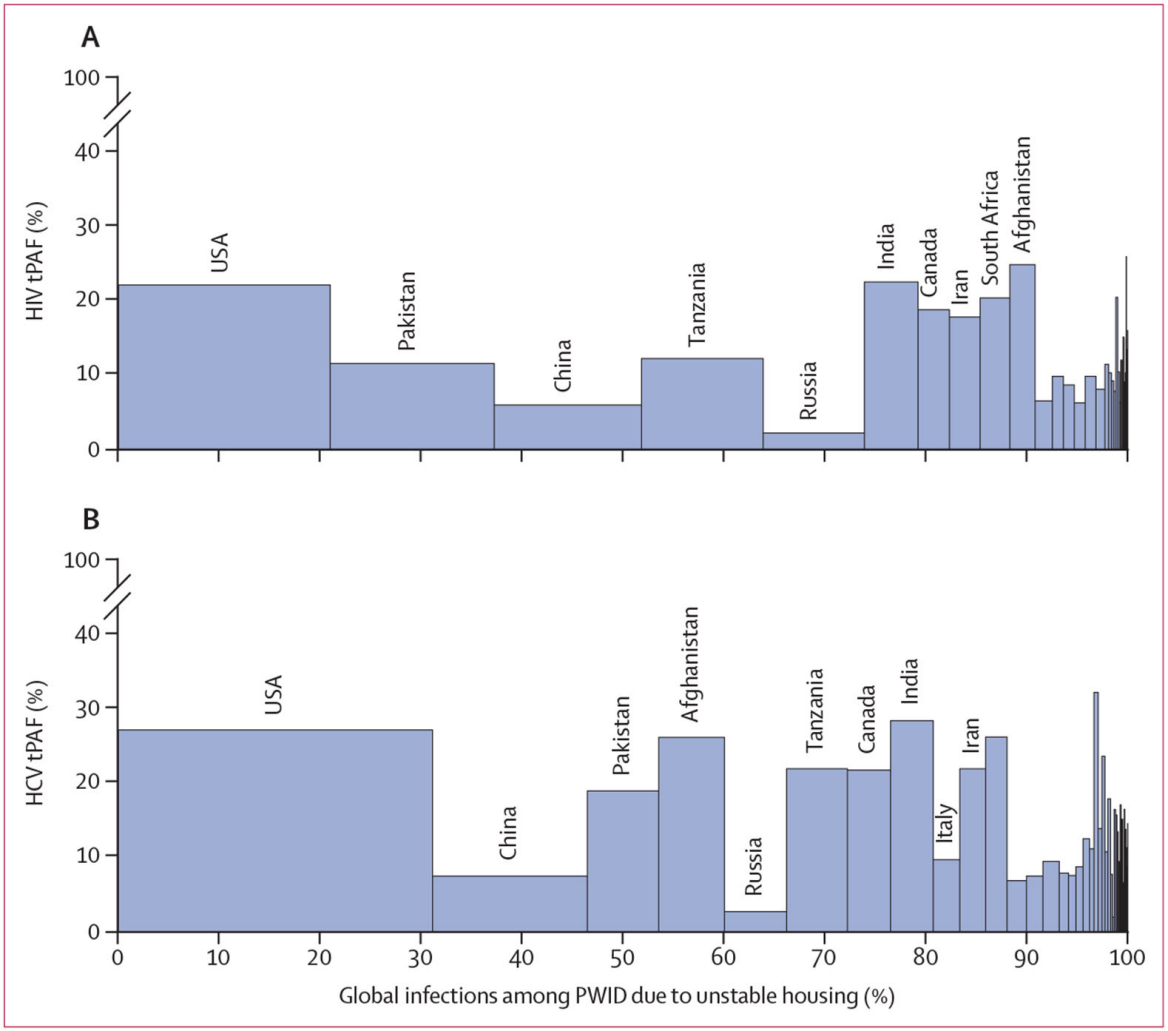
Country-specific estimates for the 10-year tPAF of unstable housing and its contribution to the global number of (A) HIV infections and (B) HCV infections attributed to unstable housing among people who inject drugs The countries with the top 10 largest contributions to the global population attributable fraction are labelled in descending order. Only countries with sufficient data and population size estimates for people who inject drugs are included. HCV=hepatitis C virus. tPAF=transmission population attributable fraction.

Our model projected HIV tPAF estimates for 56 countries or territories and HCV tPAF estimates for 55 countries or territories (58 unique countries or territories overall, mainly from western Europe, eastern Europe, and south Asia), accounting for two-thirds of the world’s population of people who inject drugs. Across all countries or territories with tPAF and population size estimates (HIV n=50, HCV n=49), the model predicts that unstable housing will contribute (tPAF) 7·9% (95% CrI 2·3–15·7) new HIV infections and 11·2% (95% CrI 7·7–15·5) new HCV infections among people who inject drugs between January 2020, and December 2029. There was considerable heterogeneity in tPAFs between regions and countries (figures 2, 3, 4). Across regions, the median tPAFs for HIV ranged from 2·2% (95% CrI 0·5–4·8) in eastern Europe to 21·6% (95% CrI 6·7–36·3) in North America, and tPAFs for HCV ranged from 2·8% (95% CrI 1·6–4·2) in eastern Europe to 26·2% (95% CrI 19·5–33·2) in North America. The other regions in which median tPAFs for HCV were above 20% were sub-Saharan Africa and south Asia. For both HIV and HCV, the tPAFs in high-income countries (HIV 17·2% [95% CrI 5·1–30·0]; HCV 19·4% [95% CrI 13·8–26·0]) were over double those in LMICs (HIV 6·6% [95% CrI 1·8–13·1]; HCV 8·3% [95% CrI 5·5–11·7]), although these differences are largely determined by the tPAFs for USA, China, and Russia.

Across countries or territories with tPAF estimates for both HIV and HCV, tPAFs for HCV were typically higher than for HIV (appendix p 13) because of the greater effect of unstable housing on HCV transmission risk. The highest tPAFs for HIV and HCV were estimated in Afghanistan (HIV 24·7% [95% CrI 7·2–45·0] and HCV 26·0% [95% CrI 17·9–36·3]), Czech Republic (25·8% [95% CrI 8·0–42·1] and 32·0% [95% CrI 24·1–41·1]), India (22·4% [95% CrI 6·6–39·8] and 28·2% [95% CrI 18·5–38·8]), the USA (22·0% [95% CrI 6·9–37·2] and 27·0% [95% CrI 20·1–34·3]), England (20·4% [95% CrI 6·1–35·9] and 26·0% [95% CrI 18·2–34·6]), and Wales (insufficient data for HIV and 30·6% [95% CrI 22·9–38·6]). These six countries contributed 29% of the global HIV and 44% of the global HCV infections attributable to unstable housing among people who inject drugs (estimated as the sum across countries or territories with sufficient data and population size estimates), with the USA being the single largest contributor (figure 4).

Analyses of covariance (appendix pp 15–16) showed that uncertainty in the RR of HIV or HCV transmission if unstably housed and the proportion of people who inject drugs that are unstably housed together contributed over 85·9% (range 85·9–99·3%) of the variability in the tPAF of HIV and 67·6% (49·1–98·9%) of the variability in the tPAF of HCV in all countries or territories except Pakistan. In Pakistan, uncertainty in the HCV prevalence among people who inject drugs (range 0·0–79·1%) was the largest contributor to variability in the tPAF for HCV (41·3%). Across all countries or territories uncertainty in the duration of unstable housing and HIV mortality rate (HIV model only) each contributed less than 1% of the variability in the tPAFs.

**Figure 5:**
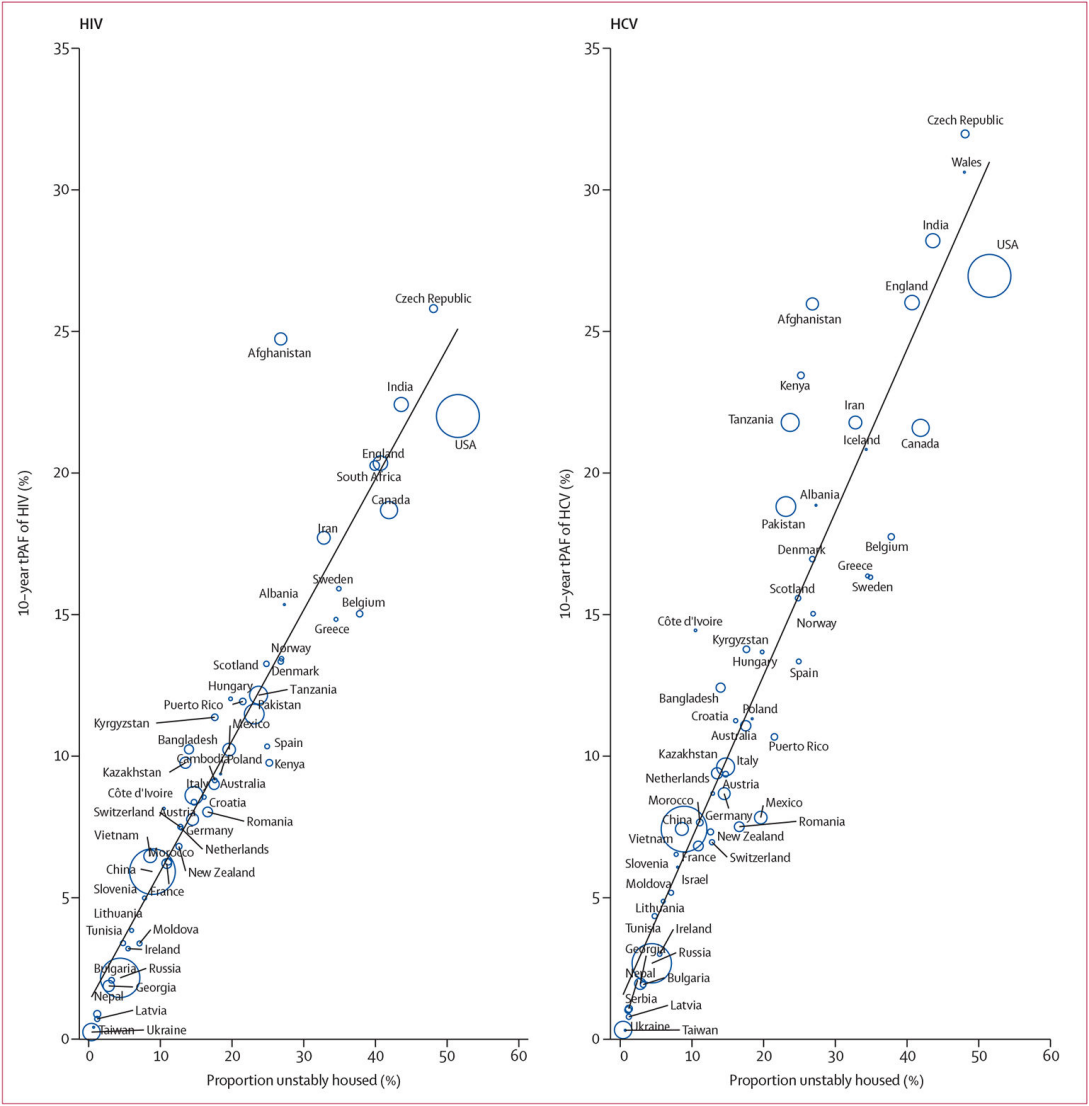
Scatter plot of the associations between each country’s proportion of people who inject drugs that are unstably housed and their median tPAF of unstable housing for HIV and HCV The size of the bubbles is proportional to the population size of people who inject drugs; closed circles for Albania, Iceland, Israel, Poland, Taiwan, and Wales denote missing populations sizes for people who inject drugs. The black line is a plotted line of best fit. HCV=hepatitis C virus. tPAF=transmission population attributable fraction.

For both HIV and HCV, there was a strong positive association between a country or territory’s median tPAF and the proportion of people who inject drugs that are unstably housed (figure 5), with variation in the proportion unstably housed explaining 90·5% of the variability in the median HIV tPAFs and 88·3% of the variability in the median HCV tPAFs. After adjusting for the proportion unstably housed, a country or territory’s prevalence of HIV and chronic HCV were negatively associated with their tPAFs. No other modelled variables were associated with the tPAFs.

In sensitivity analyses (appendix pp 17–30), the global tPAF for HIV (baseline 7·8%) was most sensitive to assuming the high unadjusted RR of HIV transmission if unstably housed (tPAf 11·0% [95% CrI 5·8–18·0]) or partial assortative mixing (tPAF 9·3% [2·7–17·2]). Changes to the RR of HIV transmission had greater effect in countries or territories with higher baseline tPAFs, with the tPAFs increasing to 33·5% ([95% CrI 19·8–46·6) in the Czech Republic, 33·3% (18·3–50·9) in Afghanistan, 29·5% (15·6–44·3) in India, and 29·2% (16·8–41·5) in the USA. Including assortative mixing had the greatest effect in countries or territories with the lowest HIV prevalence (appendix pp 20–29).

The global tPAF for HCV (baseline 11·2%) was most sensitive to assuming the high Europe-specific RR of HCV transmission if unstably housed, which increased the global tPAF to 12·3% (95% CrI 9·1–16·1) and the tPAF for western Europe from 10·6% (95% CrI 7·3–14·4) to 15·9% (11·0–22·0). The effect was greater in European countries with higher tPAFs, such that the tPAFs increased substantially, to 43·9% (95% Crl 32·9–54·4) for Czech Republic, 41·7% (31·8–52·2) for Wales, and 36·8% (25·7–48·6) for England. In contrast to HIV, including assortative mixing generally had little effect on the tPAFs for HCV because of the higher prevalence of HCV than HIV. Regional and national results of the sensitivity analyses are given in the appendix (pp 20–29). Lastly, including countries or territories with imputed data and population size estimates of people who inject drugs resulted in similar global tPAF estimates (appendix p 30).

Overall, we found the cPAF for HIV (5·6% [95%CrI 1·5–10·3]) was 31·0% (95% CrI 20·7 to 39·4) lower than the tPAF, and the cPAF for HCV (10·9% [95% CrI 7·9 to 14·5]) was 3.1% (95% CrI −15·0 to 17·1) higher than the tPAF. For HIV, all country-level cPAFs were smaller than the corresponding tPAFs, whereas this was only the case for HCV when the chronic HCV prevalence was less than 50% (appendix p 14). Indeed, across countries or territories the ratio of the tPAF to the cPAF is negatively associated with disease prevalence (appendix p 14).

## Discussion

Globally, we project that unstable housing will contribute 7·9% of new HIV infections and 11·2% of new HCV infections among people who inject drugs in 2020–29. However, there is considerable variation, with over one-fifth of new HCV and HIV infections being due to this structural exposure in countries or territories with high prevalences (>40%) of unstable housing among people who inject drugs (eg, Czech Republic, India, the USA, and England), but less than 2% in other settings with low prevalences (<3%) of unstable housing among people who inject drugs (eg, Taiwan, Georgia, Latvia, Ukraine, and Nepal). The contribution of unstable housing is also much higher across high-income countries than for LMICs, largely because of substantial differences in the proportion of people who inject drugs who are unstably housed in three key countries: the USA (51·5% unstably housed), China (8·9%), and Russia (4·4%). These differences are likely due to a much higher proportion of people who inject drugs living with family in China (43%^29^) and Russia (up to two-thirds^30^) than in the USA (eg, 3·5% in San Francisco, CA^21^). More generally, differences in the social protection provided by countries is probably an important determinant to variations in the rates of unstable housing across countries,^31^ and so could be an important determinant of the contribution of unstable housing to HIV and HCV transmission among people who inject drugs.

One of the most important strengths of our work is the use of dynamic transmission modelling to estimate the population-level contribution of housing instability to HIV and HCV transmission. Our findings suggest that this contribution might be underestimated using classical methods, particularly for HIV or in settings with lower HCV prevalence (<50%). Our work also exploits data from numerous large-scale systematic reviews.^1,5,17^ Our study has several limitations. Firstly, the estimated PAFs assume a causal relationship between unstable housing and HIV and HCV infection risk, yet the associations derived from our systematic review were based on observational studies, which are liable to confounding. Nevertheless, the associations were robust across numerous scenarios, including among studies with low risk of bias or that had adjusted for important confounders, supporting, at least in part, a causal relationship.^5^ Causality is additionally supported by ample evidence illustrating the potential mechanisms through which risk could be elevated among people who inject drugs who are unstably housed^8,9,11^ and an indication of a dose-response relationship between time unhoused and HCV risk^21^ and of improved HIV treatment outcomes following housing assistance.^12^ In projecting the tPAF, we modelled a complete reduction in the excess risk associated with unstable housing that will probably require a multi-factorial approach beyond simply providing housing.^32^

Secondly, owing to scarce data,^24^ we made some assumptions regarding the ART coverage among people who inject drugs and only modelled its effects on HIV-related mortality. Thirdly, we did not model opioid agonist therapy and needle and syringe programmes or the additional risk of incarceration, all of which might be associated with unstable housing and could confound, mediate, or moderate the effect of unstable housing on HIV and HCV transmission risk. These factors and their relationships are complex with further research being needed to understand how they interact to disentangle the direct and indirect contributions of unstable housing to transmission. Fourth, HCV treatment was only considered in sensitivity analyses. Importantly, we did several sensitivity analyses that collectively suggest that inclusion of these interventions should not have affected our model projections. However, some evidence suggests that housing instability might reduce uptake of HIV and HCV interventions,^10-12^ in which case, our PAF estimates might be conservative. Fifth, the data on how long people who inject drugs stay unstably housed and the proportion of people who inject drugs that are unstably housed when initiating injecting is scarce. Our analyses suggest these uncertainties do not affect our projections. Sixth, aside from a sensitivity analysis done for Europe, we assumed the magnitude of the association between housing instability and HIV and HCV risk to be the same across settings, consistent with findings from our systematic review.^5^ Unfortunately, few studies have estimated this association in LMICs. The definition of unstable housing also differed between settings in our systematic reviews and reflected the sociocultural context in each setting. Lastly, only 58 countries or territories had sufficient data to estimate tPAFs for HIV or HCV, with many estimates based on subnational studies,^1,5,16,17^ which highlights a need to increase the quality and geographical coverage of HIV and HCV surveillance and structural determinants of risk among people who inject drugs.

Few studies have modelled the transmission of infectious diseases in homeless or unstably housed populations, despite this population being highly vulnerable. Additionally, no study has modelled the effects of homelessness on HIV transmission and only two have modelled its effect on HCV transmission; finding that HCV outreach might be cost-effective among homeless people who inject drugs^33^ and that homelessness might contribute substantially to HCV transmission among people who inject drugs in the UK.^34^ The study estimating the contribution of homelessness to HCV transmission in the UK estimated the 15-year tPAF of homelessness in Dundee, Scotland, to be 58% (95% CrI 29–77),^34^ which is much higher than our estimate for Scotland (15·6% [95% CrI 11·2–20·4]). This difference is partly due to the previous study assuming a higher prevalence of homelessness (26·0–42·0%) than our study (20·3–29·3%) and greater risk associated with being homeless (OR 2·13 [range 1·40–3·24]) than our study (aRR 1·64 [95% CI 1·43–1·89]), but also that HCV infection was effectively eliminated through removing the risks associated with homelessness.

Our study is the first to model HIV transmission among unstably housed individuals and to estimate the global contribution of unstable housing to the transmission of any infection. Other studies have estimated the contribution of incarceration to HIV and HCV transmission among people who inject drugs. These studies suggest that the contribution of incarceration to disease transmission might be greater than for unstable housing in the USA (modelled HCV tPAF of incarceration in Kentucky was 42·7% [95% CrI 15·0–67·4]^6^), Ukraine (modelled national HIV tPAF of incarceration is 55·1% [95% CrI 40·2–68·2]^3^), and Scotland (modelled national HCV tPAF of incarceration is 27·7% [95% CrI −3·1 to 51·1%]^7^), although estimates are uncertain.

However, the contribution of incarceration might be lower than the contribution of unstable housing in other settings, depending upon a setting’s incarceration dynamics and whether prison itself is associated with decreased infection risk, thereby offsetting the increased risk following release.^35^

Overall, differences in PAFs across countries or territories point to settings in which strategies are needed to reduce housing instability and associated risk behaviours among people who inject drugs. With the UNAIDS beginning to incorporate social enablers into their target setting, including for key populations,^36^ it is essential to strive for improved access to stable housing for people who inject drugs. Most accommodation-based interventions have been found to be effective for improving housing stability and some health outcomes, particularly if provided without restrictions and coupled with high levels of support and services.^37^ It remains to be established whether such interventions can reduce HIV and HCV risk among people who inject drugs. Given the complex relationship between homelessness, other social determinants of health (including incarceration, poverty, food insecurity, and unemployment), access to harm-reduction programmes, and HIV and HCV risk among people who inject drugs, it is unlikely that stable housing on its own will be sufficient to eliminate the excess risk observed in our study. Interventions must go beyond simply providing stable housing by addressing individuals’ broader health and social needs and providing access to prevention and treatment services.^30^

Our study adds to a small but growing evidence base on the effect of structural factors, such as housing instability and incarceration, on HIV and HCV transmission among people who inject drugs.^3-7^ Efforts towards HIV and HCV elimination should not overlook the importance of implementing interventions and policies that address the structural drivers of risk in this group. In settings where unstable housing and other structural factors contribute considerably to transmission (eg, USA and UK), HIV and HCV elimination targets will be missed unless the effect of these structural drivers are mitigated.

## Supplementary Material

1

## Figures and Tables

**Table:** Model parameters and calibration data

	Sampling Distribution	Reference
HIV prevalence among people who inject drugs	Triangular distributions that differ by country or territory	^1,16^
HCV antibody prevalence among people who inject drugs	Triangular distributions that differ by country or territory	^1^
Population size of people who inject drugs	Triangular distributions that differ by country or territory	^1,16^
Proportion of people who inject drugs that are unstably housed	Triangular distributions that differ by country or territory	^1^
Average duration of injecting	Triangular distributions that differ by country or territory	^17^
Proportion of HCV infections that spontaneously clear	Uniform (0·22–0·29%)	^18^
Relative increase in HIV transmission risk if unstably housed	Lognormal distribution with mean 1·39 (95% CI 1·06–1·84)	^5^
Relative increase in HCV transmission risk if unstably housed	Lognormal distribution with mean 1·64 (95% CI 1·43–1·89)	^5^
Average duration of unstable housing (years)	Uniform (0·25–2)	^19-22^
Annual rate of non-HIV mortality*	Triangular distributions that differ by country, region, or territory	^23^
Average number of years before HIV mortality	Differs by country depending on country-level ART coverage	^25-27^

ART=antiretroviral therapy. HCV=hepatitis C virus.

* Regional estimates used as available; otherwise estimates for high-income and low-income and middle-income countries were used as appropriate.

## Research in Context

### Evidence before this study

We anticipated few studies to have estimated the contribution of homelessness or unstable housing to HIV and hepatitis C virus (HCV) transmission, and therefore we used a broad search strategy that did not focus on these two infectious diseases. We searched PubMed on June 11, 2021, with no date or language restrictions, for published studies that either estimated the population attributable fraction (PAF) of unstable housing or homelessness on health outcomes through modelling or any other methods or, more generally, modelled infectious disease transmission among unstably housed or homeless individuals. We used the search terms: {([math* OR “dynamic” OR “transmission”] AND [model*]) OR [“population attributable fraction” OR “tPAF” OR “PAF”]} AND {([“stable” OR “unstable” OR “stability” OR “instability”] AND hous*) OR homeless*}. We identified four studies that evaluated the classical PAF (not model based so not accounting for changes in transmission dynamics) of: homelessness on tuberculosis infections (two studies), homelessness on the unsuccessful outcomes of treatment of newly diagnosed pulmonary tuberculosis cases (one study), and severe housing insecurity during pregnancy on birth and infant outcomes (one study). We identified only 11 studies that modelled infectious disease transmission among unstably housed or homeless individuals: five for tuberculosis, four for COVID-19, and two for HCV. Only two of these studies considered homelessness among people who inject drugs, both of which modelled HCV transmission in the UK. The first of these studies found an outreach intervention to improve HCV testing and treatment uptake among homeless people who inject drugs in London, England, to be cost effective, and possibly cost saving. The other study estimated the 15-year transmission population attributable fraction (tPAF; which accounts for changes in transmission dynamics) of homelessness to HCV transmission among people who inject drugs in Dundee, Scotland (58%) or homelessness and injecting crack cocaine in Walsall, England (59%) and Bristol, England (64%).

### Added value of the study

Our study is the first to model HIV transmission among unstably housed individuals and only the second to estimate the tPAF of unstable housing to the transmission of any infection. Using data from several systematic reviews, our study explores the contribution of unstable housing to HIV and HCV transmission among people who inject drugs globally and shows that this risk factor accounts for a substantial proportion of incident HIV and HCV cases in many countries. We highlight the countries and regions in which a large proportion of new HIV or HCV infection cases are attributed to housing instability and therefore where housing-oriented strategies are most needed to reduce transmission and to strengthen progress towards reaching HIV and HCV elimination targets.

### Implications of all the available evidence

Although unstable housing and homelessness are known social determinants of health, few studies have considered their potential contribution to disease transmission or the potential effect and cost-effectiveness of targeting prevention and treatment interventions to people experiencing housing instability. WHO calls the 2020s the decade for disease elimination and recommends countries should eliminate HIV and HCV as public health threats by 2030. Given that people who inject drugs are a central population to HIV and HCV elimination goals, and that, in some countries, housing instability contributes substantially to transmission in this group, elimination efforts should expand to also address the broader social needs of people who inject drugs. Although further work is required to develop and implement interventions that best serve these needs, such interventions could have important population-level effects and be cost-effective. Rapid action taken by some national governments to house people who are homeless during the COVID-19 pandemic has shown that bold programmes to reduce housing instability are possible.
