# Supplementary material for: The contribution of unstable housing to HIV and hepatitis C virus transmission among people who inject drugs globally, regionally, and at country level: a modelling study
Source: Lancet Public Health. Author manuscript; Available in PMC 2022 Feb 16. (PMC8848679; doi:10.1016/S2468-2667(21)00258-9)
Supplement: 1 [file NIHMS1777140-supplement-1.pdf]

THE LANCET
Public Health

Supplementary appendix

This appendix formed part of the original submission and has been peer reviewed. We post it as supplied by the authors.

Supplement to: Stone J, Artenie A, Hickman M, et al. The contribution of unstable housing to HIV and hepatitis C virus transmission among people who inject drugs globally, regionally, and at country level: a modelling study. *Lancet Public Health* 2022; published online Jan 7. https://doi.org/10.1016/S2468-2667(21)00258-9.

Model Equations 2
Appendix Table 1: Data values used for each country, and their sources. 3
Appendix Table 2: Country-level tPAFs of Unstable Housing on HIV and HCV 8
Appendix Figure 1: Scatter plot of the tPAFs of HIV and HCV 13
Appendix Figure 2: Scatter plot of the ratio between the median tPAF and cPAF and the HIV/HCV prevalence 14
Appendix Figure 3: Results of the uncertainty analyses for the PAF of Unstable Housing on HIV transmission 15
Appendix Figure 4: Results of the uncertainty analyses for the PAF of Unstable Housing on HCV transmission. 16
Appendix Table 3: Sensitivity analysis for the Regional & Global tPAFs of Unstable Housing on HIV 17
Appendix Table 4: Sensitivity analysis for the Regional & Global tPAFs of Unstable Housing on HCV 18
Appendix Table 5: Regional & Global tPAFs of Unstable Housing on HIV and HCV including countries with imputed data 19
Appendix Table 6: Sensitivity analysis for the country-level tPAFs of Unstable Housing on HIV. 20
Appendix Table 7: Sensitivity analysis for the tPAFs of Unstable Housing on HCV 25
Appendix Figure 5: Sensitivity analyses. 30
References 31

Model Equations

Let S_k and I_k denote the number of PWID that are susceptible or infected over time, with subscript k denoting strata for not homeless ($k = 0$) or currently homeless ($k = 1$). Then the model can be described by the following equations:

$$\frac{dS_0}{dt} = (1-\rho_I)(1-\rho_H)\theta - (\beta + \kappa + \mu)S_0 + \epsilon S_1$$
$$\frac{dS_1}{dt} = (1-\rho_I)\rho_H\theta - (\eta\beta + \epsilon + \mu)S_1 + \kappa S_1$$
$$\frac{dI_0}{dt} = \rho_I(1-\rho_H)\theta + \beta S_0 - (\mu_I + \mu + \kappa)I_0 + \epsilon I_1$$
$$\frac{dI_1}{dt} = \rho_I\rho_H\theta + \eta\beta S_1 - (\mu_I + \mu + \epsilon)I_1 + \kappa I_0$$

Where,

- θ is the number of individuals that enter the model each year.
- ρ_H is the proportion of individuals that are unstably housed when initiating injecting
- ρ_I is the proportion of individuals that are infected when initiating injecting
- κ is the rate at which PWID become unstably housed.
- ϵ is the rate at which unstably housed PWID stop being unstably housed.
- μ is the rate at which PWID cease injecting or die from non-disease related causes.
- μ_I is the rate at which infected PWID die from disease related causes. $\mu_I = 0$ when modelling HCV.
- η is the relative transmission risk if unstably housed compared to stably housed.
- And β is the force of infection given by:

$$\beta = \frac{I_0 + \eta I_1}{(S_0 + I_0) + \eta(S_1 + I_1)}$$

Appendix Table 1: Data values used for each country, and their sources.

Country	PWID Population size	Ref	HIV Prevalence	Ref	HCV AB Prevalence	Ref	% Unstably Housed	Ref	Injecting duration	Ref
**Australasia**	115500 (83000-148000)	1	1.1% (0.8-1.4)	1	57.1% (52.7-61.5)	1	16.5% (11.4-22.1)	1	15.4	2
Australia	93000 (68000-118000)	1	1.3% (1-1.6)	1	53.5% (50.2-56.9)	1	17.5% (12.5-22.5)	1	15.4	2
New Zealand	22500 (15000-30000)	1	0.1% (0-0.8)	1	71.9% (63.2-80.6)	1	12.6% (6.9-20.6)	1		
**Caribbean**	79500 (53000-118000)	1	13.5% (8.3-19.1)	1	63.6% (54.3-72.6)	1	21.5% (17.5-25.5)	1	12.9	2
Antigua & Barbuda										
Bahamas										
Barbados										
Bermuda										
Comm. of Puerto Rico	28000 (19000-42000)	1	6% (3.7-9.3)	1	78.4% (73.5-82.8)	1	21.5% (17.5-25.5)	1	12.9	2
Cuba										
Dominica										
Dominican Republic										
Grenada										
Haiti										
Jamaica										
Saint Kitts & Nevis										
Saint Lucia										
St Vincent & the Grenadines										
Trinidad & Tobago										
**Central Asia**	281500 (189500-416500)	1	10.5% (8.6-12.5)	1	38.6% (17.2-62.4)	1	14.3% (11.7-17.2)	1	5.7	2
Kazakhstan	112500 (75500-166000)	1	9.2% (8-10.4)	1	58.8% (54-63.6)	1	13.5% (10.9-16.5)	1	5	2
Kyrgyzstan	28500 (19000-42000)	1	12.4% (10.3-14.7)	1	43.9% (40.6-47.2)	1	17.6% (15.1-20.2)	1	6.3	2
Tajikistan	23500 (16000-34500)	1	27% (21-33.7)	1	61.3% (56.8-65.6)	1			5.9	2
Turkmenistan										
Uzbekistan	94000 (63500-139000)	1	7.3% (5.8-9.1)	1	51.7% (46.8-56.6)	1				
**East and South East Asia**	3989000 (3041000-49555000)	1	15.2% (9.9-20.4)	1	50.3% (37.7-62.8)	1	8.8% (5.9-12.9)	1	7.4	2
Brunei Darussalam										
Cambodia	10500 (9500-22500)	1	24.4% (17-33.1)	1			17.6% (14.4-20.8)	1		
China	2564000 (1964000-3164000)	1	12.4% (6.8-17.9)	1	43.1% (27.5-58.6)	1	8.9% (6.3-12.2)	1	7.1	2
Hong Kong (China)					85.4% (82.2-88.2)	1			17	2
Indonesia	190500 (156000-225000)	1	44.5% (34-55)	1	89.2% (85.3-92.3)	1			7.1	2
Japan	368500 (281000-459000)	1			64.8% (55-74.5)	1				
Lao PDR	1661		17.4% (7.8-31.4)	1						
Malaysia	281500 (233500-330000)	1	17.8% (16.6-19.1)	1	67.1% (62.9-71.1)	1			13.9	2
Mongolia										
Myanmar	173500 (115500-235000)	1	23.4% (19-27.7)	1	29.5% (26.9-32.2)	1			3.4	2
North Korea										
Philippines	25500 (19000-32000)	1	20.3% (13-27.6)	1	35.2% (15.9-54.5)	1			6.8	2
Republic of Korea			0% (0-0)	1	48.4% (42.8-54.1)	1			4.8	2
Singapore					42.5% (39.1-45.9)	1				
Taiwan			12.4% (8.1-16.8)	1	91% (89.5-92.4)	1	0.7% (0.3-1.3)	1	15.5	2
Thailand	51500 (16000-87000)	1	24.5% (17.4-31.7)	1	88.5% (82.6-92.9)	1				
Timor Leste	500 (500-500)	1								

Country	PWID Population size	Ref	HIV Prevalence	Ref	HCV AB Prevalence	Ref	% Unstably Housed	Ref	Injecting duration	Ref
Viet Nam	161000 (123000-200500)	1	16.6% (13.1-20.1)	1	58.3% (42.7-74)	1	8.6% (0.3-26.1)	1	5.8	2
**Eastern Europe**	3020000 (1653500-5008000)	1	24.7% (15.6-33.9)	1	64.7% (56.6-72.9)	1	6.7% (4.4-9.2)	1	8.7	2
Armenia	13000 (9000-29000)	1	5.4% (2.2-8.5)	1	42.7% (29.3-56.1)	1				
Azerbaijan	43500 (34500-52000)	1	9.7% (5.6-13.8)	1	62.1% (47.1-77.2)	1			8.8	2
Belarus	40500 (15000-66000)	1	25.6% (17.9-33.2)	1	58.3% (43.3-73.3)	1			10.9	2
Bosnia & Herzegovina			0.3% (0-0.6)	1	39.9% (27.5-52.4)	1			15	2
Bulgaria	18500 (15000-22500)	1	7% (3.2-10.8)	1	68.7% (64.3-73)	1	3.2% (2.4-3.9)	1	9	2
Czech Republic	47000 (44500-49000)	1	0.3% (0.2-0.4)	1	18.3% (14.5-22.1)	1	48.1% (41.9-54.9)	1		
Estonia	8500 (6500-16000)	1	53.4% (44.4-62.5)	1	79.2% (67.4-91)	1			8.1	2
Georgia	115000 (13000-217000)	1	2.2% (1.5-2.9)	1	69.1% (58-80.2)	1	2.8% (1-5.9)	1	14.1	2
Hungary	4000 (2000-6000)	1	0.2% (0-0.4)	1	46.6% (30.4-62.8)	1	19.8% (6.6-32.9)	1	9.6	2
Latvia	14000 (11000-18000)	1	26.9% (24.1-29.6)	1	74.4% (67.6-81.2)	1	1.2% (0.4-2.8)	1	9.1	2
Lithuania	5000 (2500-8000)	1	8% (1.7-14.4)	1	41.1% (38.1-44.2)	1	6% (3.9-8.8)	1	10	2
Moldova	12000 (7500-16500)	1	29.5% (12.9-46)	1	50.1% (34.1-66.1)	1	7.1% (4.7-10.4)	1	12.7	2
Poland			18% (15.3-20.9)	1	58.7% (55.1-62.2)	1	18.4% (16.2-20.5)	1	14.4	2
Romania	81500 (60500-110000)	1	20.5% (7-34.1)	1	83.8% (80.6-87.1)	1	16.6% (1.9-31.3)	1	9.8	2
Russian Federation	1881000 (1028500-3114000)	1	30.4% (17.9-43)	1	68.7% (59.6-77.9)	1	4.4% (2.2-6.6)	1	7.6	2
Slovakia	20000 (14500-36000)	1	0.01% (0-0.03)	1	56.1% (35.6-76.7)	1				
Ukraine	319500 (172000-590500)	1	19.1% (16.1-22.2)	1	53.9% (49.2-58.7)	1	0.4% (0.2-0.8)	1	12.2	2
**Latin America**	1823000 (1392000-2380000)	1	35.7% (15-56.6)	1	61.9% (58.9-64.9)	1	19.6% (13-26.3)	1	16.1	2
Argentina	80500 (79000-82500)	1	49.7% (35.4-64)	1	54.6% (51.1-58.1)	1				
Belize										
Bolivia										
Brazil	962000 (734500-1256000)	1	48% (18-78)	1	63.9% (60.5-67.3)	1				
Chile	47000 (36000-61500)	1								
Colombia			4.6% (2.7-6.4)	1	28.8% (26.2-31.6)	1			3	2
Costa Rica										
Ecuador										
El Salvador										
Guatemala										
Guyana										
Honduras										
Mexico	150500 (100500-209500)	1	4% (3-4.9)	1	95.3% (93.3-97.3)	1	19.6% (13-26.3)	1	16.1	2
Nicaragua			2.4% (0.1-12.9)	1					6	2
Panama										
Paraguay			9.4% (3.7-15)	1	9.8% (7.7-11.9)	1				
Peru			13% (10.9-15.1)	1						
Suriname										
Uruguay	6500 (2000-19000)	1	18.5% (16.1-21)	1	21.9% (19-24.8)	1				
Venezuela										
**Middle East & North Africa**	349500 (177500-521500)	1	3.6% (1.5-6.2)	1	48.1% (39.2-57.1)	1	9.4% (4.3-14.8)	1	10	2
Algeria	40961 (26333-55590)	3	1.1% (0-5.7)	1						
Bahrain	674 (337-1011)	3	4.6% (1.9-9.3)	1						
Cyprus	500 (500-1000)	1	1.2% (0.6-1.7)	1	49.7% (44.4-55)	1			8.8	2
Egypt	88618 (56970-120265)	3	2.6% (0.6-4.5)	1	49.4% (35.8-63)	1				
Iraq	34673 (23115-46230)	3								

Country	PWID Population size	Ref	HIV Prevalence	Ref	HCV AB Prevalence	Ref	% Unstably Housed	Ref	Injecting duration	Ref
Israel			0% (0-0)	1	45.3% (38.1-52.6)	1	8% (4.7-12.7)	1	14	2
Jordan	4850 (3200-6500)	3								
Kuwait	4100 (2700-5500)	3								
Lebanon	3300 (2200-4400)	3	0% (0-0)	1	23.4% (15.3-33.3)	1				
Libyan Arab Jamahiriya	2000 (1000-3000)	1	89.6% (85.8-92.7)	1	94.5% (91.5-96.7)	1				
Morocco	30500 (15500-45500)	1	9.6% (0-20.6)	1	53.9% (33.7-74)	1	11.1% (4.6-17.7)	1	10	2
Occ. Palestinian Territories	1850 (1200-2500)	3	0% (0-0)	1	41.6% (35.2-47.9)	1			14	2
Oman	4250 (2800-5700)	3	11.8% (5-18.6)	1						
Qatar	1190 (780-1600)	3								
Saudi Arabia	23600 (15172-32028)	3	9.8% (7-13.2)	1	77.8% (73.2-81.9)	1				
South Sudan										
Sudan	37828 (24319-51337)	3	0% (0-0)	1						
Syrian Arab Rep.	6000 (4000-8000)	3	0% (0-0)	1	3.3% (1.8-5.6)	1				
Tunisia	13163 (8462-17864)	3	3.5% (2.6-4.4)	1	29.1% (25.7-32.6)	1	4.8% (3.3-6.6)	1		
Turkey			0.2% (0.1-0.4)	1	44.9% (41.7-48.2)	1			4	2
United Arab Emirates	4800 (3200-6400)	3								
Yemen	19770 (12710-26830)	3								
**North America**	2557000 (1498500-4428000)	1	9% (7-11.1)	1	55.2% (40.8-67.7)	1	50.3% (39.7-61)	1	16	2
Canada	308000 (262000-354500)	1	11.3% (8.5-14.3)	1	65.4% (60.1-70.6)	1	41.9% (30.6-53.1)	1	14.3	2
United States	2248500 (1236500-4074000)	1	8.7% (6.8-10.7)	1	53.1% (38.1-68)	1	51.5% (40.9-62.1)	1	16.2	2
**Pacific Island States & Territories**	22500 (15000-33500)	1	16.3% (10-22.7)	1	55.5% (43.8-67)	1				
American Samoa										
Fed. States of Micronesia										
Fiji										
French Polynesia										
Guam										
Kiribati										
Marshall Islands										
Nauru										
New Caledonia										
Northern Mariana Islands										
Palau										
Papua New Guinea										
Samoa			0% (0-0)	1						
Solomon Islands			0% (0-0)	1						
Tonga			0% (0-0)	1						
Tuvalu										
Vanuatu										
**South Asia**	1023500 (783500-1263000)	1	19.4% (15-23.8)	1	38.6% (17.2-62.4)	1	27.8% (19-36.7)	1	5.7	2
Afghanistan	139000 (88000-190500)	1	4% (2.2-5.8)	1	37.8% (27.5-48.1)	1	26.8% (22.5-31.4)	4	2.5	2
Bangladesh	68500 (63500-74000)	1	0.5% (0.2-0.7)	1	33.9% (22.4-45.4)	1	14% (4.6-23.3)	1	6	2
Bhutan										
India	197000 (127500-267000)	1	15.6% (12.9-18.2)	1	40% (33.9-46.1)	1	43.6% (25.4-61.9)	1	7.2	2
Iran	158000 (107000-209000)	1	14% (9.2-18.7)	1	44.1% (28.2-59.9)	1	32.8% (23.5-42.1)	1	8.2	2

Country	PWID Population size	Ref	HIV Prevalence	Ref	HCV AB Prevalence	Ref	% Unstably Housed	Ref	Injecting duration	Ref
Maldives	1500 (500-2500)	1	0% (0-0)	1	0.7% (0-1.7)	1			3.5	2
Nepal	35000 (33500-37000)	1	9.6% (6.3-12.9)	1	44.5% (30.8-58.2)	1	1.2% (0.4-2)	1	5.2	2
Pakistan	423000 (363000-482500)	1	32.3% (25.5-39.1)	1	36.5% (0-79.1)	1	23.1% (18.3-28)	1	5.1	2
Sri Lanka	500 (500-500)	1	0% (0-0)	1			2.1% (0.9-4.4)	1	11	2
**Sub Saharan Africa**	1378000 (645500-3080000)	1	18.3% (11.3-25.4)	1	21.8% (17.6-26.5)	1	26.5% (12-41)	1	4.6	2
Angola			5.1% (3.2-7)	1						
Benin									13	2
Botswana										
Burkina Faso										
Burundi										
Cameroon										
Cape Verde										
Central African Republic										
Chad										
Comoros										
Congo (Kinshasa)	3500 (0-158000)	1	13.3% (7.3-21.6)	1						
Cote d'Ivoire	500 (500-1000)	1	5.3% (1.1-14.6)	1	1.8% (0-9.4)	1	10.5% (4.0-21.5)	1		
Djibouti										
Equatorial Guinea										
Eritrea										
Ethiopia										
Gabon										
Gambia										
Ghana					40.1% (34.8-45.4)	1			10	2
Guinea										
Guinea-Bissau										
Kenya	30500 (9000-52000)	1	42% (21.1-62.8)	1	16.4% (10.9-23.3)	1	25.2% (24.1-26.3)	4	5.3	2
Lesotho										
Liberia										
Madagascar	15500 (3000-79500)	1	4.8% (0.2-9.4)	1	5.5% (2.1-9)	1				
Malawi										
Mali										
Mauritania										
Mauritius	7000 (3500-14000)	1	45.5% (42.4-48.6)	1	97.1% (96-98.1)	1			14	2
Mozambique	29000 (0-59000)	1	46.3% (41.9-50.7)	1	67.1% (62.9-71.2)	1				
Namibia										
Niger										
Nigeria			3.1% (1.8-4.4)	1	5.8% (3.8-8.9)	1			8	2
Rep. of the Congo										
Rwanda	2000 (500-4500)	1								
Sao Tome & Principe										
Senegal			9.3% (5-15.4)	1	39.3% (31.1-47.9)	1				
Seychelles	1500 (1000-2500)	1	3.8% (2-6.4)	1	42% (36.8-47.4)	1				
Sierra Leone	1500 (1000-1500)	1	8.5% (5.4-12.6)	1						
Somalia	1000 (500-2000)	1								
South Africa	76000 (21500-268000)	1	14.2% (11.1-17.8)	1			39.9% (21.1-58.7)	1		
Swaziland										

Country	PWID Population size	Ref	HIV Prevalence	Ref	HCV AB Prevalence	Ref	% Unstably Housed	Ref	Injecting duration	Ref
Togo	2500 (500-19500)	[1]					13% (9.2-17.6)	[1]		
Uganda										
United Rep. of Tanzania	343000 (200000-486000)	[1]	28.3% (16.3-40.4)	[1]	27.7% (22.4-33.5)	[1]	23.7% (10-37.3)	[1]	4.3	[2]
Zambia										
Zimbabwe										
**Western Europe**	1009500 (686500-1386500)	[1]	4.5% (3.2-6)	[1]	53.2% (48.4-57.9)	[1]	21.9% (15.9-27.9)	[1]	10.7	[2]
Albania			0.5% (0-2.8)	[1]	34% (27.5-41)	[1]	27.3% (19.6-36.1)	[1]		
Andorra										
Austria	18500 (12500-24500)	[1]	0.6% (0.3-1)	[1]	60.9% (54.8-67)	[1]	14.7% (9.6-21.3)	[1]	13	[2]
Belgium	26000 (18500-37000)	[1]	4.3% (3.3-5.4)	[1]	58.4% (47-69.7)	[1]	37.8% (15.4-40.2)	[1]	13.3	[2]
Croatia	6500 (5000-8500)	[1]	0.4% (0-0.8)	[1]	36.7% (28.1-45.3)	[1]	16.1% (8.6-23.7)	[1]	13.5	[2]
Denmark	16500 (13000-19000)	[1]	1.3% (0.3-3.6)	[1]	42.6% (36.1-49.1)	[1]	26.8% (14.2-42.9)	[1]	18.2	[2]
England	210500 (196500-225000)	[1]	0.8% (0.1-1.5)	[1]	30.7% (26.9-34.6)	[1]	40.7% (26.4-55)	[1]	10	[2]
Finland	17000 (15000-25000)	[1]	1.2% (0.5-2.4)	[1]	73.7% (69.9-77.2)	[1]				
Form. Yug. Rep. Macedonia					62.2% (59.4-64.9)	[1]				
France	82000 (66500-970000)	[1]	8.7% (5.3-12.1)	[1]	64% (60.8-67)	[1]	10.9% (7.2-15.8)	[4]		
Germany	131500 (14000-249500)	[1]	4.4% (2.3-6.4)	[1]	65% (60.6-69.4)	[1]	14.5% (10.7-18.2)	[1]	13.9	[2]
Greece	5000 (4000-6000)	[1]	6.9% (4.2-9.6)	[1]	65.7% (61.8-69.5)	[1]	34.5% (18.2-36)	[1]	11.7	[2]
Greenland										
Iceland					63% (59.8-66.2)	[1]	34.3% (33.1-44)	[1]	7	[2]
Ireland	8500 (6500-10500)	[1]	5.8% (4.2-7.4)	[1]	74.6% (72.3-76.9)	[1]	5.5% (3.4-7.6)	[1]		
Italy	341500 (233500-467500)	[1]	6.1% (4.7-7.5)	[1]	57.9% (52.5-63.3)	[1]	14.7% (13.5-16)	[1]	9	[2]
Liechtenstein										
Luxembourg	2000 (1500-2500)	[1]	1.5% (0.5-2.5)	[1]	81.3% (76.2-85.8)	[1]				
Malta			0.5% (0-1.2)	[1]	25.2% (13.1-37.3)	[1]				
Monaco										
Montenegro			0.2% (0-0.6)	[1]	43.4% (39.8-47.1)	[1]			6	[2]
Netherlands	3500 (2500-4500)	[1]	2.3% (1.9-2.6)	[1]	55.3% (49.7-60.9)	[1]	12.9% (10.5-15.6)	[4]		
Northern Ireland										
Norway	8500 (7000-10000)	[1]	0.7% (0-1.5)	[1]	64.8% (60.4-69.1)	[1]	26.9% (16.9-36.9)	[1]	14	[2]
Portugal	16000 (14000-17500)	[1]	18% (15.4-20.6)	[1]	87.7% (80.5-95)	[1]				
San Marino										
Scotland	16000 (13500-17500)	[1]	0.8% (0.5-1.3)	[1]	52.2% (45.5-58.8)	[1]	24.8% (20.3-29.3)	[1]	11.5	[2]
Serbia	29000 (24000-34500)	[1]	0% (0-0)	[1]	25.9% (22.1-29.7)	[1]	1.1% (0.2-2)	[1]	8.8	[2]
Slovenia	6000 (4000-75000)	[1]	0.5% (0.1-1)	[1]	30.5% (26.4-34.5)	[1]	7.8% (4.5-12.4)	[1]		
Spain	10500 (3500-17500)	[1]	32.6% (31.6-33.6)	[1]	71% (69.5-72.5)	[1]	24.9% (15.4-34.4)	[1]	11.2	[2]
Sweden	8000 (2000-38500)	[1]	0.2% (0-0.5)	[1]	81.7% (79.6-83.6)	[1]	34.9% (21.2-48.7)	[1]	21	[2]
Switzerland	13500 (11000-16000)	[1]	1.4% (0.6-2.2)	[1]	74.6% (69.3-79.4)	[1]	12.8% (11-14.7)	[1]		
Wales					26.8% (23.4-30.4)	[1]	48.0% (43.6-52.5)	[4]	9	[2]

Appendix Table 2: Country-level tPAFs of Unstable Housing on HIV and HCV. *Estimates marked with an asterisk denotes that the country had 'insufficient data' and so regional data for were used.*

	HIV tPAF	HCV tPAF
**Australasia**		
Australia	9.0% (2.4-17.9)	11.1% (7.3-15.1)
New Zealand	6.8% (1.9-14.4)	7.3% (4.2-12.0)
**Caribbean**		
Antigua & Barbuda	11.3% (3.2-21.4) *	12.6% (9.0-16.6) *
Bahamas	11.2% (3.1-21.2) *	12.6% (8.9-16.9) *
Barbados	11.3% (3.2-21.4) *	12.6% (8.7-16.9) *
Bermuda	11.0% (3.2-21.3) *	12.7% (9.0-16.8) *
Comm. of Puerto Rico	11.9% (3.3-22.4)	10.7% (7.5-14.6)
Cuba	11.1% (3.1-21.1) *	12.6% (8.9-17.0) *
Dominica	11.2% (3.1-21.3) *	12.6% (9.0-16.7) *
Dominican Republic	11.4% (3.1-20.8) *	12.7% (9.0-16.7) *
Grenada	11.3% (3.1-21.0) *	12.5% (8.9-17.1) *
Haiti	11.2% (3.0-20.7) *	12.5% (8.8-16.8) *
Jamaica	11.4% (3.1-20.8) *	12.7% (9.0-17.0) *
Saint Kitts & Nevis	11.2% (3.1-20.8) *	12.6% (8.9-17.1) *
Saint Lucia	11.2% (3.3-21.6) *	12.7% (8.9-16.9) *
St Vincent & the Grenadines	11.2% (3.3-21.2) *	12.7% (9.0-17.0) *
Trinidad & Tobago	10.9% (3.1-21.0) *	12.5% (8.8-16.7) *
**Central Asia**		
Kazakhstan	9.8% (2.6-19.8)	9.4% (6.6-12.9)
Kyrgyzstan	11.4% (3.2-21.3)	13.8% (9.6-18.7)
Tajikistan	7.7% (2.1-15.7) *	9.4% (6.5-12.7) *
Turkmenistan	10.0% (2.6-19.8) *	12.1% (8.1-17.3) *
Uzbekistan	10.1% (2.6-20.0) *	10.7% (7.5-14.8) *
**East and South East Asia**		
Brunei Darussalam	5.6% (1.4-12.3) *	6.9% (4.4-10.5) *
Cambodia	9.1% (2.5-17.8)	12.6% (8.8-17.1) *
China	5.9% (1.6-12.3)	7.4% (4.9-11.4)
Hong Kong (China)	4.9% (1.2-10.0) *	4.3% (2.7-6.5) *
Indonesia	3.5% (0.9-7.4) *	3.5% (2.2-5.4) *
Japan	5.8% (1.5-11.7) *	5.7% (3.6-9.0) *
Lao PDR	5.4% (1.4-11.3) *	6.8% (4.4-10.5) *
Malaysia	5.0% (1.3-10.5) *	5.5% (3.4-8.1) *
Mongolia	5.8% (1.5-12.4) *	6.9% (4.4-10.6) *
Myanmar	6.0% (1.6-13.2) *	10.3% (6.7-16.1) *
North Korea	5.6% (1.4-12.3) *	6.9% (4.3-10.4) *
Philippines	5.4% (1.5-11.6) *	8.1% (4.9-12.4) *
Republic of Korea	N/A	7.5% (4.6-11.5) *
Singapore	5.6% (1.5-11.9) *	7.4% (4.7-11.5) *
Taiwan	0.4% (0.1-1.1)	0.3% (0.2-0.6)
Thailand	5.0% (1.2-10.5) *	3.7% (2.3-5.9) *
Timor Leste	5.6% (1.4-12.4) *	6.8% (4.3-10.2) *
Viet Nam	6.5% (0.9-18.1)	7.4% (1.6-16.7)
**Eastern Europe**		

Armenia	4.4% (1.1-9.3) *	5.3% (3.5-8.0) *
Azerbaijan	4.2% (1.1-9.1) *	4.3% (2.8-6.6) *
Belarus	3.4% (0.9-7.1) *	4.5% (2.8-6.8) *
Bosnia & Herzegovina	4.1% (1.0-8.9) *	5.1% (3.3-7.7) *
Bulgaria	2.1% (0.5-4.6)	1.9% (1.3-2.7)
Czech Republic	25.8% (8.0-42.1)	32.0% (24.1-41.1)
Estonia	2.1% (0.6-4.5) *	3.4% (2.1-5.3) *
Georgia	1.9% (0.5-5.1)	2.0% (0.9-3.6)
Hungary	12.0% (3.1-25.3)	13.7% (6.7-21.4)
Latvia	0.7% (0.2-2.0)	0.8% (0.3-1.5)
Lithuania	3.8% (1.0-8.5)	4.9% (3.1-7.4)
Moldova	3.4% (0.9-7.8)	5.2% (3.2-7.8)
Poland	9.4% (2.6-17.7)	11.3% (8.2-15.0)
Romania	8.0% (1.4-20.5)	7.5% (2.3-13.6)
Russian Federation	2.2% (0.5-5.0)	2.7% (1.4-4.2)
Slovakia	4.7% (1.1-10.4) *	4.7% (2.9-7.1) *
Ukraine	0.3% (0.1-0.6)	0.3% (0.2-0.6)
**Latin America**		
Argentina	6.4% (1.9-13.4) *	12.1% (7.8-16.9) *
Belize	7.8% (2.0-16.1) *	11.6% (7.6-16.3) *
Bolivia	7.6% (2.1-16.0) *	11.4% (7.7-16.3) *
Brazil	6.5% (1.6-14.6) *	11.4% (7.6-16.0) *
Chile	7.5% (2.1-15.5) *	11.5% (7.6-16.3) *
Colombia	16.8% (4.9-33.5) *	20.9% (13.3-30.9) *
Costa Rica	7.9% (2.0-15.8) *	11.6% (7.5-16.0) *
Ecuador	7.7% (2.0-16.1) *	11.5% (7.5-15.8) *
El Salvador	7.8% (2.0-15.7) *	11.5% (7.6-16.5) *
Guatemala	7.8% (2.0-16.2) *	11.5% (7.6-16.2) *
Guyana	7.7% (2.2-16.1) *	11.4% (7.4-16.1) *
Honduras	7.8% (2.2-15.4) *	11.4% (7.8-16.1) *
Mexico	10.2% (2.7-19.8)	7.8% (5.0-11.5)
Nicaragua	12.7% (3.4-25.2) *	12.0% (8.1-17.4) *
Panama	7.7% (2.0-15.7) *	11.4% (7.6-16.1) *
Paraguay	9.8% (2.7-18.6) *	14.0% (9.3-19.8) *
Peru	9.5% (2.6-18.4) *	11.5% (7.6-16.0) *
Suriname	7.7% (2.2-15.6) *	11.5% (7.7-16.0) *
Uruguay	9.2% (2.5-17.8) *	13.6% (9.0-19.8) *
Venezuela	7.8% (2.2-15.9) *	11.5% (7.6-16.4) *
**Middle East & North Africa**		
Algeria	6.0% (1.5-13.3) *	7.0% (3.8-11.1) *
Bahrain	5.9% (1.6-13.5) *	6.9% (3.7-10.9) *
Cyprus	6.2% (1.7-14.5) *	7.0% (3.8-11.1) *
Egypt	6.0% (1.5-14.0) *	6.9% (3.8-11.1) *
Iraq	6.0% (1.5-13.8) *	7.1% (4.0-11.2) *
Israel	NA	6.1% (3.7-9.5)
Jordan	6.0% (1.5-13.5) *	7.0% (3.7-10.9) *
Kuwait	6.0% (1.6-13.5) *	6.9% (3.8-11.0) *
Lebanon	NA	8.3% (4.8-13.1) *
Libyan Arab Jamahiriya	0.4% (0.1-1.0) *	3.4% (1.7-5.7) *

Morocco	6.3% (1.7-14.4)	7.7% (3.7-12.5)
Occ. Palestinian Territories	NA	7.0% (3.8-11.0) *
Oman	5.4% (1.4-12.2) *	6.8% (3.8-11.1) *
Qatar	6.0% (1.4-14.1) *	6.9% (3.6-11.0) *
Saudi Arabia	5.8% (1.5-12.9) *	4.9% (2.8-7.8) *
South Sudan	6.1% (1.5-14.1) *	6.9% (3.8-11.2) *
Sudan	NA	6.8% (3.7-11.1) *
Syrian Arab Rep.	NA	9.0% (5.0-14.5) *
Tunisia	3.4% (0.9-7.3)	4.4% (2.7-6.5)
Turkey	8.2% (2.0-18.9) *	8.3% (4.5-13.4) *
United Arab Emirates	5.9% (1.5-14.0) *	7.0% (3.8-10.9) *
Yemen	6.0% (1.5-14.1) *	6.9% (3.6-11.2) *
**North America**		
Canada	18.7% (5.7-33.0)	21.6% (15.4-28.8)
United States	22.0% (6.9-37.2)	27.0% (20.1-34.3)
**South Asia**		
Afghanistan	24.7% (7.2-45.0)	26.0% (17.9-36.3)
Bangladesh	10.2% (2.4-23.4)	12.4% (5.7-20.6)
Bhutan	15.6% (4.5-29.1) *	21.3% (13.8-30.3) *
India	22.4% (6.6-39.8)	28.2% (18.5-38.8)
Iran	17.7% (5.1-32.8)	21.8% (15.3-29.7)
Maldives	NA	32.6% (22.3-46.1) *
Nepal	0.9% (0.2-2.3)	1.1% (0.5-2.0)
Pakistan	11.5% (3.2-22.5)	18.8% (11.5-28.3)
Sri Lanka	NA	2.0% (1.0-3.7) *
**Sub Saharan Africa**		
Angola	17.4% (4.6-34.7) *	24.3% (13.8-36.1) *
Benin	12.6% (3.5-24.5) *	19.3% (10.9-28.4) *
Botswana	13.1% (3.4-26.1) *	24.5% (14.6-35.1) *
Burkina Faso	15.1% (4.2-32.0) *	24.7% (14.3-36.2) *
Burundi	15.1% (4.0-30.8) *	24.6% (13.9-36.8) *
Cameroon	15.3% (4.2-31.1) *	24.1% (14.4-37.3) *
Cape Verde	15.2% (4.3-31.5) *	24.3% (13.9-37.0) *
Central African Republic	15.0% (4.2-30.7) *	24.3% (14.2-37.1) *
Chad	15.4% (4.2-30.9) *	24.4% (14.3-37.0) *
Comoros	15.6% (4.0-30.6) *	24.4% (14.3-37.1) *
Congo (Kinshasa)	16.7% (5.1-34.6) *	24.7% (14.4-35.9) *
Cote d'Ivoire	8.1% (1.9-19.5)	14.4% (6.8-25.5)
Djibouti	15.2% (4.5-31.2) *	24.4% (14.5-36.3) *
Equatorial Guinea	14.8% (4.2-30.2) *	24.6% (14.5-37.0) *
Eritrea	15.4% (4.3-32.8) *	24.4% (14.3-37.0) *
Ethiopia	15.2% (4.2-31.3) *	24.2% (14.0-37.0) *
Gabon	15.5% (4.1-31.1) *	24.5% (14.0-36.2) *
Gambia	15.7% (4.5-32.3) *	24.5% (14.2-36.3) *
Ghana	13.1% (3.5-26.5) *	18.3% (10.5-27.7) *
Guinea	15.8% (4.5-31.2) *	24.3% (14.5-36.3) *
Guinea-Bissau	15.4% (4.2-31.1) *	24.5% (14.4-36.1) *
Kenya	9.8% (2.5-20.6)	23.4% (17.1-31.8)
Lesotho	13.2% (3.6-26.4) *	24.2% (13.6-36.9) *

Liberia	15.4% (4.4-32.1) *	24.4% (14.5-35.5) *
Madagascar	18.4% (5.4-36.8) *	26.7% (15.5-40.2) *
Malawi	14.3% (3.8-29.6) *	24.3% (14.1-37.4) *
Mali	15.8% (4.3-31.4) *	24.8% (14.7-36.1) *
Mauritania	15.5% (4.1-31.4) *	24.2% (14.5-36.3) *
Mauritius	9.2% (2.4-19.8) *	9.4% (4.8-14.7) *
Mozambique	9.4% (2.5-20.2) *	15.0% (8.6-23.3) *
Namibia	13.9% (3.9-29.4) *	24.4% (14.8-36.9) *
Niger	15.9% (3.9-31.4) *	24.5% (14.2-36.6) *
Nigeria	14.6% (3.9-30.1) *	22.5% (13.3-33.6) *
Rep. of the Congo	15.6% (4.2-31.6) *	24.3% (14.1-36.6) *
Rwanda	14.9% (4.1-30.9) *	24.3% (13.7-36.0) *
Sao Tome & Principe	15.7% (4.2-31.7) *	24.6% (14.5-37.2) *
Senegal	16.8% (4.4-34.6) *	21.1% (11.5-31.8) *
Seychelles	17.9% (4.8-37.2) *	20.8% (12.1-31.9) *
Sierra Leone	17.3% (4.6-33.5) *	24.8% (14.3-35.9) *
Somalia	16.1% (4.1-31.1) *	24.5% (14.0-36.5) *
South Africa	20.3% (5.8-36.8)	33.1% (21.7-46.1) *
Swaziland	13.4% (3.7-26.5) *	24.5% (13.9-37.4) *
Togo	8.4% (2.2-17.1) *	14.1% (9.0-20.7) *
Uganda	14.8% (3.9-30.6) *	24.4% (14.5-36.2) *
United Rep. of Tanzania	12.2% (3.0-26.5)	21.8% (12.1-34.0)
Zambia	13.9% (3.7-28.7) *	24.6% (14.4-36.3) *
Zimbabwe	13.9% (3.6-27.9) *	24.7% (14.5-35.5) *
**Western Europe**		
Albania	15.4% (4.3-28.0)	18.9% (13.0-25.3)
Andorra	11.8% (3.3-22.7) *	13.9% (9.4-19.1) *
Austria	8.4% (2.2-16.9)	9.4% (5.8-13.7)
Belgium	15.0% (4.1-29.1)	17.7% (10.7-24.8)
Croatia	8.6% (2.2-18.4)	11.3% (6.7-16.8)
Denmark	13.3% (3.8-26.6)	17.0% (10.4-25.1)
England	20.4% (6.1-35.9)	26.0% (18.2-34.6)
Finland	12.0% (3.4-22.8) *	11.4% (7.9-15.9) *
Form. Yug. Rep. Macedonia	11.7% (3.4-22.7) *	12.9% (8.9-17.9) *
France	6.2% (1.6-12.9)	6.8% (4.4-10.3)
Germany	7.8% (2.1-15.7)	8.7% (6.0-12.2)
Greece	14.8% (4.4-27.7)	16.4% (10.5-22.1)
Greenland	11.7% (3.2-22.9) *	13.9% (9.7-19.1) *
Iceland	20.8% (6.3-36.6) *	20.8% (15.2-27.1)
Ireland	3.2% (0.8-7.0)	3.0% (1.9-4.6)
Italy	8.6% (2.4-17.1)	9.6% (6.9-12.7)
Liechtenstein	11.8% (3.4-22.9) *	14.0% (9.7-19.2) *
Luxembourg	12.1% (3.5-23.1) *	10.3% (7.2-14.7) *
Malta	12.2% (3.3-23.5) *	16.3% (11.2-22.5) *
Monaco	11.8% (3.3-23.4) *	14.0% (9.6-19.3) *
Montenegro	14.7% (4.0-28.3) *	16.3% (11.1-22.6) *
Netherlands	7.5% (2.1-14.7)	8.7% (6.0-11.6)
Northern Ireland	12.0% (3.2-22.6) *	13.9% (9.6-19.2) *
Norway	13.4% (3.9-26.5)	15.0% (9.7-20.8)

Portugal	10.7% (3.0-20.8) *	9.2% (6.1-13.2) *
San Marino	11.8% (3.3-22.9) *	13.8% (9.4-19.4) *
Scotland	13.3% (3.5-24.7)	15.6% (11.2-20.4)
Serbia	NA	1.0% (0.4-1.8)
Slovenia	5.0% (1.2-11.2)	6.5% (3.8-10.6)
Spain	10.3% (3.0-20.7)	13.3% (8.6-19.1)
Sweden	15.9% (4.6-29.7)	16.3% (10.6-23.5)
Switzerland	7.5% (2.0-15.0)	7.0% (5.0-9.3)
Wales	23.7% (7.2-39.7) *	30.6% (22.9-38.6)

Appendix Figure 1: Scatter plot of the tPAFs of HIV and HCV for countries where sufficient data was available for both estimates. Dashed line is y=x.

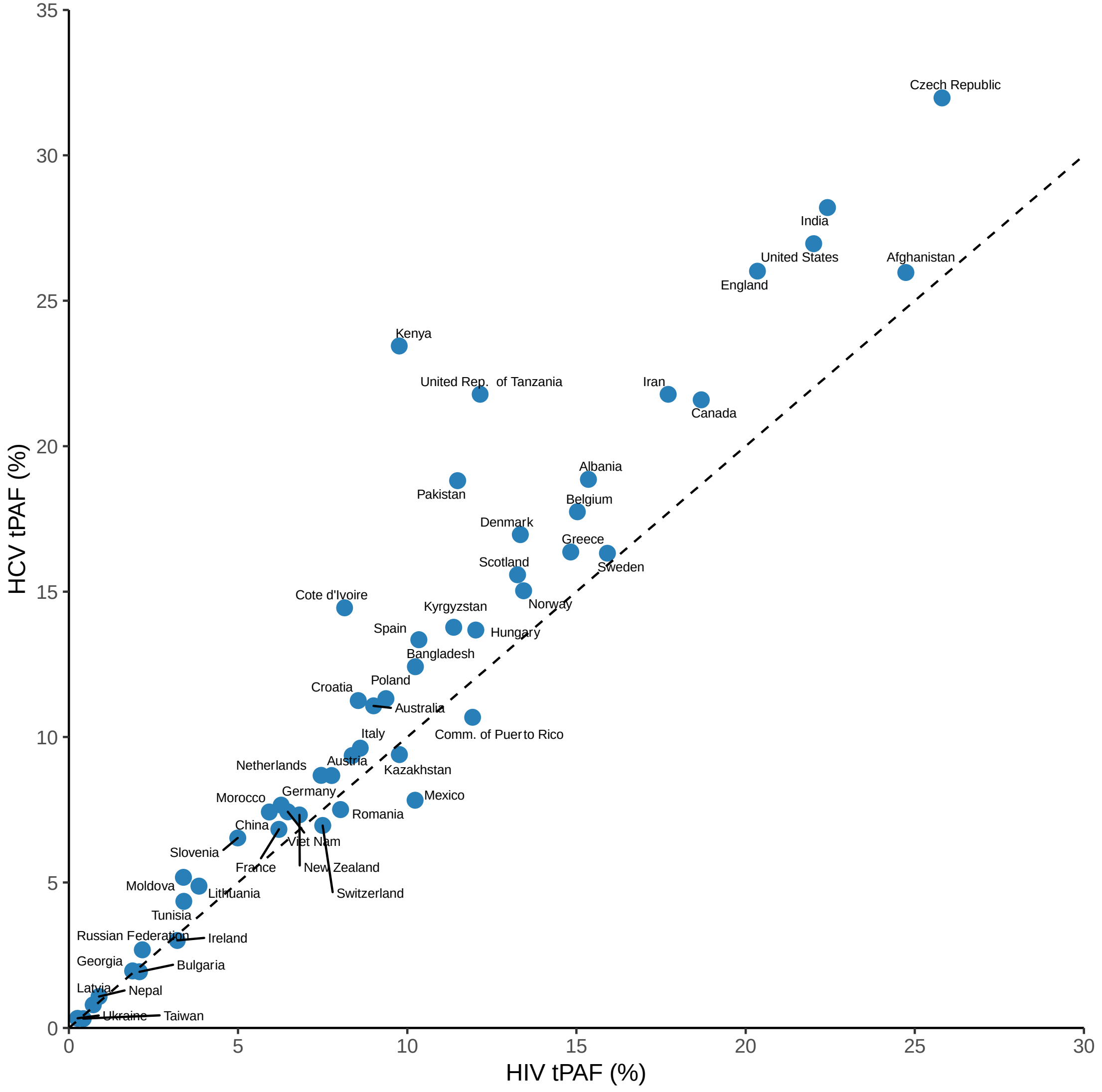

Appendix Figure 2: Scatter plot of the ratio between the median tPAF and cPAF and the HIV/HCV prevalence for countries where sufficient data was available. Black lines are the line of best fit.

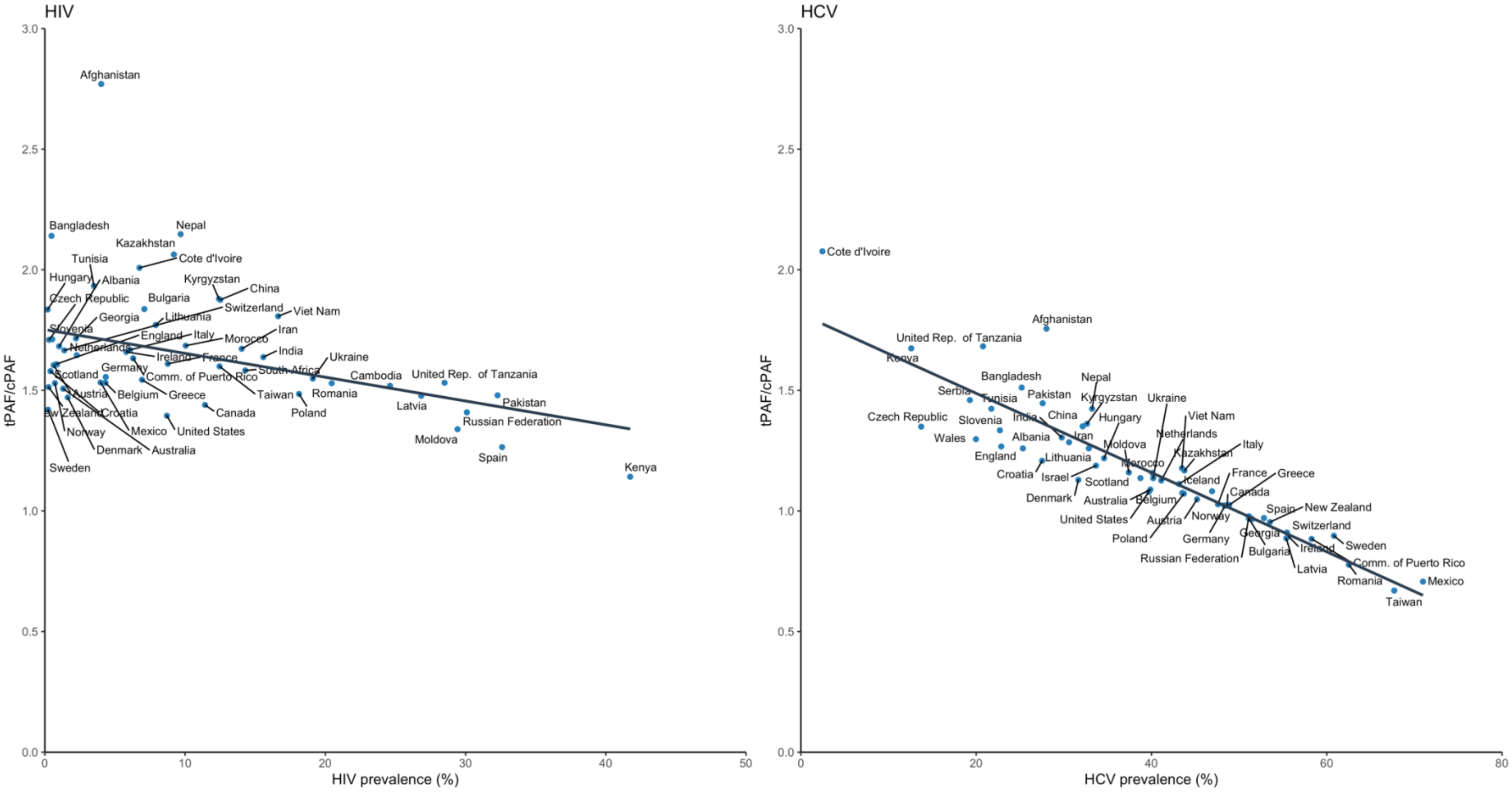

Appendix Figure 3: Results of the uncertainty analyses for the PAF of Unstable Housing on HIV transmission. Stacked bars show how uncertainty in each parameter contributed to the variability in the estimates of the PAF for each country for which the PAF could be estimated without using imputed data.

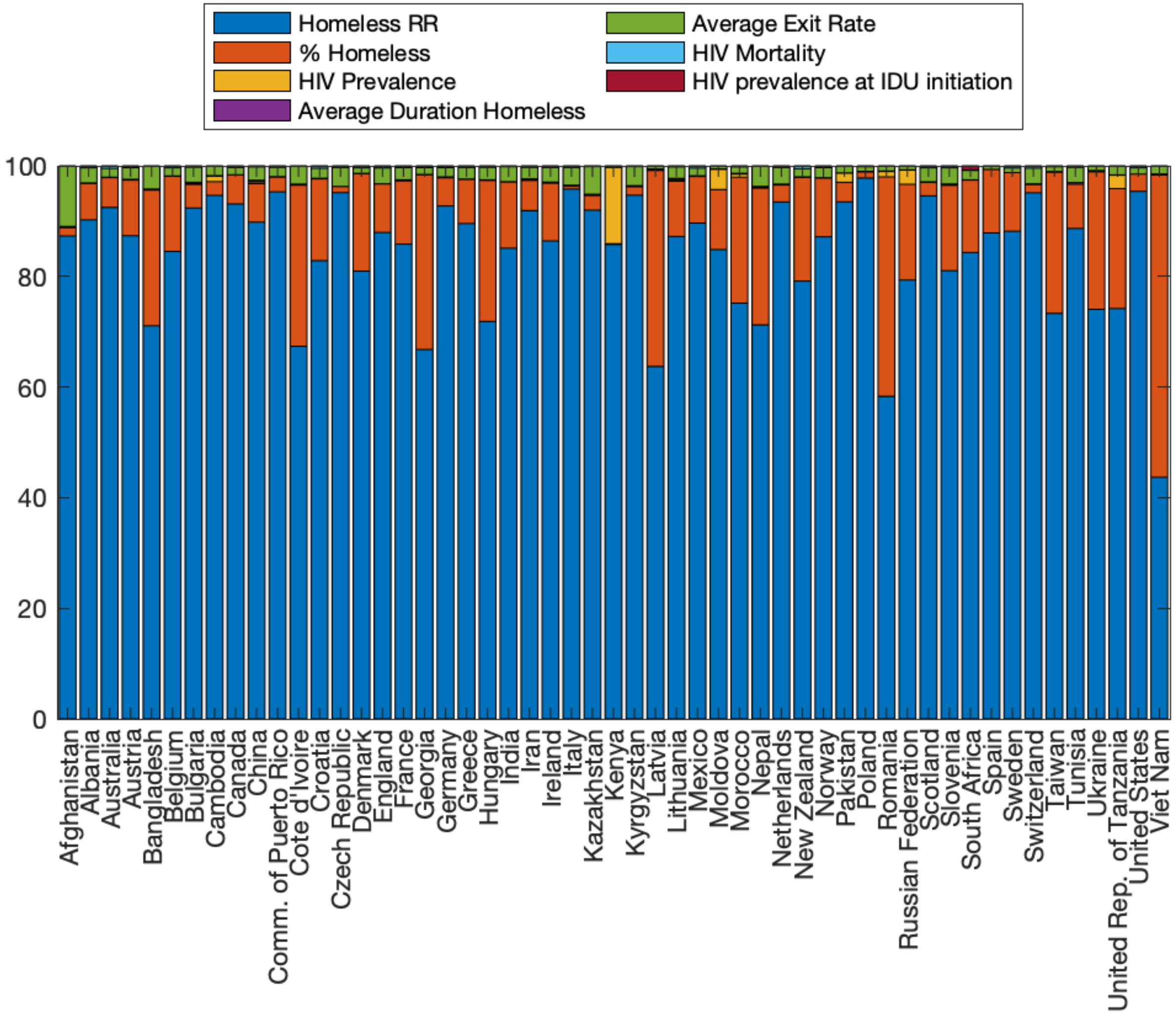

Appendix Figure 4: Results of the uncertainty analyses for the PAF of Unstable Housing on HCV transmission. Stacked bars show how uncertainty in each parameter contributed to the variability in the estimates of the PAF for each country for which the PAF could be estimated without using imputed data.

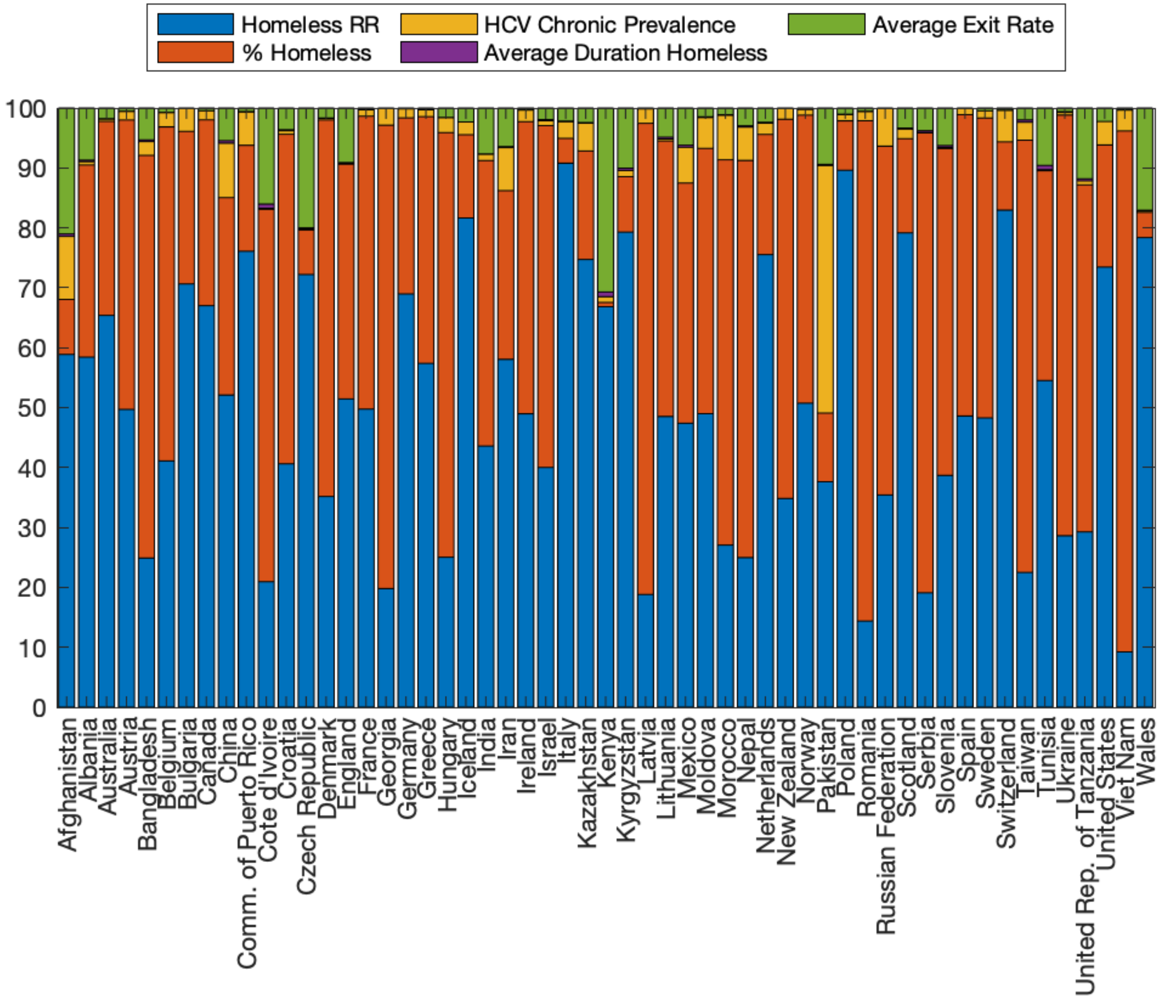

Appendix Table 3: Sensitivity analysis for the Regional & Global tPAFs of Unstable Housing on HIV. *Estimates are the weighted average over all countries within a region with a PWID population size.*

	**Main Analyses**	**Assortative Mixing**	**Unadjusted RR**	**Decreasing incidence**	**Increasing incidence**	**All PWID start injecting stably housed**	**Unstably housed PWID have higher mortality rates**
Australasia	8.8% (2.4-17.7)	24.3% (7.7-38.9)	12.6% (6.3-20.5)	8.6% (2.1-17.1)	9.3% (2.5-18.3)	9.3% (2.6-18.3)	8.8% (2.4-17.7)
Caribbean	11.9% (3.3-22.4)	20.1% (6.0-33.4)	16.5% (8.6-25.7)	11.4% (3.1-21.7)	12.3% (3.3-23.3)	12.5% (3.4-24.1)	12.1% (3.2-22.6)
Central Asia	10.3% (2.8-19.8)	16.3% (4.8-28.2)	14.4% (7.4-23.4)	10.0% (2.6-18.8)	10.5% (2.8-20.7)	11.3% (3.0-22.1)	10.3% (2.8-19.8)
East and South East Asia	6.0% (1.7-12.5)	10.1% (2.9-19.6)	8.6% (4.5-14.8)	5.9% (1.4-12.3)	6.1% (1.6-12.7)	6.7% (1.8-13.7)	6.0% (1.7-12.4)
Eastern Europe	2.2% (0.5-4.8)	2.6% (0.7-6.0)	3.2% (1.5-5.9)	2.3% (0.5-5.2)	2.1% (0.3-4.7)	2.3% (0.6-5.1)	2.2% (0.6-4.8)
Latin America	10.2% (2.7-19.8)	21.3% (6.7-34.6)	14.3% (7.0-23.1)	9.9% (2.5-19.3)	10.7% (3.0-20.6)	10.7% (2.8-20.6)	10.3% (2.7-19.9)
Middle East & North Africa	5.8% (1.6-13.1)	12.2% (3.5-25.7)	8.4% (3.8-15.8)	5.7% (1.5-12.9)	6.0% (1.4-13.7)	6.3% (1.6-14.1)	5.8% (1.5-13.1)
North America	21.6% (6.7-36.3)	23.4% (7.8-37.6)	28.7% (16.4-40.6)	20.8% (6.5-35.4)	22.2% (7.2-37.3)	22.4% (7.1-37.2)	22.1% (6.9-37.3)
Pacific Island States & Territories	-	-	-	-	-	-	-
South Asia	14.1% (4.3-25.6)	13.8% (4.2-24.3)	19.4% (10.5-29.5)	14.1% (4.0-25.8)	13.9% (4.1-25.7)	15.1% (4.5-27.5)	14.3% (4.3-25.8)
Sub Saharan Africa	13.0% (3.3-26.1)	12.8% (3.4-25.0)	17.9% (8.5-30.4)	13.2% (3.4-26.6)	12.7% (3.3-26.1)	13.8% (3.5-28.1)	13/0% (3.3-26.6)
Western Europe	8.2% (2.1-15.9)	15.7% (4.5-27.5)	11.5% (5.9-18.4)	8.0% (2.0-15.6)	8.5% (2.2-16.4)	8.7% (2.3-17.0)	8.2% (2.2 – 16.0)
**Global**	7.9% (2.3-15.7)	9.3% (2.7-17.2)	11.0% (5.8-18.0)	7.9% (2.2-15.5)	8.0% (2.2-15.7)	8.5% (2.4-16.7)	8.1% (2.3 – 16.0)
**High-income**	17.2% (5.1-30.0)	20.5% (6.3-33.9)	23.0% (13.0-34.4)	16.6% (4.9-29.4)	17.6% (5.5-30.8)	17.6% (5.3-31.6)	17.8% (5.4-31.3)
**Low/middle income**	6.6% (1.8-13.1)	7.7% (2.2-14.9)	9.2% (4.7-15.4)	6.6% (1.8-13.2)	6.5% (1.8-13.1)	7.1% (2.0-14.2)	6.7% (1.9-13.3)

Appendix Table 4: Sensitivity analysis for the Regional & Global tPAFs of Unstable Housing on HCV. *Estimates are the weighted average over all countries within a region with a PWID population size.*

	**Main Analyses**	**Assortative Mixing**	**Higher RR in Europe**	**Decreasing incidence**	**Increasing incidence**	**All PWID start injecting stably housed**	**Treat 10% infected PWID per year**	**Unstably housed PWID have higher mortality rates**
Australasia	10.1% (6.9-13.9)	10.2% (7.0-14.0)	10.1% (6.9-13.9)	10.2% (7.0-14.0)	10.0% (6.8-13.6)	10.2% (7.0-14.0)	10.8% (7.4-14.8)	10.1% (6.9-13.9)
Caribbean	10.7% (7.5-14.6)	9.6% (6.7-13.2)	10.7% (7.5-14.6)	11.2% (7.9-15.1)	10.1% (7.2-14.0)	10.4% (7.3-14.3)	12.7% (8.7-17.0)	10.7% (7.5-14.6)
Central Asia	10.1% (6.9-13.6)	9.5% (6.6-12.8)	10.1% (6.9-13.6)	10.6% (7.3-14.3)	9.5% (6.5-12.8)	10.4% (7.2-14.0)	10.9% (7.5-14.7)	10.1% (6.9-13.6)
East and South East Asia	7.4% (4.9-11.2)	8.0% (5.3-12.4)	7.4% (4.9-11.2)	7.6% (5.1-11.3)	7.2% (4.6-11.1)	7.8% (5.1-12.0)	7.9% (5.2-11.7)	7.4% (4.9-11.2)
Eastern Europe	2.8% (1.6-4.2)	2.5% (1.5-3.9)	4.3% (2.5-7.1)	3.0% (1.7-4.6)	2.6% (1.5-4.0)	2.8% (1.6-4.2)	3.2% (1.7-5.1)	2.8% (1.7-4.3)
Latin America	7.8% (5.0-11.5)	6.8% (4.3-10.1)	7.8% (5.0-11.5)	8.3% (5.4-12.0)	7.3% (4.6-10.9)	7.3% (4.5-11.0)	10.3% (6.3-14.6)	7.8% (4.9-11.4)
Middle East & North Africa	6.9% (3.7-11.1)	7.4% (4.0-12.1)	6.9% (3.7-11.1)	7.1% (3.7-11.5)	6.8% (3.6-11.1)	7.2% (3.8-11.8)	7.6% (3.9-12.0)	6.9% (3.7-11.3)
North America	26.2% (19.5-33.2)	25.1% (18.8-31.5)	26.2% (19.5-33.2)	26.3% (19.5-33.3)	26.0% (19.5-33.1)	26.4% (19.7-33.5)	27.6% (20.7-34.8)	26.5% (19.8-33.6)
Pacific Island States & Territories	-	-	-	-	-	-	-	
South Asia	21.4% (15.0-29.2)	20.6% (14.2-27.7)	21.4% (15.0-29.2)	21.7% (15.3-29.5)	20.9% (14.5-28.6)	22.8% (15.7-31.1)	22.2% (15.7-29.9)	21.7% (15.1-29.4)
Sub Saharan Africa	21.9% (12.5-33.8)	23.4% (15.0-32.6)	21.9% (12.5-33.8)	21.5% (12.4-33.2)	21.8% (12.5-33.6)	23.6% (13.5-36.3)	22.3% (12.8-34.2)	22.0% (12.5-34.1)
Western Europe	10.6% (7.3-14.4)	10.5% (7.3-14.2)	15.9% (11.0-22.0)	10.8% (7.5-14.6)	10.5% (7.2-14.2)	10.8% (7.4-14.6)	11.3% (7.8-15.2)	10.7% (7.4-14.4)
**Global**	11.2% (7.7-15.5)	11.1% (7.6-15.1)	12.3% (9.1-16.1)	11.3% (7.9-15.5)	11.1% (7.5-15.4)	11.6% (8.0-15.9)	11.8% (8.1-16.1)	11.6% (8.0–16.0)
**High-income**	19.4% (13.8-26.0)	18.6% (13.3-24.9)	21.4% (16.7-27.1)	19.5% (13.8-26.2)	19.3% (13.7-26.0)	19.5% (13.9-26.4)	20.4% (14.4-27.2)	20.0% (14.2-26.6)
**Low/middle income**	8.3% (5.5-11.7)	8.3% (5.5-11.7)	8.8% (6.4-12.0)	8.4% (5.6-11.7)	8.2% (5.4-11.6)	8.8% (5.8-12.4)	8.7% (5.7-12.2)	8.4% (5.6-11.8)

Appendix Table 5: Regional & Global tPAFs of Unstable Housing on HIV and HCV including countries with imputed data. *Estimates are the weighted average over all countries within a region with a PWID population size.*

	HIV tPAF	HCV tPAF
Australasia	8.8% (2.4-17.7)	10.1% (6.9-13.9)
Caribbean	11.9% (3.3-22.4)	10.7% (7.5-14.6)
Central Asia	9.8% (2.6-18.8)	10.2% (7.2-13.8)
East and South East Asia	5.6% (1.5-11.3)	6.8% (4.7-9.7)
Eastern Europe	2.2% (0.6-4.8)	2.9% (1.7-4.3)
Latin America	6.6% (1.7-14.4)	10.8% (7.5-14.8)
Middle East & North Africa	5.5% (1.4-10.8)	6.8% (4.7-9.3)
North America	21.6% (6.7-36.3)	26.2% (19.5-33.2)
Pacific Island States & Territories	-	-
South Asia	14.1% (4.3-25.6)	21.4% (15-29.2)
Sub Saharan Africa	13% (3.3-25.5)	23% (15.6-32.4)
Western Europe	8.3% (2.2-16.1)	10.6% (7.4-14.3)
**Global**	7.5% (2.1-14.5)	10.7% (7.6-14.3)
**High-income**	14.1% (4.1-25.4)	16.8% (11.9-22.8)
**Low/middle income**	6.5% (1.8-12.9)	8.6% (6.0-11.6)

Appendix Table 6: Sensitivity analysis for the country-level tPAFs of Unstable Housing on HIV. *Countries marked with an asterisk denotes that the country had 'insufficient data' and so regional data for were used.*

	Main Analyses	Assortative Mixing	Unadjusted RR	Decreasing incidence	Increasing incidence	All PWID start injecting stably housed	Unstably housed PWID have higher mortality rates
**Australasia**							
Australia	9.0% (2.4-17.9)	24.2% (7.7-38.9)	12.7% (6.3-20.7)	8.7% (2.1-17.2)	9.4% (2.5-18.5)	9.4% (2.6-18.5)	9.0% (2.4-17.9)
New Zealand	6.8% (1.9-14.4)	25.8% (8.6-40.7)	9.7% (4.8-17.3)	6.7% (1.7-14.1)	7.2% (2.0-15.0)	7.1% (2.0-15.6)	6.7% (1.8-14.7)
**Caribbean**							
Antigua & Barbuda*	11.3% (3.2-21.4)	15.7% (4.6-27.9)	15.7% (8.2-24.6)	11.0% (3.1-21.1)	11.6% (3.3-22.2)	11.8% (3.3-22.4)	11.4% (3.2-21.7)
Bahamas*	11.2% (3.1-21.2)	15.8% (4.7-27.4)	15.6% (8.4-24.3)	11.1% (3.0-20.5)	11.5% (3.2-21.8)	11.8% (3.4-22.7)	11.4% (3.2-21.4)
Barbados*	11.3% (3.2-21.4)	15.5% (4.7-27.6)	15.7% (8.4-24.8)	11.0% (2.9-21.0)	11.5% (3.3-21.9)	11.8% (3.4-22.8)	11.3% (3.2-21.8)
Bermuda*	11.0% (3.2-21.3)	15.5% (4.7-27.4)	15.4% (8.5-24.5)	10.7% (3.0-20.9)	11.4% (3.4-21.9)	11.6% (3.4-22.7)	11.1% (3.2-21.6)
Comm. of Puerto Rico	11.9% (3.3-22.4)	20.1% (6.0-33.4)	16.5% (8.6-25.7)	11.4% (3.1-21.7)	12.3% (3.3-23.3)	12.5% (3.4-24.1)	12.1% (3.2-22.6)
Cuba*	11.1% (3.1-21.1)	15.5% (4.5-27.6)	15.3% (8.0-24.2)	10.7% (2.6-21.0)	11.4% (3.3-21.7)	11.6% (3.2-22.3)	11.3% (3.1-21.4)
Dominica*	11.2% (3.1-21.3)	15.6% (4.5-28.4)	15.5% (8.0-24.5)	10.9% (2.9-21.0)	11.5% (3.3-21.8)	11.8% (3.3-22.5)	11.3% (3.2-21.6)
Dominican Republic*	11.4% (3.1-20.8)	15.8% (4.6-27.4)	15.9% (8.0-24.2)	11.1% (3.0-20.7)	11.8% (3.2-21.5)	12.0% (3.3-22.3)	11.5% (3.2-21.1)
Grenada*	11.3% (3.1-21.0)	15.6% (4.5-27.4)	15.5% (8.2-24.2)	11.1% (2.9-20.6)	11.5% (3.4-21.6)	11.8% (3.3-22.3)	11.3% (3.1-21.1)
Haiti*	11.2% (3.0-20.7)	15.6% (4.5-27.0)	15.5% (8.1-23.9)	11.0% (2.8-20.3)	11.4% (3.2-21.4)	11.7% (3.2-22.0)	11.3% (3.0-21.0)
Jamaica*	11.4% (3.1-20.8)	15.6% (4.5-27.5)	15.8% (8.4-24.3)	11.1% (2.7-20.6)	11.6% (3.2-21.5)	11.9% (3.2-22.4)	11.4% (3.1-21.2)
Saint Kitts & Nevis*	11.2% (3.1-20.8)	15.5% (4.6-28.0)	15.6% (8.2-24.4)	11.0% (3.1-20.6)	11.6% (3.2-21.7)	11.9% (3.3-22.2)	11.5% (3.2-21.1)
Saint Lucia*	11.2% (3.3-21.6)	15.5% (4.6-27.8)	15.6% (8.5-24.4)	10.9% (3.2-20.7)	11.5% (3.3-22.0)	11.9% (3.5-22.9)	11.3% (3.4-21.7)
St Vincent & the Grenadines*	11.2% (3.3-21.2)	15.6% (4.5-27.6)	15.4% (8.4-24.3)	11.0% (2.9-20.6)	11.5% (3.4-21.6)	11.8% (3.5-22.5)	11.3% (3.2-21.4)
Trinidad & Tobago*	10.9% (3.1-21.0)	15.4% (4.6-27.9)	15.4% (8.1-23.9)	10.8% (3.0-20.4)	11.3% (3.2-21.8)	11.6% (3.3-22.3)	11.1% (3.1-21.4)
**Central Asia**							
Kazakhstan	9.8% (2.6-19.8)	16.3% (4.8-28.6)	13.8% (6.9-23.1)	9.6% (2.4-18.7)	10.0% (2.6-20.7)	10.9% (2.8-22.1)	9.8% (2.5-19.8)
Kyrgyzstan	11.4% (3.2-21.3)	15.9% (4.8-27.7)	15.7% (8.4-24.9)	11.0% (2.8-20.6)	11.6% (3.2-21.9)	12.3% (3.5-23.5)	11.3% (3.2-21.4)
Tajikistan*	7.7% (2.1-15.7)	8.5% (2.4-16.5)	11.0% (5.7-18.1)	7.9% (2.2-15.9)	7.5% (2.0-15.0)	8.3% (2.3-16.9)	7.8% (2.1-15.8)
Turkmenistan*	10.0% (2.6-19.8)	15.5% (4.6-27.2)	14.0% (7.1-23.0)	9.7% (2.3-19.4)	10.1% (2.7-20.3)	11.1% (3.0-21.9)	10.0% (2.7-19.8)
Uzbekistan*	10.1% (2.6-20.0)	17.9% (5.5-30.7)	14.2% (7.4-23.1)	9.8% (2.4-19.0)	10.4% (2.7-20.8)	11.1% (3.0-21.8)	10.1% (2.6-20.0)
**East and South East Asia**							
Brunei Darussalam*	5.6% (1.4-12.3)	8.8% (2.5-18.0)	8.0% (4.0-14.7)	5.6% (1.3-12.0)	5.8% (1.5-12.8)	6.1% (1.6-13.4)	5.6% (1.5-12.4)
Cambodia	9.1% (2.5-17.8)	10.4% (2.9-19.7)	12.8% (6.7-20.8)	9.0% (2.8-17.7)	9.1% (2.4-17.6)	9.7% (2.7-19.1)	9.2% (2.5-17.7)
China	5.9% (1.6-12.3)	10.2% (2.9-20.1)	8.5% (4.3-14.7)	5.8% (1.3-11.9)	6.0% (1.5-12.7)	6.5% (1.8-13.8)	5.9% (1.7-12.2)
Hong Kong (China)*	4.9% (1.2-10.0)	8.1% (2.1-16.1)	7.1% (3.4-12.0)	4.8% (1.1-10.0)	5.1% (1.1-10.3)	5.2% (1.3-10.7)	4.9% (1.2-10.1)
Indonesia*	3.5% (0.9-7.4)	3.3% (0.9-6.9)	5.0% (2.4-8.9)	3.8% (1.0-8.2)	3.2% (0.8-7.0)	3.6% (0.9-7.6)	3.5% (0.9-7.4)
Japan*	5.8% (1.5-11.7)	9.0% (2.4-17.7)	8.3% (3.9-14.1)	5.7% (1.2-11.5)	5.8% (1.3-12.1)	6.3% (1.6-13.1)	5.8% (1.5-11.7)
Lao PDR*	5.4% (1.4-11.3)	7.7% (2.1-16.8)	7.7% (3.8-13.7)	5.4% (1.3-11.3)	5.4% (1.3-11.6)	5.8% (1.5-12.5)	5.3% (1.4-11.3)
Malaysia*	5.0% (1.3-10.5)	7.5% (2.0-14.6)	7.1% (3.5-12.3)	5.1% (1.2-10.3)	5.1% (1.1-10.7)	5.3% (1.3-11.2)	5.0% (1.3-10.3)
Mongolia*	5.8% (1.5-12.4)	9.0% (2.4-18.2)	8.3% (4.0-14.8)	5.7% (1.3-12.0)	5.8% (1.2-12.5)	6.2% (1.6-13.6)	5.7% (1.5-12.3)
Myanmar*	6.0% (1.6-13.2)	7.1% (1.9-14.8)	8.7% (4.4-16.2)	6.3% (1.6-13.9)	5.7% (1.3-12.9)	6.7% (1.8-15.1)	6.0% (1.6-13.0)
North Korea*	5.6% (1.4-12.3)	8.7% (2.3-18.4)	8.1% (3.8-14.7)	5.6% (1.3-12.4)	5.7% (1.2-12.6)	6.1% (1.6-13.5)	5.6% (1.4-12.5)
Philippines*	5.4% (1.5-11.6)	7.3% (2.1-15.3)	7.9% (3.9-14.1)	5.6% (1.2-11.8)	5.4% (1.3-11.8)	5.9% (1.6-13.1)	5.4% (1.4-11.5)
Republic of Korea	NA	NA	NA	NA	NA	NA	NA
Singapore*	5.6% (1.5-11.9)	8.8% (2.5-17.2)	8.1% (3.9-14.0)	5.7% (1.5-11.9)	5.7% (1.2-12.1)	6.1% (1.7-12.9)	5.6% (1.5-11.8)
Taiwan	0.4% (0.1-1.1)	1.0% (0.2-2.4)	0.6% (0.3-1.4)	0.5% (-0.9-2.1)	0.4% (-1.0-2.0)	0.5% (0.1-1.2)	0.4% (0.1-1.1)
Thailand*	5.0% (1.2-10.5)	6.1% (1.5-12.7)	7.1% (3.5-12.5)	5.1% (1.2-10.7)	5.0% (1.2-10.3)	5.4% (1.4-11.4)	5.0% (1.3-10.5)
Timor Leste*	5.6% (1.4-12.4)	8.8% (2.4-18.0)	8.2% (4.0-14.7)	5.7% (1.4-12.2)	5.8% (1.4-12.7)	6.1% (1.6-13.4)	5.6% (1.4-12.4)

	Main Analyses	Assortative Mixing	Unadjusted RR	Decreasing incidence	Increasing incidence	All PWID start injecting stably housed	Unstably housed PWID have higher mortality rates
Viet Nam	6.5% (0.9-18.1)	9.2% (1.6-22.2)	9.4% (2.0-22.3)	6.5% (0.9-17.9)	6.5% (0.5-18.2)	7.2% (1.0-20.1)	6.4% (0.9-18.2)
**Eastern Europe**							
Armenia*	4.4% (1.1-9.3)	13.2% (3.7-24.3)	6.4% (3.0-11.3)	4.4% (0.9-9.3)	4.7% (0.9-10.1)	4.8% (1.2-10.5)	4.4% (1.1-9.4)
Azerbaijan*	4.2% (1.1-9.1)	9.3% (2.4-18.5)	6.1% (2.9-10.9)	4.2% (0.6-9.3)	4.4% (0.8-9.7)	4.5% (1.1-9.9)	4.2% (1.1-9.0)
Belarus*	3.4% (0.9-7.1)	4.3% (1.2-8.9)	5.0% (2.4-8.7)	3.5% (0.9-7.3)	3.4% (0.8-7.3)	3.6% (0.9-7.7)	3.4% (0.8-7.2)
Bosnia & Herzegovina*	4.1% (1.0-8.9)	25.2% (8.2-39.5)	6.0% (2.9-10.4)	4.2% (0.6-8.6)	4.3% (0.8-9.4)	4.4% (1.1-9.6)	4.1% (1.1-8.8)
Bulgaria	2.1% (0.5-4.6)	6.3% (1.7-13.9)	3.1% (1.4-5.5)	2.2% (-0.1-5.1)	2.2% (-0.1-5.2)	2.3% (0.6-5.2)	2.1% (0.5-4.6)
Czech Republic	25.8% (8.0-42.1)	26.7% (8.9-41.7)	33.5% (19.8-46.6)	24.4% (7.4-40.1)	27.1% (8.5-44.1)	26.8% (8.3-43.7)	26.4% (8.4-42.7)
Estonia*	2.1% (0.6-4.5)	1.9% (0.5-4.1)	3.0% (1.4-5.4)	2.4% (0.6-4.9)	1.9% (0.4-4.0)	2.0% (0.6-4.5)	2.1% (0.6-4.5)
Georgia	1.9% (0.5-5.1)	12.3% (3.6-23.9)	2.8% (1.1-6.2)	2.1% (-0.2-5.2)	2.0% (-0.4-5.4)	2.0% (0.5-5.5)	1.9% (0.5-5.0)
Hungary	12.0% (3.1-25.3)	26.4% (8.8-41.4)	16.7% (7.0-29.3)	11.2% (3.0-24.0)	12.6% (3.0-26.3)	12.7% (3.4-26.5)	12.1% (3.1-25.4)
Latvia	0.7% (0.2-2.0)	0.9% (0.2-2.4)	1.1% (0.4-2.4)	0.9% (-0.3-2.3)	0.7% (-0.5-2.1)	0.8% (0.2-2.0)	0.7% (0.2-1.9)
Lithuania	3.8% (1.0-8.5)	9.7% (2.6-21.7)	5.6% (2.7-10.1)	3.9% (0.6-8.6)	4.0% (0.8-9.0)	4.2% (1.1-9.1)	3.8% (1.0-8.4)
Moldova	3.4% (0.9-7.8)	4.1% (1.1-10.3)	4.9% (2.3-9.4)	3.5% (0.7-8.0)	3.4% (0.8-7.9)	3.6% (1.0-8.2)	3.4% (0.9-7.8)
Poland	9.4% (2.6-17.7)	12.3% (3.6-22.0)	13.1% (6.7-20.5)	9.2% (2.5-17.5)	9.4% (2.6-17.9)	9.8% (2.7-18.6)	9.4% (2.6-17.7)
Romania	8.0% (1.4-20.5)	10.3% (2.0-24.5)	11.8% (3.4-24.0)	8.1% (1.6-20.0)	8.0% (1.3-20.7)	8.6% (1.5-21.7)	8.1% (1.4-20.4)
Russian Federation	2.2% (0.5-5.0)	2.5% (0.6-5.9)	3.2% (1.4-6.1)	2.4% (0.5-5.3)	2.1% (0.3-5.1)	2.3% (0.6-5.5)	2.2% (0.5-5.1)
Slovakia*	4.7% (1.1-10.4)	26.6% (8.8-41.7)	6.6% (3.2-12.1)	4.6% (0.6-10.0)	4.9% (1.0-11.2)	5.1% (1.2-11.3)	4.7% (1.2-10.4)
Ukraine	0.3% (0.1-0.6)	0.4% (0.1-1.0)	0.4% (0.2-0.8)	0.4% (-0.9-2.0)	0.2% (-1.1-1.3)	0.3% (0.1-0.7)	0.3% (0.1-0.6)
**Latin America**							
Argentina*	6.4% (1.9-13.4)	5.9% (1.7-12.4)	9.1% (4.6-15.7)	6.7% (1.9-13.7)	6.1% (1.7-12.8)	6.4% (1.8-13.4)	6.5% (1.9-13.3)
Belize*	7.8% (2.0-16.1)	8.1% (2.2-17.1)	11.0% (5.5-18.8)	8.0% (2.0-16.5)	7.8% (2.0-15.7)	8.0% (2.1-16.4)	7.8% (2.1-16.0)
Bolivia*	7.6% (2.1-16.0)	8.0% (2.2-17.8)	10.9% (5.5-18.8)	7.8% (2.1-16.0)	7.5% (2.0-16.2)	7.8% (2.2-16.7)	7.6% (2.2-16.3)
Brazil*	6.5% (1.6-14.6)	6.1% (1.5-15.1)	9.3% (3.7-17.1)	6.8% (1.7-15.0)	6.1% (1.5-14.3)	6.4% (1.6-14.8)	6.5% (1.7-14.6)
Chile*	7.5% (2.1-15.5)	8.1% (2.1-17.1)	10.9% (5.3-18.4)	7.7% (1.9-15.7)	7.5% (2.0-15.5)	7.7% (2.2-16.0)	7.6% (2.1-15.8)
Colombia*	16.8% (4.9-33.5)	23.7% (7.8-38.7)	23.4% (12.5-38.3)	15.7% (4.4-31.0)	17.7% (4.9-35.7)	19.2% (5.5-37.6)	16.8% (4.9-33.8)
Costa Rica*	7.9% (2.0-15.8)	8.3% (2.2-17.9)	11.1% (5.4-18.9)	8.2% (2.1-16.0)	7.8% (2.1-16.1)	8.1% (2.1-16.7)	7.9% (2.0-16.0)
Ecuador*	7.7% (2.0-16.1)	8.1% (2.2-17.5)	10.9% (5.2-18.9)	7.9% (2.0-16.0)	7.6% (2.0-16.1)	7.8% (2.1-16.5)	7.7% (2.1-16.2)
El Salvador*	7.8% (2.0-15.7)	8.2% (2.2-17.4)	11.0% (5.5-18.7)	8.0% (2.1-15.8)	7.7% (2.1-15.6)	7.9% (2.1-16.3)	7.8% (2.0-15.9)
Guatemala*	7.8% (2.0-16.2)	8.0% (2.0-17.5)	11.0% (5.2-18.9)	8.0% (1.9-16.1)	7.7% (1.9-16.2)	7.9% (2.0-16.8)	7.8% (1.9-16.4)
Guyana*	7.7% (2.2-16.1)	8.1% (2.2-17.9)	10.9% (5.6-19.0)	7.9% (2.2-16.2)	7.5% (2.1-16.2)	7.8% (2.3-16.7)	7.7% (2.2-16.1)
Honduras*	7.8% (2.2-15.4)	8.1% (2.2-16.7)	11.0% (5.6-18.6)	8.0% (2.3-15.7)	7.6% (2.2-15.3)	7.9% (2.3-16.0)	7.8% (2.2-15.4)
Mexico	10.2% (2.7-19.8)	21.3% (6.7-34.6)	14.3% (7.0-23.1)	9.9% (2.5-19.3)	10.7% (3.0-20.6)	10.7% (2.8-20.6)	10.3% (2.7-19.9)
Nicaragua*	12.7% (3.4-25.2)	21.3% (6.6-36.3)	17.8% (9.3-29.4)	12.3% (3.2-23.8)	13.3% (3.7-26.9)	13.8% (3.8-27.7)	12.8% (3.6-25.3)
Panama*	7.7% (2.0-15.7)	8.0% (2.0-17.3)	10.9% (5.4-18.7)	7.9% (2.1-15.8)	7.6% (2.0-15.5)	7.9% (2.0-16.4)	7.8% (2.0-15.9)
Paraguay*	9.8% (2.7-18.6)	16.5% (4.9-29.1)	13.8% (6.9-21.6)	9.6% (2.5-18.1)	10.2% (2.7-19.3)	10.3% (2.8-19.8)	9.8% (2.7-18.8)
Peru*	9.5% (2.6-18.4)	14.3% (4.1-25.7)	13.1% (6.6-21.6)	9.2% (2.6-18.1)	9.7% (2.8-18.9)	9.8% (2.7-19.4)	9.5% (2.5-18.5)
Suriname*	7.7% (2.2-15.6)	8.0% (2.3-17.1)	11.0% (5.6-18.4)	7.8% (2.3-15.8)	7.5% (2.2-15.5)	7.8% (2.2-15.9)	7.7% (2.2-15.7)
Uruguay*	9.2% (2.5-17.8)	12.1% (3.5-22.2)	12.8% (6.6-20.7)	9.1% (2.3-17.4)	9.3% (2.5-18.2)	9.5% (2.6-18.3)	9.2% (2.6-17.9)
Venezuela*	7.8% (2.2-15.9)	8.0% (2.3-17.0)	11.1% (5.6-18.8)	8.0% (2.2-16.0)	7.5% (2.1-15.9)	7.9% (2.2-16.2)	7.8% (2.2-16.0)
**Middle East & North Africa**							
Algeria*	6.0% (1.5-13.3)	20.3% (6.1-36.8)	8.6% (3.9-15.9)	5.8% (1.2-13.1)	6.2% (1.4-13.8)	6.5% (1.6-14.6)	6.0% (1.5-13.2)
Bahrain*	5.9% (1.6-13.5)	15.4% (4.7-29.3)	8.5% (3.9-16.1)	5.6% (1.2-13.1)	6.0% (1.4-14.2)	6.3% (1.7-14.5)	5.8% (1.6-13.5)
Cyprus*	6.2% (1.7-14.5)	23.2% (7.6-38.0)	9.1% (4.1-17.3)	6.1% (1.3-13.8)	6.5% (1.5-15.5)	6.8% (1.8-16.3)	6.2% (1.6-14.6)
Egypt*	6.0% (1.5-14.0)	19.7% (6.0-34.0)	8.9% (4.0-16.8)	6.0% (1.2-13.5)	6.5% (1.5-14.7)	6.7% (1.7-15.3)	6.1% (1.5-14.0)
Iraq*	6.0% (1.5-13.8)	17.4% (5.4-31.8)	8.7% (3.9-16.5)	5.9% (1.5-13.2)	6.3% (1.3-14.3)	6.5% (1.6-15.0)	6.0% (1.5-13.7)

	Main Analyses	Assortative Mixing	Unadjusted RR	Decreasing incidence	Increasing incidence	All PWID start injecting stably housed	Unstably housed PWID have higher mortality rates
Israel	NA	NA	NA	NA	NA	NA	NA
Jordan*	6.0% (1.5-13.5)	17.5% (5.1-32.1)	8.8% (3.8-16.1)	6.0% (1.3-13.2)	6.3% (1.3-14.3)	6.5% (1.7-14.8)	6.0% (1.5-13.8)
Kuwait*	6.0% (1.6-13.5)	17.4% (5.4-31.9)	8.6% (3.9-16.1)	5.8% (1.5-13.5)	6.3% (1.3-14.4)	6.4% (1.7-15.1)	6.0% (1.6-13.6)
Lebanon	NA	NA	NA	NA	NA	NA	NA
Libyan Arab Jamahiriya*	0.4% (0.1-1.0)	0.4% (0.1-0.9)	0.6% (0.3-1.2)	0.5% (0.1-1.1)	0.4% (0.1-0.9)	0.2% (0.1-0.6)	0.4% (0.1-1.0)
Morocco	6.3% (1.7-14.4)	12.1% (3.3-27.6)	9.1% (3.9-17.2)	6.2% (1.5-14.2)	6.4% (1.5-14.8)	6.7% (1.7-15.5)	6.3% (1.6-14.5)
Occ. Palestinian Territories	NA	NA	NA	NA	NA	NA	NA
Oman*	5.4% (1.4-12.2)	9.8% (2.7-21.4)	7.9% (3.7-14.7)	5.4% (1.3-12.3)	5.7% (1.3-12.8)	5.9% (1.6-13.2)	5.5% (1.4-12.3)
Qatar*	6.0% (1.4-14.1)	17.4% (5.1-32.0)	8.7% (3.6-16.8)	5.8% (1.3-13.3)	6.2% (1.1-14.7)	6.5% (1.6-15.2)	5.9% (1.4-14.2)
Saudi Arabia*	5.8% (1.5-12.9)	11.2% (3.2-22.3)	8.3% (3.8-15.1)	5.7% (1.3-12.5)	5.9% (1.4-13.2)	6.1% (1.6-13.7)	5.8% (1.5-12.8)
South Sudan*	6.1% (1.5-14.1)	17.7% (5.1-32.1)	8.7% (3.8-17.0)	5.8% (1.2-13.7)	6.2% (1.4-14.9)	6.5% (1.5-15.4)	6.0% (1.4-14.0)
Sudan	NA	NA	NA	NA	NA	NA	NA
Syrian Arab Rep.	NA	NA	NA	NA	NA	NA	NA
Tunisia	3.4% (0.9-7.3)	13.8% (4.1-25.3)	4.9% (2.3-8.8)	3.3% (0.3-7.3)	3.5% (0.4-7.9)	3.7% (0.9-8.2)	3.4% (0.9-7.3)
Turkey*	8.2% (2.0-18.9)	26.3% (8.7-41.1)	11.8% (5.2-22.6)	7.7% (1.6-17.5)	9.0% (1.8-20.6)	9.4% (2.4-22.0)	8.2% (2.0-18.9)
United Arab Emirates*	5.9% (1.5-14.0)	17.6% (5.1-31.7)	8.6% (4.0-16.8)	5.9% (1.3-13.8)	6.1% (1.3-14.6)	6.4% (1.7-15.3)	5.9% (1.5-14.2)
Yemen*	6.0% (1.5-14.1)	17.6% (5.2-32.0)	8.7% (4.0-17.1)	5.8% (1.1-13.4)	6.3% (1.3-14.8)	6.5% (1.6-15.6)	6.0% (1.5-14.3)
**North America**							
Canada	18.7% (5.7-33.0)	21.6% (7.0-35.7)	25.2% (14.1-37.0)	18.1% (5.5-32.5)	19.1% (6.0-33.7)	19.2% (6.0-34.2)	18.9% (5.8-33.6)
United States	22.0% (6.9-37.2)	23.8% (7.9-38.0)	29.2% (16.8-41.5)	21.1% (6.5-36.0)	22.7% (7.4-38.1)	22.7% (7.3-38.2)	22.6% (7.1-38.3)
**South Asia**							
Afghanistan	24.7% (7.2-45.0)	25.6% (8.3-40.6)	33.3% (18.3-50.9)	23.0% (6.6-41.9)	26.6% (7.6-48.5)	28.3% (8.3-50.5)	25.1% (7.3-45.2)
Bangladesh	10.2% (2.4-23.4)	25.9% (8.7-41.1)	14.6% (5.8-27.2)	9.9% (2.0-21.4)	10.8% (2.2-25.0)	11.4% (2.8-25.7)	10.2% (2.4-23.2)
Bhutan*	15.6% (4.5-29.1)	16.7% (5.1-29.2)	21.2% (11.4-33.7)	15.4% (4.4-29.0)	15.6% (4.6-29.3)	16.7% (4.9-31.5)	15.7% (4.6-29.4)
India	22.4% (6.6-39.8)	21.6% (6.8-35.9)	29.5% (15.6-44.3)	21.9% (6.6-38.9)	22.8% (6.7-40.6)	23.6% (7.2-41.6)	22.9% (6.7-40.3)
Iran	17.7% (5.1-32.8)	19.8% (6.2-33.7)	23.9% (13.2-37.0)	17.3% (5.1-31.8)	17.9% (5.3-33.1)	18.7% (5.5-34.3)	17.8% (5.2-32.8)
Maldives	NA	NA	NA	NA	NA	NA	NA
Nepal	0.9% (0.2-2.3)	2.3% (0.6-5.4)	1.3% (0.5-2.8)	1.1% (-1.1-3.2)	0.9% (-1.3-3.4)	1.0% (0.2-2.7)	0.9% (0.2-2.3)
Pakistan	11.5% (3.2-22.5)	11.2% (3.2-21.0)	16.1% (8.6-26.3)	12.1% (3.3-23.4)	10.9% (3.1-21.5)	12.2% (3.4-23.8)	11.6% (3.2-22.3)
Sri Lanka	NA	NA	NA	NA	NA	NA	NA
**Sub Saharan Africa**							
Angola*	17.4% (4.6-34.7)	23.2% (7.5-38.0)	24.0% (11.5-39.6)	16.5% (4.5-32.8)	18.4% (5.1-36.5)	19.1% (5.1-37.8)	17.5% (4.7-34.7)
Benin*	12.6% (3.5-24.5)	15.0% (4.3-27.3)	17.5% (8.4-29.0)	12.4% (3.7-24.5)	12.8% (3.2-24.9)	13.2% (3.7-25.9)	12.7% (3.5-24.8)
Botswana*	13.1% (3.4-26.1)	15.4% (4.4-28.3)	18.1% (8.7-30.3)	13.0% (3.3-25.8)	13.4% (3.6-26.4)	13.9% (3.7-27.6)	13.1% (3.4-26.2)
Burkina Faso*	15.1% (4.2-32.0)	16.5% (4.8-31.1)	21.2% (10.0-36.6)	15.0% (4.0-31.6)	15.2% (4.5-32.6)	16.5% (4.7-35.0)	15.2% (4.3-32.1)
Burundi*	15.1% (4.0-30.8)	16.5% (4.7-29.8)	21.4% (10.5-35.5)	14.9% (3.7-30.6)	15.2% (4.2-30.5)	16.6% (4.3-33.7)	15.3% (4.1-30.5)
Cameroon*	15.3% (4.2-31.1)	16.5% (4.9-30.7)	21.0% (10.5-36.0)	15.0% (4.3-30.7)	15.2% (4.4-31.6)	16.5% (4.8-34.0)	15.4% (4.2-31.5)
Cape Verde*	15.2% (4.3-31.5)	16.6% (5.1-30.6)	21.0% (10.2-36.1)	14.9% (4.3-31.0)	15.2% (4.2-31.6)	16.4% (4.9-33.9)	15.2% (4.4-31.8)
Central African Republic*	15.0% (4.2-30.7)	16.6% (4.7-29.7)	21.1% (10.0-35.2)	14.9% (4.3-30.0)	15.1% (4.1-30.9)	16.4% (4.5-32.3)	15.1% (4.1-31.1)
Chad*	15.4% (4.2-30.9)	16.5% (4.6-30.8)	21.4% (10.6-35.9)	15.1% (4.1-30.5)	15.4% (4.1-30.8)	16.5% (4.5-33.4)	15.5% (4.2-31.3)
Comoros*	15.6% (4.0-30.6)	16.8% (4.7-30.2)	21.5% (10.4-36.1)	15.7% (4.0-30.7)	15.6% (4.2-30.7)	17.0% (4.5-33.4)	15.7% (4.0-30.6)
Congo (Kinshasa)*	16.7% (5.1-34.6)	18.7% (5.8-33.8)	23.0% (11.2-39.2)	16.1% (4.9-33.3)	17.1% (4.8-35.6)	18.0% (5.7-37.2)	16.7% (5.1-35.0)
Cote d'Ivoire	8.1% (1.9-19.5)	16.4% (4.6-31.9)	11.8% (4.5-23.1)	7.9% (1.8-18.0)	8.5% (1.7-20.3)	9.0% (2.1-21.9)	8.2% (1.9-19.2)
Djibouti*	15.2% (4.5-31.2)	16.6% (5.0-31.0)	21.2% (10.6-36.8)	15.1% (4.5-30.3)	15.4% (4.4-31.6)	16.4% (4.8-33.2)	15.5% (4.4-31.2)
Equatorial Guinea*	14.8% (4.2-30.2)	16.2% (5.0-29.6)	20.7% (10.3-34.8)	14.7% (4.2-29.8)	15.1% (4.4-30.3)	16.1% (4.6-32.5)	14.8% (4.3-30.2)
Eritrea*	15.4% (4.3-32.8)	16.8% (4.9-31.2)	21.4% (10.7-37.4)	15.3% (4.2-31.5)	15.4% (4.2-33.2)	16.7% (4.8-35.4)	15.4% (4.4-33.4)

	Main Analyses	Assortative Mixing	Unadjusted RR	Decreasing incidence	Increasing incidence	All PWID start injecting stably housed	Unstably housed PWID have higher mortality rates
Ethiopia*	15.2% (4.2-31.3)	16.4% (4.8-31.3)	21.1% (10.0-36.4)	14.9% (3.9-30.4)	15.2% (4.2-31.9)	16.5% (4.5-34.2)	15.3% (4.1-31.9)
Gabon*	15.5% (4.1-31.1)	16.6% (4.6-29.9)	21.4% (10.5-36.0)	15.3% (4.2-30.5)	15.3% (4.1-31.1)	16.7% (4.4-33.0)	15.5% (4.0-30.7)
Gambia*	15.7% (4.5-32.3)	17.0% (4.8-31.2)	21.9% (10.6-37.0)	15.5% (4.3-31.7)	15.9% (4.1-32.8)	17.0% (4.7-34.8)	15.9% (4.4-32.8)
Ghana*	13.1% (3.5-26.5)	15.4% (4.4-28.3)	18.2% (9.0-30.8)	12.8% (3.6-26.2)	13.3% (3.6-26.9)	13.7% (3.8-27.9)	13.2% (3.5-26.7)
Guinea*	15.8% (4.5-31.2)	17.1% (4.9-30.2)	21.9% (10.3-35.8)	15.4% (4.5-30.5)	15.8% (4.2-31.4)	16.9% (4.9-33.3)	15.8% (4.4-31.2)
Guinea-Bissau*	15.4% (4.2-31.1)	16.6% (4.8-30.2)	21.3% (10.7-35.6)	15.2% (4.2-30.6)	15.4% (4.2-31.1)	16.6% (4.6-33.2)	15.4% (4.2-31.2)
Kenya	9.8% (2.5-20.6)	8.9% (2.4-19.2)	13.8% (6.5-23.8)	10.3% (2.9-20.9)	9.1% (2.5-19.8)	9.9% (2.7-21.3)	9.8% (2.6-20.7)
Lesotho*	13.2% (3.6-26.4)	15.5% (4.4-27.8)	18.4% (8.8-30.6)	13.0% (3.7-26.0)	13.3% (3.7-26.9)	14.0% (3.9-28.0)	13.2% (3.6-26.4)
Liberia*	15.4% (4.4-32.1)	16.5% (5.0-30.9)	21.2% (10.4-36.9)	15.3% (4.1-31.5)	15.3% (4.3-32.1)	16.5% (4.6-34.9)	15.4% (4.3-32.1)
Madagascar*	18.4% (5.4-36.8)	23.6% (7.5-39.2)	25.0% (13.0-41.7)	17.3% (4.9-34.6)	19.3% (5.2-38.9)	19.8% (5.9-39.9)	18.4% (5.3-37.2)
Malawi*	14.3% (3.8-29.6)	16.2% (4.6-30.0)	19.9% (9.6-33.6)	14.1% (3.7-29.0)	14.6% (4.0-29.9)	15.4% (4.2-31.5)	14.4% (3.9-29.9)
Mali*	15.8% (4.3-31.4)	17.0% (4.9-30.2)	22.0% (10.3-36.5)	15.4% (4.0-31.7)	16.0% (4.2-31.6)	17.2% (4.6-34.1)	16.0% (4.2-31.8)
Mauritania*	15.5% (4.1-31.4)	16.6% (4.9-30.9)	21.4% (10.2-35.5)	15.3% (4.1-30.6)	15.4% (4.0-31.8)	16.9% (4.5-33.6)	15.5% (4.1-31.8)
Mauritius*	9.2% (2.4-19.8)	8.5% (2.2-18.0)	13.0% (6.0-23.0)	9.6% (2.5-20.6)	8.7% (2.3-18.8)	9.3% (2.4-19.8)	9.2% (2.4-20.0)
Mozambique*	9.4% (2.5-20.2)	8.4% (2.2-17.1)	13.4% (6.2-24.1)	10.3% (2.7-21.8)	8.7% (2.3-18.8)	9.6% (2.6-20.6)	9.5% (2.5-20.2)
Namibia*	13.9% (3.9-29.4)	15.9% (4.5-30.2)	19.4% (9.5-33.5)	13.8% (3.6-29.1)	13.9% (4.0-29.6)	14.9% (4.2-31.4)	14.0% (3.7-29.4)
Niger*	15.9% (3.9-31.4)	16.9% (4.5-30.8)	21.8% (10.0-36.2)	15.6% (3.9-30.9)	15.8% (3.8-31.6)	17.2% (4.3-34.0)	16.0% (3.8-32.0)
Nigeria*	14.6% (3.9-30.1)	23.9% (7.7-38.6)	20.3% (9.4-34.7)	13.9% (3.6-28.9)	15.3% (4.0-31.9)	15.5% (4.2-32.3)	14.7% (3.8-30.4)
Rep. of the Congo*	15.6% (4.2-31.6)	16.9% (4.7-30.9)	21.7% (9.9-36.3)	15.4% (4.0-31.2)	15.7% (4.1-31.5)	16.9% (4.6-34.2)	15.8% (4.2-32.0)
Rwanda*	14.9% (4.1-30.9)	16.6% (4.8-30.2)	20.5% (10.2-35.3)	14.6% (4.5-29.8)	15.0% (4.1-30.7)	16.0% (4.5-32.8)	14.9% (4.0-30.9)
Sao Tome & Principe*	15.7% (4.2-31.7)	17.1% (4.7-30.7)	21.5% (10.3-36.4)	15.5% (4.2-31.2)	15.7% (4.3-31.8)	17.1% (4.5-34.4)	15.8% (4.2-31.8)
Senegal*	16.8% (4.4-34.6)	20.4% (6.3-36.3)	23.3% (10.6-39.6)	16.1% (4.2-32.9)	17.3% (4.5-36.1)	18.6% (4.7-37.4)	17.0% (4.3-34.7)
Seychelles*	17.9% (4.8-37.2)	23.9% (7.7-39.0)	24.7% (11.9-42.7)	17.0% (4.4-35.8)	18.9% (5.0-39.1)	19.7% (5.2-40.0)	18.1% (4.8-37.6)
Sierra Leone*	17.3% (4.6-33.5)	21.3% (6.3-35.8)	23.7% (11.0-38.4)	16.5% (4.4-32.0)	18.0% (5.0-34.9)	18.8% (5.3-36.5)	17.4% (4.7-34.1)
Somalia*	16.1% (4.1-31.1)	17.0% (4.4-30.3)	22.1% (10.2-36.1)	16.0% (4.1-30.5)	16.0% (4.0-31.4)	17.4% (4.6-33.5)	16.2% (4.2-31.5)
South Africa	20.3% (5.8-36.8)	21.0% (6.7-35.1)	27.1% (14.1-42.1)	19.7% (5.5-35.7)	20.8% (6.2-37.8)	21.2% (6.3-38.9)	20.5% (5.9-37.4)
Swaziland*	13.4% (3.7-26.5)	15.5% (4.4-28.4)	18.6% (8.9-31.4)	13.1% (3.3-25.9)	13.7% (3.8-27.3)	14.4% (3.9-28.2)	13.4% (3.7-26.7)
Togo*	8.4% (2.2-17.1)	10.7% (3.0-21.2)	11.8% (5.9-20.3)	8.4% (2.2-17.0)	8.3% (2.1-17.0)	9.2% (2.5-19.2)	8.4% (2.2-17.2)
Uganda*	14.8% (3.9-30.6)	16.3% (4.6-30.6)	20.3% (9.9-35.1)	14.6% (3.9-30.3)	14.8% (4.0-30.6)	15.9% (4.4-32.8)	14.8% (4.0-30.7)
United Rep. of Tanzania	12.2% (3.0-26.5)	12.0% (3.1-24.9)	16.9% (7.4-30.6)	12.5% (3.0-26.8)	11.6% (3.0-26.0)	13.0% (3.3-28.4)	12.1% (3.0-26.5)
Zambia*	13.9% (3.7-28.7)	15.8% (4.7-29.2)	19.2% (9.7-33.2)	13.7% (3.6-28.1)	14.1% (4.0-29.4)	14.9% (4.1-30.9)	13.9% (3.9-29.1)
Zimbabwe*	13.9% (3.6-27.9)	15.7% (4.6-29.5)	19.3% (9.2-32.3)	13.8% (3.7-27.5)	14.1% (4.0-28.3)	14.9% (4.2-29.5)	14.1% (3.8-27.8)
**Western Europe**							
Albania	15.4% (4.3-28.0)	25.5% (8.5-40.7)	20.8% (11.0-32.2)	14.6% (4.0-26.8)	16.1% (4.7-29.3)	16.1% (4.7-29.6)	15.4% (4.5-28.0)
Andorra*	11.8% (3.3-22.7)	21.5% (6.7-35.3)	16.5% (8.4-26.2)	11.4% (3.1-22.0)	12.2% (3.4-23.3)	12.4% (3.4-23.9)	11.8% (3.3-22.5)
Austria	8.4% (2.2-16.9)	25.2% (8.3-40.0)	11.8% (5.5-20.0)	8.1% (1.9-16.5)	8.8% (2.2-17.6)	8.8% (2.4-17.9)	8.3% (2.3-16.8)
Belgium	15.0% (4.1-29.1)	23.1% (7.4-37.7)	20.6% (10.4-33.0)	14.5% (3.7-28.2)	15.6% (4.3-30.0)	15.7% (4.3-30.1)	15.2% (4.2-29.3)
Croatia	8.6% (2.2-18.4)	25.8% (8.3-40.6)	12.1% (5.7-21.6)	8.4% (1.9-17.7)	9.0% (2.2-19.1)	9.1% (2.4-19.1)	8.6% (2.2-18.4)
Denmark	13.3% (3.8-26.6)	24.9% (8.2-39.6)	18.3% (9.2-30.0)	12.9% (3.7-25.6)	13.8% (3.7-27.5)	13.7% (3.9-27.3)	13.4% (3.7-26.4)
England	20.4% (6.1-35.9)	26.3% (8.7-41.4)	27.3% (15.2-40.6)	19.6% (5.6-34.6)	21.2% (6.5-37.3)	21.3% (6.5-37.4)	20.7% (6.4-36.4)
Finland*	12.0% (3.4-22.8)	25.1% (8.2-39.7)	16.6% (8.7-26.6)	11.4% (3.2-22.2)	12.6% (3.5-24.0)	12.7% (3.6-24.5)	12.0% (3.5-23.2)
Form. Yug. Rep. Macedonia*	11.7% (3.4-22.7)	21.5% (6.8-35.1)	16.2% (8.7-26.4)	11.3% (3.0-21.8)	12.3% (3.5-23.8)	12.3% (3.5-24.2)	11.8% (3.3-22.9)
France	6.2% (1.6-12.9)	12.7% (3.5-24.2)	8.9% (4.3-15.2)	6.2% (1.4-12.7)	6.4% (1.6-13.4)	6.6% (1.7-13.8)	6.2% (1.6-12.9)
Germany	7.8% (2.1-15.7)	19.0% (5.9-32.3)	10.9% (5.6-18.4)	7.6% (1.9-15.7)	8.2% (2.3-16.4)	8.1% (2.3-16.8)	7.7% (2.1-15.9)
Greece	14.8% (4.4-27.7)	21.5% (6.9-35.7)	20.2% (10.9-31.9)	14.2% (4.2-27.1)	15.4% (4.5-28.8)	15.5% (4.6-29.2)	15.0% (4.4-28.1)
Greenland*	11.7% (3.2-22.9)	21.3% (7.0-35.5)	16.3% (8.8-26.5)	11.5% (3.1-22.0)	12.0% (3.5-23.8)	12.4% (3.6-24.0)	11.7% (3.3-23.3)

	Main Analyses	Assortative Mixing	Unadjusted RR	Decreasing incidence	Increasing incidence	All PWID start injecting stably housed	Unstably housed PWID have higher mortality rates
Iceland*	20.8% (6.3-36.6)	24.5% (8.0-39.3)	27.8% (15.5-41.2)	19.8% (6.0-35.1)	21.6% (6.5-38.3)	22.0% (6.8-39.3)	21.0% (6.2-36.8)
Ireland	3.2% (0.8-7.0)	10.2% (2.7-19.7)	4.6% (2.2-8.5)	3.3% (0.4-7.1)	3.4% (0.5-7.4)	3.5% (0.9-7.7)	3.2% (0.8-6.9)
Italy	8.6% (2.4-17.1)	17.8% (5.5-30.3)	12.2% (6.4-19.8)	8.2% (1.9-16.5)	9.1% (2.3-17.6)	9.3% (2.6-18.4)	8.6% (2.3-17.1)
Liechtenstein*	11.8% (3.4-22.9)	21.3% (6.7-35.4)	16.5% (8.8-26.6)	11.4% (3.3-22.3)	12.2% (3.5-23.5)	12.5% (3.6-24.1)	11.8% (3.4-22.9)
Luxembourg*	12.1% (3.5-23.1)	24.9% (8.2-39.4)	16.8% (9.0-26.5)	11.6% (3.2-22.0)	12.7% (3.7-24.0)	12.8% (3.7-24.5)	12.1% (3.4-23.4)
Malta*	12.2% (3.3-23.5)	26.0% (8.5-40.9)	16.7% (8.8-27.0)	11.6% (3.0-22.6)	12.8% (3.5-24.5)	12.8% (3.5-24.8)	12.2% (3.4-23.5)
Monaco*	11.8% (3.3-23.4)	21.5% (6.8-35.8)	16.5% (8.8-26.8)	11.4% (3.0-22.5)	12.4% (3.4-24.7)	12.5% (3.6-25.2)	11.9% (3.4-23.9)
Montenegro*	14.7% (4.0-28.3)	26.4% (8.8-41.5)	20.2% (10.4-32.8)	13.7% (3.7-27.0)	15.6% (4.0-30.2)	16.1% (4.3-31.6)	14.6% (4.0-28.4)
Netherlands	7.5% (2.1-14.7)	21.6% (6.8-35.5)	10.7% (5.7-17.4)	7.3% (2.0-14.3)	7.8% (2.1-15.5)	8.0% (2.2-15.9)	7.5% (2.1-14.7)
Northern Ireland*	12.0% (3.2-22.6)	21.6% (6.8-35.4)	16.5% (8.4-25.8)	11.5% (3.0-21.8)	12.4% (3.4-23.8)	12.7% (3.3-23.9)	12.0% (3.2-22.7)
Norway	13.4% (3.9-26.5)	25.9% (8.6-40.6)	18.8% (10.0-30.3)	13.0% (3.5-25.7)	14.1% (4.2-27.7)	14.0% (4.1-27.8)	13.6% (3.8-26.6)
Portugal*	10.7% (3.0-20.8)	13.7% (3.9-24.6)	14.9% (7.9-23.9)	10.6% (2.9-20.7)	10.9% (3.0-21.4)	11.2% (3.1-21.8)	10.7% (3.0-20.8)
San Marino*	11.8% (3.3-22.9)	21.5% (6.6-35.4)	16.5% (8.5-26.2)	11.5% (3.1-22.1)	12.3% (3.4-23.8)	12.6% (3.6-24.3)	11.9% (3.3-23.2)
Scotland	13.3% (3.5-24.7)	25.7% (8.5-40.7)	18.3% (9.5-28.1)	12.8% (3.3-23.8)	13.9% (4.0-25.8)	14.0% (3.8-25.9)	13.3% (3.7-24.7)
Serbia	NA	NA	NA	NA	NA	NA	NA
Slovenia	5.0% (1.2-11.2)	24.7% (8.0-39.0)	7.1% (3.1-13.3)	4.9% (0.8-10.8)	5.2% (1.0-11.4)	5.3% (1.3-12.2)	4.9% (1.2-10.8)
Spain	10.3% (3.0-20.7)	10.6% (3.1-20.6)	14.5% (7.3-24.1)	10.5% (3.0-20.6)	10.3% (2.9-20.6)	10.6% (3.0-21.5)	10.3% (2.9-20.8)
Sweden	15.9% (4.6-29.7)	26.4% (8.9-41.7)	21.6% (11.5-33.6)	15.4% (4.3-29.1)	16.4% (4.8-30.9)	16.4% (4.6-30.8)	16.2% (4.7-30.4)
Switzerland	7.5% (2.0-15.0)	23.3% (7.7-37.9)	10.6% (5.5-17.8)	7.4% (1.8-14.5)	7.8% (2.1-15.8)	8.0% (2.2-16.3)	7.5% (2.0-15.0)
Wales*	23.7% (7.2-39.7)	25.2% (8.4-40.0)	31.1% (18.1-43.9)	22.8% (6.5-38.4)	24.6% (7.7-41.1)	24.5% (7.5-41.2)	24.0% (7.4-39.9)

Appendix Table 7: Sensitivity analysis for the tPAFs of Unstable Housing on HCV. *Countries marked with an asterisk denotes that the country had 'insufficient data' and so regional data for were used.*

	**Main Analyses**	**Assortative Mixing**	**Higher RR in Europe**	**Decreasing incidence**	**Increasing incidence**	**All PWID start injecting stably housed**	**Treat 10% infected PWID per year**	**Unstably housed PWID have higher mortality rates**
**Australasia**								
Australia	11.1% (7.3-15.1)	11.4% (7.6-15.4)	11.1% (7.3-15.1)	11.2% (7.5-15.3)	11.0% (7.4-14.9)	11.2% (7.6-15.4)	11.8% (7.8-16.1)	11.1% (7.4-15.2)
New Zealand	7.3% (4.2-12.0)	6.8% (4.0-11.3)	7.3% (4.2-12.0)	7.5% (4.4-12.2)	7.2% (4.2-11.8)	7.3% (4.2-11.9)	8.3% (4.7-13.5)	7.3% (4.3-11.8)
**Caribbean**								
Antigua & Barbuda*	12.6% (9.0-16.6)	11.9% (8.5-16.0)	12.6% (9.0-16.6)	12.9% (9.3-17.1)	12.3% (8.7-16.3)	12.7% (9.1-16.8)	13.9% (9.9-18.5)	12.6% (9.0-16.8)
Bahamas*	12.6% (8.9-16.9)	12.1% (8.3-16.3)	12.6% (8.9-16.9)	12.9% (9.0-17.4)	12.3% (8.5-16.5)	12.7% (8.8-17.1)	13.9% (9.7-18.4)	12.6% (8.8-16.9)
Barbados*	12.6% (8.7-16.9)	12.0% (8.2-16.2)	12.6% (8.7-16.9)	13.0% (9.1-17.3)	12.3% (8.5-16.6)	12.7% (8.8-17.0)	13.9% (9.6-18.5)	12.7% (8.9-17.0)
Bermuda*	12.7% (9.0-16.8)	12.0% (8.5-16.2)	12.7% (9.0-16.8)	13.0% (9.1-17.4)	12.4% (8.7-16.5)	12.8% (8.9-17.1)	14.0% (9.6-18.6)	12.7% (8.9-16.9)
Comm. of Puerto Rico	10.7% (7.5-14.6)	9.6% (6.7-13.2)	10.7% (7.5-14.6)	11.2% (7.9-15.1)	10.1% (7.2-14.0)	10.4% (7.3-14.3)	12.7% (8.7-17.0)	10.7% (7.5-14.6)
Cuba*	12.6% (8.9-17.0)	12.0% (8.5-16.1)	12.6% (8.9-17.0)	13.0% (9.2-17.4)	12.3% (8.7-16.6)	12.7% (9.0-17.3)	13.9% (9.9-18.6)	12.7% (9.0-17.1)
Dominica*	12.6% (9.0-16.7)	12.0% (8.5-16.0)	12.6% (9.0-16.7)	12.9% (9.2-17.1)	12.3% (8.8-16.3)	12.7% (9.0-16.9)	13.9% (9.9-18.5)	12.7% (9.0-16.7)
Dominican Republic*	12.7% (9.0-16.7)	12.0% (8.4-16.1)	12.7% (9.0-16.7)	13.0% (9.3-17.1)	12.3% (8.8-16.4)	12.7% (9.0-16.8)	14.0% (10.0-18.6)	12.7% (9.0-16.7)
Grenada*	12.5% (8.9-17.1)	11.9% (8.2-16.2)	12.5% (8.9-17.1)	12.8% (9.2-17.4)	12.3% (8.6-16.7)	12.7% (8.9-17.2)	13.9% (9.7-18.7)	12.6% (8.9-17.1)
Haiti*	12.5% (8.8-16.8)	11.8% (8.4-16.3)	12.5% (8.8-16.8)	12.8% (9.2-17.2)	12.2% (8.6-16.6)	12.6% (8.9-17.1)	13.8% (9.8-18.4)	12.5% (8.9-16.9)
Jamaica*	12.7% (9.0-17.0)	11.9% (8.4-16.1)	12.7% (9.0-17.0)	13.0% (9.1-17.2)	12.2% (8.6-16.6)	12.7% (8.9-17.0)	14.0% (9.8-18.5)	12.7% (9.0-16.9)
Saint Kitts & Nevis*	12.6% (8.9-17.1)	11.9% (8.5-16.2)	12.6% (8.9-17.1)	13.0% (9.3-17.6)	12.3% (8.7-16.7)	12.6% (9.0-17.3)	13.9% (9.8-18.7)	12.6% (9.1-17.2)
Saint Lucia*	12.7% (8.9-16.9)	12.0% (8.4-16.1)	12.7% (8.9-16.9)	13.0% (9.1-17.3)	12.3% (8.7-16.4)	12.7% (8.8-17.0)	14.0% (10.1-18.6)	12.7% (8.9-16.9)
St Vincent & the Grenadines*	12.7% (9.0-17.0)	12.0% (8.5-16.2)	12.7% (9.0-17.0)	13.0% (9.3-17.4)	12.4% (8.8-16.6)	12.8% (9.0-17.1)	14.0% (9.9-18.6)	12.7% (9.1-17.0)
Trinidad & Tobago*	12.5% (8.8-16.7)	11.9% (8.2-15.8)	12.5% (8.8-16.7)	12.9% (9.1-17.1)	12.2% (8.5-16.2)	12.6% (8.8-16.9)	13.9% (9.5-18.4)	12.6% (8.7-16.7)
**Central Asia**								
Kazakhstan	9.4% (6.6-12.9)	8.7% (6.1-12.0)	9.4% (6.6-12.9)	10.0% (6.8-13.7)	8.8% (6.1-12.1)	9.6% (6.6-13.3)	10.3% (7.1-14.2)	9.4% (6.5-12.8)
Kyrgyzstan	13.8% (9.6-18.7)	14.1% (10.1-18.6)	13.8% (9.6-18.7)	14.0% (9.6-19.1)	13.5% (9.6-18.1)	14.4% (10.0-19.6)	14.4% (10.2-19.4)	13.8% (9.6-18.6)
Tajikistan*	9.4% (6.5-12.7)	8.7% (6.1-11.8)	9.4% (6.5-12.7)	9.9% (6.9-13.6)	8.9% (6.1-12.0)	9.6% (6.5-13.0)	10.4% (7.1-14.1)	9.4% (6.4-12.8)
Turkmenistan*	12.1% (8.1-17.3)	13.3% (8.2-20.6)	12.1% (8.1-17.3)	12.3% (8.2-17.4)	11.9% (7.7-17.4)	12.9% (8.4-18.9)	12.7% (8.5-17.8)	12.1% (8.1-17.3)
Uzbekistan*	10.7% (7.5-14.8)	10.4% (7.3-14.2)	10.7% (7.5-14.8)	11.1% (7.7-15.4)	10.3% (7.3-14.1)	11.1% (7.8-15.7)	11.5% (8.1-15.9)	10.7% (7.5-14.7)
**East and South East Asia**								
Brunei Darussalam*	6.9% (4.4-10.5)	7.0% (4.5-10.8)	6.9% (4.4-10.5)	7.1% (4.5-10.8)	6.7% (4.1-10.1)	7.2% (4.5-11.0)	7.4% (4.6-11.0)	6.9% (4.4-10.5)
Cambodia*	12.6% (8.8-17.1)	12.4% (8.5-17.3)	12.6% (8.8-17.1)	12.9% (8.9-17.4)	12.2% (8.5-16.9)	12.9% (8.9-18.0)	13.5% (9.4-18.3)	12.6% (8.7-17.0)
China	7.4% (4.9-11.4)	8.0% (5.2-12.7)	7.4% (4.9-11.4)	7.6% (5.0-11.4)	7.2% (4.6-11.2)	7.8% (5.1-12.2)	7.8% (5.1-11.8)	7.4% (4.9-11.3)
Hong Kong (China)*	4.3% (2.7-6.5)	3.8% (2.4-5.7)	4.3% (2.7-6.5)	4.6% (3.0-6.9)	4.0% (2.5-6.1)	4.1% (2.6-6.3)	5.3% (2.9-8.2)	4.3% (2.8-6.5)
Indonesia*	3.5% (2.2-5.4)	3.0% (1.9-4.7)	3.5% (2.2-5.4)	3.9% (2.5-6.0)	3.1% (1.9-4.9)	3.2% (1.9-5.1)	4.5% (2.6-6.9)	3.5% (2.2-5.4)
Japan*	5.7% (3.6-9.0)	5.3% (3.3-8.4)	5.7% (3.6-9.0)	6.1% (3.8-9.4)	5.4% (3.3-8.5)	5.8% (3.6-9.1)	6.5% (3.9-10.1)	5.7% (3.6-9.0)
Lao PDR*	6.8% (4.4-10.5)	6.9% (4.4-11.0)	6.8% (4.4-10.5)	7.1% (4.4-10.8)	6.6% (4.1-10.4)	7.1% (4.5-11.0)	7.4% (4.7-11.2)	6.8% (4.4-10.5)
Malaysia*	5.5% (3.4-8.1)	5.2% (3.2-7.6)	5.5% (3.4-8.1)	5.8% (3.6-8.4)	5.3% (3.3-7.8)	5.5% (3.4-8.2)	6.2% (3.7-9.4)	5.5% (3.5-8.1)
Mongolia*	6.9% (4.4-10.6)	7.0% (4.4-11.0)	6.9% (4.4-10.6)	7.1% (4.4-10.8)	6.7% (4.1-10.3)	7.1% (4.6-11.1)	7.5% (4.7-11.4)	6.9% (4.4-10.5)
Myanmar*	10.3% (6.7-16.1)	12.3% (8.3-18.2)	10.3% (6.7-16.1)	10.3% (6.5-16.6)	10.1% (6.3-15.8)	11.6% (7.2-18.0)	10.6% (6.9-16.5)	10.3% (6.6-16.2)
North Korea*	6.9% (4.3-10.4)	7.1% (4.3-10.8)	6.9% (4.3-10.4)	7.1% (4.3-10.7)	6.7% (4.1-10.2)	7.2% (4.4-11.0)	7.4% (4.6-11.2)	6.9% (4.3-10.5)
Philippines*	8.1% (4.9-12.4)	9.6% (5.7-15.8)	8.1% (4.9-12.4)	8.1% (5.0-12.5)	8.0% (4.9-12.3)	8.6% (5.2-13.4)	8.4% (5.2-12.7)	8.1% (4.9-12.3)
Republic of Korea*	7.5% (4.6-11.5)	7.5% (4.8-11.4)	7.5% (4.6-11.5)	7.8% (4.8-12.1)	7.1% (4.4-10.9)	8.0% (4.9-12.3)	8.0% (5.0-12.2)	7.5% (4.7-11.4)
Singapore*	7.4% (4.7-11.5)	8.2% (5.4-12.6)	7.4% (4.7-11.5)	7.6% (4.8-11.7)	7.3% (4.6-11.3)	7.8% (5.0-12.2)	7.8% (4.9-12.1)	7.4% (4.8-11.5)
Taiwan	0.3% (0.2-0.6)	0.3% (0.1-0.5)	0.3% (0.2-0.6)	0.4% (0.1-0.7)	0.3% (0.0-0.5)	0.3% (0.1-0.5)	0.4% (-0.8-2.2)	0.3% (0.2-0.6)

	Main Analyses	Assortative Mixing	Higher RR in Europe	Decreasing incidence	Increasing incidence	All PWID start injecting stably housed	Treat 10% infected PWID per year	Unstably housed PWID have higher mortality rates
Thailand*	3.7% (2.3-5.9)	3.2% (1.9-5.1)	3.7% (2.3-5.9)	4.1% (2.6-6.4)	3.3% (2.0-5.3)	3.3% (2.0-5.5)	4.6% (2.6-7.4)	3.7% (2.3-5.9)
Timor Leste*	6.8% (4.3-10.2)	6.9% (4.4-10.8)	6.8% (4.3-10.2)	7.1% (4.5-10.6)	6.7% (4.1-9.8)	7.1% (4.4-10.6)	7.4% (4.6-10.9)	6.8% (4.4-10.2)
Viet Nam	7.4% (1.6-16.7)	7.0% (1.5-15.8)	7.4% (1.6-16.7)	7.8% (1.7-17.3)	7.0% (1.4-16.0)	7.6% (1.6-17.0)	8.2% (1.7-18.0)	7.4% (1.6-16.7)
**Eastern Europe**								
Armenia*	5.3% (3.5-8.0)	6.1% (3.9-9.4)	8.6% (5.3-13.6)	5.5% (3.4-8.4)	5.3% (3.3-8.0)	5.6% (3.6-8.5)	5.6% (3.6-8.4)	5.3% (3.4-8.1)
Azerbaijan*	4.3% (2.8-6.6)	4.2% (2.6-6.5)	6.9% (4.0-11.2)	4.6% (2.8-7.1)	4.2% (2.5-6.2)	4.4% (2.8-6.8)	4.9% (2.8-7.4)	4.3% (2.8-6.6)
Belarus*	4.5% (2.8-6.8)	4.5% (2.7-6.9)	7.2% (4.4-11.3)	4.7% (2.9-7.1)	4.4% (2.6-6.6)	4.6% (2.8-7.0)	5.0% (2.9-7.6)	4.5% (2.9-6.8)
Bosnia & Herzegovina*	5.1% (3.3-7.7)	6.1% (3.9-9.5)	8.1% (4.9-12.4)	5.2% (3.2-7.7)	5.1% (3.3-7.7)	5.2% (3.5-8.1)	5.3% (3.4-7.9)	5.1% (3.3-7.6)
Bulgaria	1.9% (1.3-2.7)	1.8% (1.2-2.5)	3.1% (2.0-4.5)	2.1% (1.3-3.1)	1.8% (1.1-2.7)	1.9% (1.3-2.7)	2.3% (0.9-3.8)	1.9% (1.3-2.7)
Czech Republic	32.0% (24.1-41.1)	33.4% (26.1-40.2)	43.9% (32.9-54.4)	31.2% (23.5-39.8)	32.7% (24.7-42.1)	33.1% (24.9-42.4)	32.2% (24.4-41.3)	32.7% (24.7-41.7)
Estonia*	3.4% (2.1-5.3)	3.0% (1.9-4.7)	5.3% (3.3-8.4)	3.6% (2.3-5.6)	3.1% (1.9-5.0)	3.2% (2.0-5.2)	4.1% (2.3-6.4)	3.4% (2.1-5.2)
Georgia	2.0% (0.9-3.6)	1.8% (0.8-3.4)	3.1% (1.3-5.9)	2.1% (0.8-3.8)	1.9% (0.7-3.4)	1.9% (0.9-3.5)	2.2% (0.5-4.3)	2.0% (0.9-3.6)
Hungary	13.7% (6.7-21.4)	14.1% (6.8-22.0)	20.6% (10.2-32.2)	13.8% (6.9-21.5)	13.5% (6.3-21.5)	14.0% (6.8-22.0)	14.4% (6.9-22.3)	13.7% (6.5-21.7)
Latvia	0.8% (0.3-1.5)	0.7% (0.3-1.4)	1.3% (0.5-2.5)	0.9% (0.3-1.7)	0.7% (0.2-1.5)	0.8% (0.3-1.5)	1.0% (-0.3-2.4)	0.8% (0.3-1.5)
Lithuania	4.9% (3.1-7.4)	5.6% (3.7-8.3)	7.8% (4.9-12.4)	5.0% (3.1-7.6)	4.9% (3.0-7.4)	5.1% (3.3-7.8)	5.1% (3.2-7.8)	4.9% (3.1-7.4)
Moldova	5.2% (3.2-7.8)	5.5% (3.3-8.6)	8.1% (5.1-13.0)	5.3% (3.2-8.0)	5.1% (3.1-7.7)	5.3% (3.3-8.0)	5.5% (3.3-8.4)	5.2% (3.2-7.7)
Poland	11.3% (8.2-15.0)	11.2% (8.1-14.6)	17.2% (12.0-23.4)	11.5% (8.2-15.1)	11.2% (8.1-14.6)	11.5% (8.2-15.1)	12.3% (8.9-16.1)	11.4% (8.2-15.0)
Romania	7.5% (2.3-13.6)	6.6% (2.0-12.1)	11.6% (3.4-21.5)	8.1% (2.6-14.4)	7.0% (2.0-12.9)	7.2% (2.1-13.2)	9.3% (2.7-16.5)	7.5% (2.3-13.6)
Russian Federation	2.7% (1.4-4.2)	2.4% (1.3-3.9)	4.2% (2.2-7.0)	2.9% (1.5-4.6)	2.5% (1.3-4.0)	2.7% (1.4-4.2)	3.1% (1.4-5.2)	2.7% (1.4-4.3)
Slovakia*	4.7% (2.9-7.1)	4.7% (2.8-7.5)	7.4% (4.4-11.6)	4.9% (3.0-7.4)	4.5% (2.7-7.0)	4.8% (2.9-7.4)	5.1% (3.1-7.8)	4.7% (2.9-7.1)
Ukraine	0.3% (0.2-0.6)	0.3% (0.2-0.6)	0.5% (0.3-1.0)	0.4% (-0.2-1.3)	0.3% (-0.3-0.9)	0.3% (0.2-0.6)	0.3% (-0.6-1.5)	0.3% (0.2-0.6)
**Latin America**								
Argentina*	12.1% (7.8-16.9)	12.3% (8.1-17.0)	12.1% (7.8-16.9)	12.2% (7.8-17.1)	12.0% (8.0-16.7)	12.3% (8.0-17.3)	12.9% (8.4-18.1)	12.1% (7.9-17.1)
Belize*	11.6% (7.6-16.3)	11.3% (7.4-16.0)	11.6% (7.6-16.3)	11.8% (7.6-16.7)	11.4% (7.4-16.1)	11.7% (7.5-16.5)	12.6% (8.1-17.7)	11.6% (7.6-16.5)
Bolivia*	11.4% (7.7-16.3)	11.2% (7.5-15.9)	11.4% (7.7-16.3)	11.7% (7.8-16.5)	11.3% (7.6-16.0)	11.5% (7.8-16.4)	12.5% (8.2-17.6)	11.4% (7.6-16.4)
Brazil*	11.4% (7.6-16.0)	11.0% (7.4-15.5)	11.4% (7.6-16.0)	11.7% (7.7-16.3)	11.2% (7.6-15.7)	11.4% (7.7-16.1)	12.4% (8.3-17.3)	11.4% (7.7-16.0)
Chile*	11.5% (7.6-16.3)	11.2% (7.4-15.9)	11.5% (7.6-16.3)	11.7% (7.7-16.6)	11.3% (7.5-16.1)	11.6% (7.7-16.4)	12.5% (8.0-17.5)	11.5% (7.6-16.3)
Colombia*	20.9% (13.3-30.9)	21.9% (15.1-29.1)	20.9% (13.3-30.9)	20.9% (13.0-31.1)	20.5% (13.2-29.8)	22.9% (14.4-34.0)	21.3% (13.6-31.4)	20.8% (13.3-30.7)
Costa Rica*	11.6% (7.5-16.0)	11.3% (7.5-15.7)	11.6% (7.5-16.0)	11.8% (7.7-16.3)	11.4% (7.5-15.8)	11.7% (7.6-16.1)	12.6% (8.0-17.4)	11.6% (7.6-16.1)
Ecuador*	11.5% (7.5-15.8)	11.2% (7.3-15.6)	11.5% (7.5-15.8)	11.7% (7.5-16.1)	11.3% (7.5-15.6)	11.5% (7.5-15.9)	12.4% (8.3-17.4)	11.5% (7.6-16.0)
El Salvador*	11.5% (7.6-16.5)	11.2% (7.3-15.9)	11.5% (7.6-16.5)	11.7% (7.7-16.8)	11.4% (7.5-16.3)	11.5% (7.6-16.7)	12.6% (8.3-17.8)	11.5% (7.6-16.4)
Guatemala*	11.5% (7.6-16.2)	11.2% (7.4-15.6)	11.5% (7.6-16.2)	11.7% (7.8-16.4)	11.3% (7.6-15.9)	11.6% (7.8-16.3)	12.5% (8.2-17.5)	11.6% (7.7-16.2)
Guyana*	11.4% (7.4-16.1)	11.1% (7.3-15.6)	11.4% (7.4-16.1)	11.6% (7.6-16.3)	11.2% (7.3-15.8)	11.4% (7.4-16.2)	12.4% (8.1-17.4)	11.4% (7.5-16.2)
Honduras*	11.4% (7.8-16.1)	11.1% (7.6-15.7)	11.4% (7.8-16.1)	11.6% (7.9-16.3)	11.2% (7.7-15.9)	11.4% (7.7-16.2)	12.4% (8.3-17.5)	11.4% (7.8-16.1)
Mexico	7.8% (5.0-11.5)	6.8% (4.3-10.1)	7.8% (5.0-11.5)	8.3% (5.4-12.0)	7.3% (4.6-10.9)	7.3% (4.5-11.0)	10.3% (6.3-14.6)	7.8% (4.9-11.4)
Nicaragua*	12.0% (8.1-17.4)	11.1% (7.4-16.1)	12.0% (8.1-17.4)	12.6% (8.5-18.0)	11.4% (7.6-16.6)	12.2% (8.1-17.5)	13.3% (8.9-19.1)	12.1% (8.1-17.4)
Panama*	11.4% (7.6-16.1)	11.1% (7.4-15.5)	11.4% (7.6-16.1)	11.6% (7.7-16.5)	11.3% (7.5-15.8)	11.5% (7.6-16.1)	12.4% (8.3-17.4)	11.4% (7.6-16.1)
Paraguay*	14.0% (9.3-19.8)	26.0% (19.3-32.8)	14.0% (9.3-19.8)	13.8% (9.1-19.6)	14.3% (9.5-20.1)	14.6% (9.7-20.6)	14.0% (9.4-19.8)	14.1% (9.3-19.9)
Peru*	11.5% (7.6-16.0)	11.2% (7.5-15.8)	11.5% (7.6-16.0)	11.6% (7.7-16.5)	11.3% (7.6-15.8)	11.5% (7.7-16.3)	12.5% (8.3-17.5)	11.4% (7.7-16.1)
Suriname*	11.5% (7.7-16.0)	11.3% (7.4-15.8)	11.5% (7.7-16.0)	11.7% (7.8-16.1)	11.4% (7.5-15.9)	11.6% (7.7-16.3)	12.5% (8.2-17.3)	11.6% (7.6-16.1)
Uruguay*	13.6% (9.0-19.8)	19.4% (13.7-26.3)	13.6% (9.0-19.8)	13.5% (9.2-19.4)	13.8% (9.2-20.0)	14.1% (9.4-20.6)	13.7% (9.2-19.8)	13.6% (9.1-19.9)
Venezuela*	11.5% (7.6-16.4)	11.2% (7.3-15.9)	11.5% (7.6-16.4)	11.7% (7.6-16.6)	11.3% (7.4-16.2)	11.6% (7.5-16.5)	12.5% (8.3-17.6)	11.5% (7.5-16.5)
**Middle East & North Africa**								
Algeria*	7.0% (3.8-11.1)	7.3% (4.1-11.5)	7.0% (3.8-11.1)	7.1% (3.9-11.3)	6.9% (3.7-10.8)	7.2% (4.0-11.6)	7.4% (3.9-11.7)	7.0% (3.8-11.1)
Bahrain*	6.9% (3.7-10.9)	7.4% (4.0-11.5)	6.9% (3.7-10.9)	7.1% (3.7-11.2)	6.8% (3.7-10.6)	7.2% (3.8-11.3)	7.3% (3.9-11.6)	6.9% (3.6-10.9)

	Main Analyses	Assortative Mixing	Higher RR in Europe	Decreasing incidence	Increasing incidence	All PWID start injecting stably housed	Treat 10% infected PWID per year	Unstably housed PWID have higher mortality rates
Cyprus*	7.0% (3.8-11.1)	7.2% (4.0-11.2)	7.0% (3.8-11.1)	7.1% (3.9-11.4)	6.8% (3.6-10.8)	7.2% (3.8-11.5)	7.5% (4.0-11.9)	7.0% (3.8-11.1)
Egypt*	6.9% (3.8-11.1)	7.2% (3.9-11.7)	6.9% (3.8-11.1)	7.1% (3.8-11.3)	6.8% (3.7-10.8)	7.2% (3.9-11.5)	7.5% (4.1-11.5)	6.9% (3.8-11.0)
Iraq*	7.1% (4.0-11.2)	7.4% (4.2-11.9)	7.1% (4.0-11.2)	7.2% (3.9-11.5)	6.9% (3.8-11.1)	7.3% (4.1-11.7)	7.5% (4.1-11.9)	7.0% (3.9-11.2)
Israel	6.1% (3.7-9.5)	6.8% (4.1-10.3)	6.1% (3.7-9.5)	6.2% (3.7-9.7)	6.1% (3.6-9.4)	6.3% (3.8-9.8)	6.4% (3.8-10.1)	6.1% (3.7-9.5)
Jordan*	7.0% (3.7-10.9)	7.4% (4.0-11.7)	7.0% (3.7-10.9)	7.2% (3.8-11.1)	6.9% (3.7-10.8)	7.3% (3.9-11.4)	7.4% (3.9-11.6)	7.0% (3.7-10.9)
Kuwait*	6.9% (3.8-11.0)	7.3% (4.1-11.5)	6.9% (3.8-11.0)	7.1% (3.8-11.2)	6.8% (3.8-10.9)	7.1% (3.9-11.5)	7.4% (4.0-11.8)	6.9% (3.8-11.1)
Lebanon*	8.3% (4.8-13.1)	12.3% (7.1-19.0)	8.3% (4.8-13.1)	8.2% (4.5-13.1)	8.4% (4.7-13.5)	8.8% (5.0-14.3)	8.4% (4.8-13.4)	8.3% (4.7-13.3)
Libyan Arab Jamahiriya*	3.4% (1.7-5.7)	2.9% (1.5-4.9)	3.4% (1.7-5.7)	3.7% (1.9-6.3)	3.0% (1.5-5.2)	3.0% (1.5-5.3)	4.6% (2.0-7.7)	3.4% (1.8-5.6)
Morocco	7.7% (3.7-12.5)	7.8% (3.6-13.3)	7.7% (3.7-12.5)	7.9% (3.7-12.9)	7.5% (3.6-12.5)	7.8% (3.8-13.2)	8.3% (3.9-13.4)	7.7% (3.8-12.7)
Occ. Palestinian Territories*	7.0% (3.8-11.0)	8.0% (4.4-12.8)	7.0% (3.8-11.0)	7.1% (3.7-11.2)	7.0% (3.7-11.1)	7.3% (3.9-11.4)	7.3% (3.9-11.4)	7.0% (3.8-11.1)
Oman*	6.8% (3.8-11.1)	7.2% (4.1-11.9)	6.8% (3.8-11.1)	7.1% (3.9-11.3)	6.7% (3.7-10.8)	7.0% (3.9-11.6)	7.2% (4.0-11.7)	6.9% (3.8-11.1)
Qatar*	6.9% (3.6-11.0)	7.4% (3.9-11.6)	6.9% (3.6-11.0)	7.0% (3.6-11.2)	6.8% (3.6-10.9)	7.1% (3.8-11.6)	7.4% (3.9-11.8)	6.9% (3.6-11.1)
Saudi Arabia*	4.9% (2.8-7.8)	4.4% (2.5-7.0)	4.9% (2.8-7.8)	5.3% (3.0-8.4)	4.6% (2.5-7.3)	4.8% (2.7-7.7)	5.9% (3.0-9.3)	4.9% (2.8-7.8)
South Sudan*	6.9% (3.8-11.2)	7.3% (4.0-11.6)	6.9% (3.8-11.2)	7.0% (4.1-11.5)	6.8% (3.6-10.9)	7.1% (3.9-11.5)	7.3% (3.9-11.8)	6.9% (3.8-11.2)
Sudan*	6.8% (3.7-11.1)	7.3% (4.0-12.1)	6.8% (3.7-11.1)	7.0% (3.6-11.3)	6.8% (3.7-11.0)	7.2% (3.9-11.6)	7.4% (3.9-11.9)	6.9% (3.7-11.3)
Syrian Arab Rep.*	9.0% (5.0-14.5)	28.4% (20.3-36.9)	9.0% (5.0-14.5)	8.8% (4.6-14.2)	9.3% (5.0-15.4)	9.8% (5.4-16.1)	9.0% (5.0-14.5)	9.0% (4.9-14.7)
Tunisia	4.4% (2.7-6.5)	6.1% (3.9-8.9)	4.4% (2.7-6.5)	4.5% (2.4-6.8)	4.4% (2.5-6.6)	4.6% (2.9-7.1)	4.4% (2.8-6.7)	4.3% (2.7-6.5)
Turkey*	8.3% (4.5-13.4)	8.3% (4.7-13.2)	8.3% (4.5-13.4)	8.7% (4.6-14.3)	7.8% (4.2-12.6)	8.9% (4.7-14.6)	8.8% (4.7-14.1)	8.3% (4.5-13.3)
United Arab Emirates*	7.0% (3.8-10.9)	7.3% (4.0-11.6)	7.0% (3.8-10.9)	7.2% (3.8-11.3)	6.8% (3.7-10.9)	7.2% (4.0-11.4)	7.4% (4.0-11.7)	6.9% (3.8-11.0)
Yemen*	6.9% (3.6-11.2)	7.2% (3.9-11.9)	6.9% (3.6-11.2)	7.0% (3.6-11.4)	6.7% (3.6-11.1)	7.1% (3.8-11.6)	7.3% (3.9-11.8)	6.9% (3.7-11.2)
**North America**								
Canada	21.6% (15.4-28.8)	20.2% (14.3-27.0)	21.6% (15.4-28.8)	21.8% (15.5-29.2)	21.2% (15.0-28.4)	21.6% (15.6-28.9)	23.4% (16.9-31.0)	21.6% (15.4-29.1)
United States	27.0% (20.1-34.3)	26.0% (19.5-33.0)	27.0% (20.1-34.3)	27.0% (20.0-34.4)	26.8% (20.2-34.3)	27.1% (20.4-34.7)	28.4% (21.3-35.8)	27.3% (20.5-34.7)
**South Asia**								
Afghanistan	26.0% (17.9-36.3)	23.5% (16.8-31.5)	26.0% (17.9-36.3)	26.8% (18.6-38.0)	24.9% (17.1-34.2)	28.1% (19.1-40.1)	26.8% (18.5-37.1)	26.1% (17.8-36.6)
Bangladesh	12.4% (5.7-20.6)	14.1% (7.0-22.8)	12.4% (5.7-20.6)	12.4% (5.7-20.4)	12.3% (5.7-20.3)	13.3% (6.0-22.0)	12.8% (5.9-21.1)	12.4% (5.7-20.5)
Bhutan*	21.3% (13.8-30.3)	21.3% (13.7-30.0)	21.3% (13.8-30.3)	21.3% (13.9-30.1)	21.0% (13.5-30.4)	22.3% (14.3-32.0)	22.1% (14.8-30.9)	21.4% (14.0-30.7)
India	28.2% (18.5-38.8)	26.9% (18.7-34.9)	28.2% (18.5-38.8)	28.2% (18.7-38.6)	28.2% (18.4-38.5)	29.1% (19.3-40.0)	29.1% (19.2-39.9)	28.7% (18.8-39.2)
Iran	21.8% (15.3-29.7)	21.6% (15.0-29.2)	21.8% (15.3-29.7)	21.9% (15.3-29.7)	21.7% (15.1-29.6)	22.5% (15.6-30.6)	22.8% (16.0-30.8)	22.1% (15.5-30.3)
Maldives*	32.6% (22.3-46.1)	38.7% (30.9-45.4)	32.6% (22.3-46.1)	30.4% (20.9-42.4)	34.7% (23.6-49.6)	35.7% (23.9-51.7)	32.6% (22.3-46.1)	32.8% (22.5-46.0)
Nepal	1.1% (0.5-2.0)	1.2% (0.5-2.2)	1.1% (0.5-2.0)	1.3% (0.1-2.5)	1.0% (-0.0-2.0)	1.2% (0.5-2.1)	1.1% (0.3-2.1)	1.1% (0.5-1.9)
Pakistan	18.8% (11.5-28.3)	19.6% (10.4-33.6)	18.8% (11.5-28.3)	19.0% (12.1-27.9)	18.6% (10.8-29.2)	19.9% (11.6-31.1)	19.6% (12.7-28.7)	18.9% (11.6-28.5)
Sri Lanka*	2.0% (1.0-3.7)	2.5% (1.1-5.2)	2.0% (1.0-3.7)	2.1% (0.7-4.0)	2.0% (0.7-3.8)	2.1% (1.0-3.9)	2.1% (0.8-3.9)	2.0% (0.9-3.7)
**Sub Saharan Africa**								
Angola*	24.3% (13.8-36.1)	26.8% (17.9-35.5)	24.3% (13.8-36.1)	23.7% (13.6-35.2)	24.7% (13.7-36.7)	26.2% (15.0-39.3)	24.6% (14.0-36.4)	24.4% (14.0-36.3)
Benin*	19.3% (10.9-28.4)	24.3% (15.8-32.4)	19.3% (10.9-28.4)	18.9% (10.7-27.8)	19.6% (11.1-28.7)	20.1% (11.4-29.2)	19.4% (11.1-28.6)	19.6% (11.2-28.3)
Botswana*	24.5% (14.6-35.1)	27.1% (18.3-35.3)	24.5% (14.6-35.1)	23.9% (14.2-34.3)	25.0% (14.4-35.8)	26.2% (15.7-38.2)	24.8% (14.8-35.5)	24.7% (14.6-35.7)
Burkina Faso*	24.7% (14.3-36.2)	27.1% (18.2-35.5)	24.7% (14.3-36.2)	24.1% (13.7-35.3)	25.2% (14.4-37.1)	26.6% (15.4-39.4)	25.0% (14.5-36.5)	25.0% (14.4-36.6)
Burundi*	24.6% (13.9-36.8)	27.2% (18.2-35.7)	24.6% (13.9-36.8)	24.0% (13.8-36.2)	25.0% (14.2-37.4)	26.5% (15.0-39.8)	24.9% (14.1-37.1)	24.8% (14.1-37.3)
Cameroon*	24.1% (14.4-37.3)	26.7% (18.3-35.9)	24.1% (14.4-37.3)	23.5% (13.9-36.2)	24.5% (14.7-37.6)	26.2% (15.5-40.3)	24.5% (14.6-37.6)	24.2% (14.5-37.4)
Cape Verde*	24.3% (13.9-37.0)	27.1% (18.0-35.9)	24.3% (13.9-37.0)	23.8% (13.4-36.5)	24.7% (14.4-38.0)	26.2% (15.2-40.4)	24.6% (14.2-37.4)	24.6% (14.1-38.0)
Central African Republic*	24.3% (14.2-37.1)	26.9% (18.2-35.7)	24.3% (14.2-37.1)	23.7% (13.7-36.3)	24.9% (14.3-37.6)	26.2% (15.5-39.4)	24.6% (14.3-37.5)	24.5% (14.1-37.2)
Chad*	24.4% (14.3-37.0)	27.0% (18.3-36.0)	24.4% (14.3-37.0)	23.9% (14.0-36.1)	24.9% (14.4-37.6)	26.3% (15.3-39.7)	24.7% (14.5-37.3)	24.7% (14.3-37.5)

	Main Analyses	Assortative Mixing	Higher RR in Europe	Decreasing incidence	Increasing incidence	All PWID start injecting stably housed	Treat 10% infected PWID per year	Unstably housed PWID have higher mortality rates
Comoros*	24.4% (14.3-37.1)	27.0% (18.2-36.1)	24.4% (14.3-37.1)	23.7% (13.7-36.2)	24.9% (14.6-37.7)	26.2% (15.6-39.6)	24.7% (14.6-37.5)	24.6% (14.4-37.4)
Congo (Kinshasa)*	24.7% (14.4-35.9)	27.3% (18.2-35.2)	24.7% (14.4-35.9)	24.1% (13.9-35.1)	25.1% (14.6-36.4)	26.6% (15.5-39.3)	24.9% (14.6-36.2)	24.9% (14.6-36.5)
Cote d'Ivoire	14.4% (6.8-25.5)	32.9% (22.7-42.4)	14.4% (6.8-25.5)	13.7% (6.1-23.9)	15.2% (7.0-27.1)	16.3% (7.5-28.9)	14.5% (6.8-25.5)	14.5% (6.9-25.9)
Djibouti*	24.4% (14.5-36.3)	27.1% (18.0-35.6)	24.4% (14.5-36.3)	23.9% (13.8-35.8)	24.8% (14.5-36.7)	26.3% (15.6-39.2)	24.7% (14.6-36.6)	24.6% (14.4-36.8)
Equatorial Guinea*	24.6% (14.5-37.0)	27.2% (18.3-36.0)	24.6% (14.5-37.0)	24.1% (14.1-35.8)	25.0% (14.6-37.6)	26.4% (16.0-40.1)	24.9% (14.8-37.5)	24.8% (14.5-37.4)
Eritrea*	24.4% (14.3-37.0)	26.9% (18.3-35.7)	24.4% (14.3-37.0)	23.8% (14.1-36.1)	24.8% (14.7-37.3)	26.3% (15.4-40.0)	24.7% (14.5-37.3)	24.5% (14.7-36.8)
Ethiopia*	24.2% (14.0-37.0)	26.8% (17.8-35.5)	24.2% (14.0-37.0)	23.7% (13.6-36.2)	24.6% (14.0-37.4)	26.0% (15.4-39.6)	24.5% (14.2-37.3)	24.5% (14.1-37.2)
Gabon*	24.5% (14.0-36.2)	27.1% (18.0-35.3)	24.5% (14.0-36.2)	23.9% (13.9-35.5)	24.9% (14.2-36.9)	26.4% (15.1-39.2)	24.8% (14.2-36.6)	24.8% (14.2-36.6)
Gambia*	24.5% (14.2-36.3)	27.2% (18.2-35.4)	24.5% (14.2-36.3)	24.0% (13.9-35.1)	25.0% (14.3-36.9)	26.6% (15.3-39.6)	24.8% (14.4-36.6)	24.8% (14.3-36.7)
Ghana*	18.3% (10.5-27.7)	19.2% (11.7-27.2)	18.3% (10.5-27.7)	18.3% (10.4-27.6)	18.2% (10.5-27.5)	18.9% (10.9-28.5)	18.9% (10.8-28.4)	18.5% (10.4-28.2)
Guinea*	24.3% (14.5-36.3)	26.8% (18.4-35.7)	24.3% (14.5-36.3)	23.7% (14.1-35.9)	24.6% (14.7-36.7)	26.0% (15.7-39.1)	24.6% (14.7-36.7)	24.5% (14.5-36.8)
Guinea-Bissau*	24.5% (14.4-36.1)	27.1% (18.2-35.3)	24.5% (14.4-36.1)	23.9% (14.0-35.4)	24.9% (14.3-36.6)	26.3% (15.8-38.9)	24.8% (14.6-36.4)	24.8% (14.6-36.5)
Kenya	23.4% (17.1-31.8)	28.5% (21.6-35.9)	23.4% (17.1-31.8)	22.7% (16.4-31.1)	24.1% (17.5-32.7)	25.2% (17.9-35.1)	23.6% (17.2-32.0)	23.7% (17.2-31.8)
Lesotho*	24.2% (13.6-36.9)	27.0% (17.6-35.4)	24.2% (13.6-36.9)	23.8% (13.0-36.0)	24.7% (13.8-37.2)	26.2% (14.8-39.8)	24.5% (13.8-37.2)	24.5% (13.6-37.1)
Liberia*	24.4% (14.5-35.5)	26.9% (18.4-35.5)	24.4% (14.5-35.5)	23.8% (14.1-34.8)	24.9% (14.7-36.1)	26.3% (15.6-38.5)	24.7% (14.6-36.0)	24.5% (14.7-35.7)
Madagascar*	26.7% (15.5-40.2)	35.2% (27.2-42.9)	26.7% (15.5-40.2)	25.5% (14.9-38.1)	28.0% (16.4-42.3)	29.2% (17.1-43.8)	26.7% (15.5-40.3)	26.9% (15.4-40.4)
Malawi*	24.3% (14.1-37.4)	27.1% (18.2-36.1)	24.3% (14.1-37.4)	23.8% (13.9-36.7)	24.8% (14.3-38.0)	26.2% (15.5-40.0)	24.6% (14.3-37.8)	24.6% (14.2-37.7)
Mali*	24.8% (14.7-36.1)	27.3% (18.7-35.3)	24.8% (14.7-36.1)	24.2% (14.1-35.2)	25.4% (15.1-36.5)	26.7% (15.8-38.9)	25.1% (14.9-36.4)	25.2% (14.7-36.5)
Mauritania*	24.2% (14.5-36.3)	26.6% (18.5-35.4)	24.2% (14.5-36.3)	23.6% (14.2-36.0)	24.5% (14.5-36.4)	26.0% (15.6-39.8)	24.4% (14.7-36.6)	24.3% (14.4-36.6)
Mauritius*	9.4% (4.8-14.7)	8.2% (4.1-12.9)	9.4% (4.8-14.7)	10.2% (5.3-15.7)	8.6% (4.4-13.7)	8.5% (4.2-14.0)	12.5% (6.5-19.1)	9.3% (4.8-14.5)
Mozambique*	15.0% (8.6-23.3)	13.2% (7.6-20.0)	15.0% (8.6-23.3)	16.2% (9.2-24.9)	13.9% (7.8-21.7)	14.9% (8.5-23.0)	16.8% (9.6-25.5)	15.0% (8.6-23.2)
Namibia*	24.4% (14.8-36.9)	26.9% (18.7-35.9)	24.4% (14.8-36.9)	23.8% (14.2-36.2)	24.7% (14.8-37.9)	26.3% (15.8-39.7)	24.6% (15.0-37.3)	24.5% (14.8-37.6)
Niger*	24.5% (14.2-36.6)	27.1% (18.1-35.5)	24.5% (14.2-36.6)	24.0% (13.9-35.8)	24.8% (14.6-37.3)	26.3% (15.4-39.6)	24.7% (14.5-36.9)	24.6% (14.4-37.0)
Nigeria*	22.5% (13.3-33.6)	33.6% (25.5-41.1)	22.5% (13.3-33.6)	21.7% (12.8-32.1)	23.3% (13.7-35.0)	23.9% (14.1-35.8)	22.5% (13.3-33.6)	22.8% (13.6-34.3)
Rep. of the Congo*	24.3% (14.1-36.6)	27.0% (18.3-35.4)	24.3% (14.1-36.6)	23.8% (13.6-36.0)	24.7% (14.5-37.2)	26.4% (15.3-39.9)	24.7% (14.3-36.9)	24.5% (14.2-36.8)
Rwanda*	24.3% (13.7-36.0)	26.9% (18.1-35.5)	24.3% (13.7-36.0)	23.8% (13.5-34.9)	24.7% (13.9-37.0)	26.2% (15.0-38.9)	24.5% (14.0-36.4)	24.5% (13.9-36.6)
Sao Tome & Principe*	24.6% (14.5-37.2)	27.2% (18.5-35.7)	24.6% (14.5-37.2)	24.0% (14.2-36.2)	25.0% (14.7-37.5)	26.4% (15.6-40.3)	24.9% (14.7-37.6)	24.8% (14.7-37.2)
Senegal*	21.1% (11.5-31.8)	20.7% (12.1-29.4)	21.1% (11.5-31.8)	21.4% (11.6-31.9)	20.8% (11.2-31.1)	22.4% (12.2-33.6)	22.0% (12.1-32.9)	21.3% (11.5-32.0)
Seychelles*	20.8% (12.1-31.9)	20.0% (12.4-28.6)	20.8% (12.1-31.9)	20.9% (12.1-32.1)	20.3% (11.7-31.2)	21.9% (12.7-33.0)	21.6% (12.5-32.9)	20.8% (12.0-32.0)
Sierra Leone*	24.8% (14.3-35.9)	27.5% (18.6-35.0)	24.8% (14.3-35.9)	24.3% (13.8-35.6)	25.3% (14.3-36.3)	26.8% (15.7-38.9)	25.0% (14.6-36.2)	25.2% (14.3-36.4)
Somalia*	24.5% (14.0-36.5)	27.1% (17.8-35.6)	24.5% (14.0-36.5)	24.0% (13.6-35.6)	24.8% (14.5-37.0)	26.3% (15.2-39.4)	24.7% (14.3-36.9)	24.6% (14.1-36.9)
South Africa*	33.1% (21.7-46.1)	31.8% (23.4-39.8)	33.1% (21.7-46.1)	32.2% (21.2-45.1)	33.7% (22.1-46.9)	34.9% (23.0-48.4)	33.4% (21.9-46.4)	33.6% (21.9-46.3)
Swaziland*	24.5% (13.9-37.4)	27.2% (18.1-36.2)	24.5% (13.9-37.4)	23.8% (13.7-36.2)	24.9% (14.2-37.3)	26.3% (15.3-39.7)	24.7% (14.1-37.7)	24.7% (14.1-37.1)
Togo*	14.1% (9.0-20.7)	18.8% (13.0-25.6)	14.1% (9.0-20.7)	13.8% (8.8-20.2)	14.2% (9.2-21.0)	15.6% (9.7-22.9)	14.2% (9.1-21.0)	14.1% (9.2-20.7)
Uganda*	24.4% (14.5-36.2)	27.0% (18.2-35.4)	24.4% (14.5-36.2)	23.9% (13.9-35.1)	24.9% (14.5-36.8)	26.2% (15.3-39.1)	24.7% (14.6-36.5)	24.6% (14.6-36.3)
United Rep. of Tanzania	21.8% (12.1-34.0)	23.2% (14.4-32.5)	21.8% (12.1-34.0)	21.5% (12.1-33.4)	21.7% (12.2-33.8)	23.5% (13.1-36.4)	22.1% (12.3-34.4)	21.9% (12.0-34.2)
Zambia*	24.6% (14.4-36.3)	27.0% (18.3-35.6)	24.6% (14.4-36.3)	24.1% (14.0-35.4)	25.0% (14.8-36.7)	26.5% (15.3-38.8)	25.0% (14.6-36.6)	24.7% (14.6-36.3)
Zimbabwe*	24.7% (14.5-35.5)	27.1% (18.6-35.2)	24.7% (14.5-35.5)	24.2% (14.3-34.6)	25.2% (14.7-36.0)	26.6% (15.8-38.4)	25.0% (14.6-35.8)	24.9% (14.8-36.0)
**Western Europe**								
Albania	18.9% (13.0-25.3)	21.2% (15.0-27.6)	27.8% (18.5-37.6)	18.7% (12.9-25.3)	19.0% (13.1-25.7)	19.6% (13.4-26.4)	19.3% (13.3-25.8)	19.0% (13.2-25.9)
Andorra*	13.9% (9.4-19.1)	13.9% (9.5-18.9)	20.8% (14.0-28.9)	14.0% (9.7-19.2)	13.6% (9.3-18.9)	14.2% (9.5-19.4)	14.8% (10.1-20.4)	13.9% (9.5-19.0)
Austria	9.4% (5.8-13.7)	9.1% (5.7-13.4)	14.2% (8.9-21.5)	9.5% (5.9-14.0)	9.2% (5.7-13.4)	9.4% (5.9-13.8)	10.2% (6.3-14.8)	9.4% (5.9-13.7)
Belgium	17.7% (10.7-24.8)	17.2% (10.6-24.1)	26.1% (15.7-36.4)	17.9% (10.9-25.1)	17.5% (10.4-24.6)	17.8% (10.8-25.1)	19.0% (11.4-26.6)	17.7% (10.7-25.1)
Croatia	11.3% (6.7-16.8)	13.3% (8.2-19.2)	17.3% (10.1-25.4)	11.3% (6.7-16.8)	11.3% (6.8-16.9)	11.6% (6.9-17.6)	11.7% (6.9-17.4)	11.3% (6.8-17.0)
Denmark	17.0% (10.4-25.1)	18.3% (11.6-26.5)	25.4% (14.6-36.1)	16.9% (10.4-25.1)	17.0% (10.4-25.4)	17.4% (10.6-25.8)	17.6% (10.8-25.9)	17.2% (10.5-25.4)

	Main Analyses	Assortative Mixing	Higher RR in Europe	Decreasing incidence	Increasing incidence	All PWID start injecting stably housed	Treat 10% infected PWID per year	Unstably housed PWID have higher mortality rates
England	26.0% (18.2-34.6)	27.4% (20.2-34.5)	36.8% (25.7-48.6)	25.7% (17.9-34.2)	26.3% (18.4-35.1)	26.8% (18.8-35.5)	26.5% (18.4-35.2)	26.2% (18.2-34.6)
Finland*	11.4% (7.9-15.9)	10.4% (7.1-14.4)	17.2% (11.6-24.6)	11.8% (8.3-16.3)	10.9% (7.5-15.3)	11.2% (7.8-15.6)	13.1% (8.9-18.1)	11.4% (7.9-15.8)
Form. Yug. Rep. Macedonia*	12.9% (8.9-17.9)	12.4% (8.4-17.1)	19.5% (13.2-27.6)	13.3% (9.2-18.3)	12.6% (8.7-17.5)	13.0% (9.0-18.0)	14.2% (9.7-19.7)	12.9% (8.9-17.9)
France	6.8% (4.4-10.3)	6.5% (4.2-9.9)	10.8% (6.9-16.1)	7.1% (4.6-10.7)	6.6% (4.2-10.1)	6.9% (4.5-10.4)	7.7% (4.8-11.5)	6.8% (4.4-10.4)
Germany	8.7% (6.0-12.2)	8.3% (5.6-11.7)	13.5% (8.8-19.4)	8.9% (6.2-12.3)	8.4% (5.9-12.0)	8.7% (6.0-12.4)	9.6% (6.4-13.5)	8.7% (6.0-12.2)
Greece	16.4% (10.5-22.1)	15.3% (10.0-20.8)	24.0% (16.3-33.2)	16.8% (10.8-22.5)	16.0% (10.3-21.6)	16.4% (10.6-21.9)	18.0% (11.7-24.1)	16.4% (10.6-22.1)
Greenland*	13.9% (9.7-19.1)	13.9% (9.9-18.7)	20.8% (14.1-29.2)	14.1% (9.8-19.3)	13.7% (9.7-18.7)	14.2% (9.8-19.3)	14.8% (10.4-20.1)	14.0% (9.8-19.0)
Iceland	20.8% (15.2-27.1)	18.9% (13.8-24.6)	30.3% (22.1-39.2)	21.5% (15.6-28.1)	20.1% (14.6-26.3)	21.0% (15.3-27.2)	22.8% (16.8-29.5)	21.0% (15.2-27.2)
Ireland	3.0% (1.9-4.6)	2.7% (1.7-4.1)	4.7% (2.9-7.5)	3.2% (2.0-4.9)	2.8% (1.7-4.3)	3.0% (1.8-4.5)	3.6% (1.9-5.8)	3.0% (1.9-4.5)
Italy	9.6% (6.9-12.7)	9.3% (6.7-12.4)	14.7% (10.3-20.2)	9.9% (7.1-13.0)	9.4% (6.7-12.4)	9.8% (7.0-13.0)	10.5% (7.4-13.8)	9.6% (6.9-12.7)
Liechtenstein*	14.0% (9.7-19.2)	14.0% (9.7-18.9)	20.9% (14.1-29.3)	14.2% (9.8-19.5)	13.7% (9.6-19.0)	14.2% (9.9-19.6)	14.8% (10.4-20.4)	14.0% (9.7-19.4)
Luxembourg*	10.3% (7.2-14.7)	9.2% (6.4-13.2)	15.9% (10.4-22.8)	10.9% (7.5-15.3)	9.7% (6.8-14.1)	9.9% (6.9-14.3)	12.3% (8.3-17.4)	10.3% (7.2-14.7)
Malta*	16.3% (11.2-22.5)	20.7% (14.3-27.8)	24.0% (16.8-33.9)	16.0% (11.2-22.0)	16.6% (11.3-22.7)	17.0% (11.5-23.4)	16.5% (11.3-22.8)	16.4% (11.3-22.6)
Monaco*	14.0% (9.6-19.3)	14.0% (9.6-19.0)	21.1% (13.9-29.6)	14.2% (9.6-19.6)	13.8% (9.4-18.9)	14.3% (9.7-19.7)	14.9% (10.0-20.5)	14.1% (9.6-19.4)
Montenegro*	16.3% (11.1-22.6)	16.6% (11.6-22.3)	24.5% (16.3-33.9)	16.5% (11.2-22.9)	16.1% (11.0-22.1)	17.1% (11.6-23.7)	17.1% (11.7-23.5)	16.4% (11.1-22.6)
Netherlands	8.7% (6.0-11.6)	8.6% (5.9-11.7)	13.3% (9.1-18.8)	8.9% (6.0-11.9)	8.5% (5.8-11.4)	8.9% (6.1-11.9)	9.3% (6.3-12.5)	8.7% (5.9-11.6)
Northern Ireland*	13.9% (9.6-19.2)	13.9% (9.6-19.0)	20.9% (14.3-29.3)	14.1% (9.8-19.4)	13.8% (9.3-18.9)	14.2% (9.7-19.5)	14.8% (10.1-20.4)	14.0% (9.5-19.1)
Norway	15.0% (9.7-20.8)	14.3% (9.2-19.9)	22.2% (14.4-31.8)	15.3% (10.0-21.2)	14.7% (9.5-20.6)	15.0% (9.6-21.0)	16.5% (10.6-23.0)	15.0% (9.6-21.0)
Portugal*	9.2% (6.1-13.2)	8.1% (5.3-11.7)	14.2% (9.3-20.7)	9.9% (6.6-14.1)	8.7% (5.6-12.5)	8.7% (5.7-12.8)	11.5% (7.8-16.1)	9.2% (6.1-13.3)
San Marino*	13.8% (9.4-19.4)	13.8% (9.4-19.1)	20.8% (13.9-28.7)	14.0% (9.3-19.5)	13.6% (9.2-19.1)	14.1% (9.5-19.9)	14.8% (10.0-20.8)	13.9% (9.3-19.5)
Scotland	15.6% (11.2-20.4)	15.6% (11.3-20.5)	23.0% (16.2-31.1)	15.7% (11.2-20.5)	15.4% (11.0-20.4)	15.8% (11.4-21.2)	16.5% (11.8-21.7)	15.6% (11.2-20.7)
Serbia	1.0% (0.4-1.8)	1.6% (0.6-3.0)	1.7% (0.5-3.3)	1.2% (-0.2-2.6)	1.0% (-0.3-2.3)	1.1% (0.4-2.0)	1.0% (0.3-1.9)	1.0% (0.4-1.9)
Slovenia	6.5% (3.8-10.6)	8.8% (5.2-13.7)	10.4% (6.1-16.5)	6.5% (3.7-10.5)	6.6% (3.7-10.6)	6.9% (4.0-11.3)	6.7% (3.9-10.8)	6.5% (3.8-10.6)
Spain	13.3% (8.6-19.1)	12.3% (7.9-17.5)	19.8% (12.9-28.7)	13.8% (9.0-19.8)	12.9% (8.2-18.5)	13.3% (8.6-19.1)	15.0% (9.7-21.5)	13.4% (8.6-19.1)
Sweden	16.3% (10.6-23.5)	14.9% (9.7-21.3)	24.0% (15.9-33.8)	16.8% (11.0-24.0)	15.9% (10.4-22.9)	16.0% (10.4-22.9)	18.8% (12.2-26.6)	16.2% (10.7-23.2)
Switzerland	7.0% (5.0-9.3)	6.4% (4.5-8.5)	10.8% (7.4-15.4)	7.3% (5.2-9.7)	6.7% (4.7-9.0)	6.9% (4.9-9.2)	8.1% (5.7-11.1)	7.0% (5.0-9.4)
Wales	30.6% (22.9-38.6)	31.0% (23.9-37.7)	41.7% (31.8-52.2)	30.1% (22.5-37.9)	31.0% (23.3-39.1)	31.5% (23.4-39.6)	31.0% (23.2-39.0)	30.9% (23.0-38.6)

Appendix Figure 5: Sensitivity analyses. *Red bars show the median global transmission PAF (tPAF) or classical PAF (cPAF) of unstable housing for HIV (top) and HCV (bottom) for each of the sensitivity analyses. The solid black lines show the median baseline tPAF.*

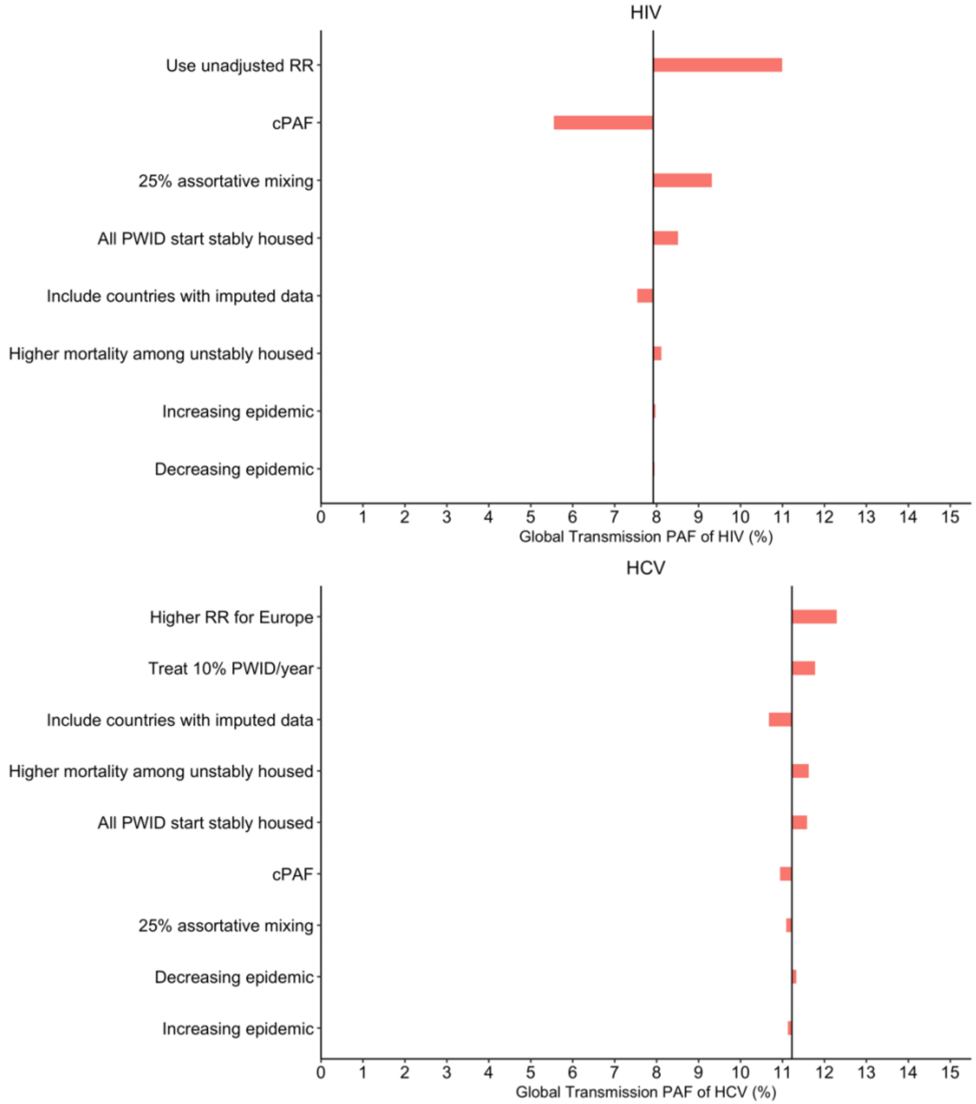

References

1. Degenhardt L, Peacock A, Colledge S, et al. Global prevalence of injecting drug use and sociodemographic characteristics and prevalence of HIV, HBV, and HCV in people who inject drugs: a multistage systematic review. *Lancet Glob Health* 2017; **5**(12): e1192-e207.
2. Hines LA, Trickey A, Leung J, et al. Associations between national development indicators and the age profile of people who inject drugs: results from a global systematic review and meta-analysis. *Lancet Glob Health* 2020; **8**(1): e76-e91.
3. Mumtaz GR, Weiss HA, Thomas SL, et al. HIV among people who inject drugs in the Middle East and North Africa: systematic review and data synthesis. *PLoS Med* 2014; **11**(6): e1001663.
4. Arum C, Fraser H, Artenie AA, et al. Homelessness, unstable housing, and risk of HIV and hepatitis C virus acquisition among people who inject drugs: a systematic review and meta-analysis. *The Lancet Public Health* 2021; **6**(5): e309-e23.
